# Discovery of *N*-aryl sulphonamide-quinazoline derivatives as anti-gastric cancer agents *in vitro* and *in vivo via* activating the Hippo signalling pathway

**DOI:** 10.1080/14756366.2021.1958211

**Published:** 2021-08-23

**Authors:** Jin-Bo Niu, Chun-Quan Hua, Yuan Liu, Guang-Xi Yu, Jia-Jia Yang, Yin-Ru Li, Yan-Bing Zhang, Ying-Qiu Qi, Jian Song, Cheng-Yun Jin, Sai-Yang Zhang

**Affiliations:** a The Third Affiliated Hospital of Zhengzhou University, Zhengzhou, China; b School of Pharmaceutical Sciences, Institute of Drug Discovery & Development, Key Laboratory of Advanced Drug Preparation Technologies (Ministry of Education), Zhengzhou University, Zhengzhou, China; c School of Basic Medical Sciences, Zhengzhou University, Zhengzhou, China; d Department of Pharmacy, Zhengzhou People's Hospital, Zhengzhou, China; e Henan Institute of Advanced Technology, Zhengzhou University, Zhengzhou, China

**Keywords:** Hippo signalling pathway, YAP, *N*-aryl sulphonamide-quinazoline, anti-proliferative activity

## Abstract

Hippo signalling pathway plays a crucial role in tumorigenesis and cancer progression. In this work, we identified an *N-aryl sulphonamide-quinazoline derivative*, compound **9i** as an anti-gastric cancer agent, which exhibited potent antiproliferative ability with IC_50_ values of 0.36 μM (MGC-803 cells), 0.70 μM (HCT-116 cells), 1.04 μM (PC-3 cells), and 0.81 μM (MCF-7 cells), respectively and inhibited YAP activity by the activation of p-LATS. Compound **9i** was effective in suppressing MGC-803 xenograft tumour growth in nude mice without obvious toxicity and significantly down-regulated the expression of YAP *in vivo*. Compound **9i** arrested cells in the G2/M phase, induced intrinsic apoptosis, and inhibited cell colony formation in MGC-803 and SGC-7901 cells. Therefore, compound **9i** is to be reported as an anti-gastric cancer agent *via* activating the Hippo signalling pathway and might help foster a new strategy for the cancer treatment by activating the Hippo signalling pathway regulatory function to inhibit the activity of YAP.

## 1. Introduction

Hippo signalling pathway is a highly conserved signalling cascade that initially was identified in *Drosophila*^1–3^. It was found to be a crucial regulator on cell survival, proliferation, and differentiation mammals in the last several years^4–9^. In mammal cells, the Hippo pathway contains protein kinases of Salvador homolog (SAV) 1, Mammalian STE20-like protein kinase (MST) 1/2, Large tumour suppressor (LATS) 1/2, Mps one binder kinase activator-like (MOB) 1. These proteins regulate downstream effectors of Yes-associated protein (YAP), transcriptional co-activator with PDZ-binding motif (TAZ), and transcriptional enhanced associate domain (TEAD)1–4. In the signalling cascade, phosphorylated MST1/2 could phosphorylate and activate LATS1/2. Phosphorylated LATS1/2 would phosphorylate YAP in the cytoplasm. SAV1 and MOB1, two scaffold proteins, could be activated by MST1/2. The phosphorylated YAP would be degraded via the ubiquitin-proteasome system^10^. Activated (non-phosphorylated) YAP would be translocated from the cytoplasm to the cell nuclear^11^^,^^12^. In cell nuclear, YAP together with TEADs regulates its target oncogene proteins like c-Myc and Bcl-2. YAP is over-activated in many cancers^5^^,^^13–15^ like breast cancer^16^, lung cancer^17^, colorectal cancer^18^, gastric cancer^19^, and prostate cancer^20^, and its negatively regulated oncogenic target gene expression is higher than that of normal cells^21^. Therefore Hippo/YAP is now believed as a promising target for cancer therapy, because of its close relation with tumour development^22–24^. However, there are few reported molecule modulators of the Hippo pathway and most of them did not act potent anticancer activity^25–30^.

**Figure 1.**
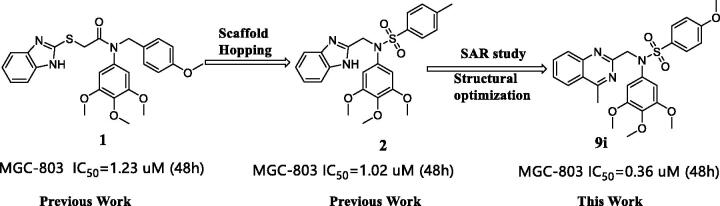
Design of N-aryl sulphonamide-quinazoline derivative **9i** as an anticancer agent in this work .

In this study, we reported the identification of a novel Hippo activator, compound **9i** (Figure 1), based on unexpected findings from our previous work from an experimental screening and the SAR study of our reported compound **1**^31^ and compound **2**^32^. Compound **1** was a tubulin inhibitor against MGC-803 cells with IC_50_ value at the micromolar level and Compound **2** inhibited the AKT/mTOR and RAS/Raf/MEK/ERK pathways against MGC-803 cells with IC_50_ value at the micromolar level. In our work, compound **9i** exhibited high antiproliferative ability against MGC-803 cells with IC_50_ value at nanomolar level, it lacked compound **1**’s ability to inhibit tubulin polymerisation and compound **2**’s ability to inhibit the AKT/mTOR and RAS/Raf/MEK/ERK pathways (these results are provided in the Supporting Information). Fortunately, we found compound **9i** showed an obvious activation activity of Hippo pathway. Compound **9i** inhibited YAP activity by the activation of p-LATS and induced the expression of p-YAP. Equally important, compound **9i** was effective in suppressing MGC-803 xenograft tumour growth in nude mice without obvious toxicity and also significantly down-regulated YAP *in vivo*. Therefore, we here reported *N*-aryl sulphonamide-quinazoline derivatives as novel anti-gastric cancer agents via activating the Hippo signalling pathway *in vitro* and *in vivo*.

## 2. Chemistry

**Scheme 1.**
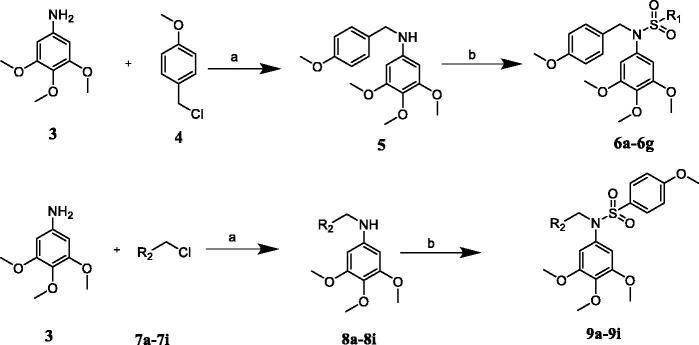
Synthesis of compounds **6a–6g** and **9a–9i**. Reagents and conditions: (a) DMF, K_2_CO_3_, rt, 8 h; (b) sulphonyl chloride derivatives or acyl chloride derivatives, TEA, DCM, rt, 5 h.

**Scheme 2.**
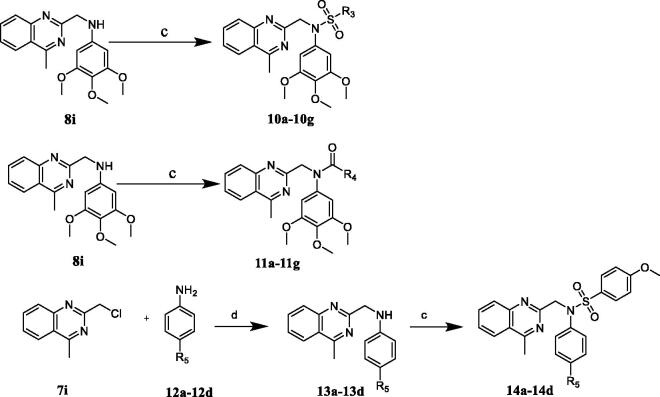
Synthesis of compounds **10a–10g**, **11a–11g**, and **14a–14d**. Reagents and conditions: (c) sulphonyl chlorides or acyl chlorides, TEA, DCM, rt, 5 h. (d) DMF, K_2_CO_3_, rt, 8 h.

Lead optimisation and SAR study were initiated with the aim of the compounds with potent antiproliferative ability. We designed and synthesised five series of *N*-aryl sulphonamide derivatives **6a–6g**, **9a–9i**, **10a–10j**, **11a–11j**, and **14a–14d**. Compounds were synthesised by outlined procedures in Schemes 1–2. Commercially available 3,4,5-trimethoxyaniline **3** reacted with substituted chloromethyl compounds **4** and **7a–7i** to give compounds **5** and **8a–8i**. Treatment of compounds **5** and **8a–8i** with sulphonyl chlorides in the presence of TEA in DCM furnished targeted compounds **6a–6g** and **9a–9i** (Scheme 1). Characterisation of compounds **6a–6g** and **9a–9i** was carried out by NMR and HREI-mass spectra.

*N*-aryl sulphonamide-quinazoline derivatives **10a–10g** and **11a–11g** were synthesised by the treatment of compound **8i** with sulphonyl chlorides or acyl chlorides in the presence of TEA in DCM (Scheme 2). The synthesis of quinazoline derivatives **14a–14g** involved two steps. Substitution reaction between commercially available 2-(chloromethyl)-4-methylquinazoline (**7i)** and aromatic amines **12a–12d** in the presence of K_2_CO_3_ in DMF gave corresponding intermediates **13a–13d**, followed by the treatment of sulphonyl chlorides, forming compounds **14a–14d** (Scheme 2). Characterisation of compounds **10a–10j**, **11a–11j**, and **14a–14d** were carried out by NMR and HREI-mass spectra.

## 3. Biological evaluation

### 3.1. In vitro antiproliferative activity

The *in vitro* antiproliferative activities of new target compounds **6a–6g**, **9a–9i**, **10a–10j**, **11a–11j**, and **14a–14d** were evaluated against four human cancer cell lines (MGC-803, PC-3, HCT-116, and MCF-7) using MTT assay and **5-FU** as a positive drug. The following Tables 1–4 depicted the results of *in vitro* antiproliferative activity.

**Table 1.** *In vitro* antiproliferative activity of compounds **6a–6g** against human cancer cells.

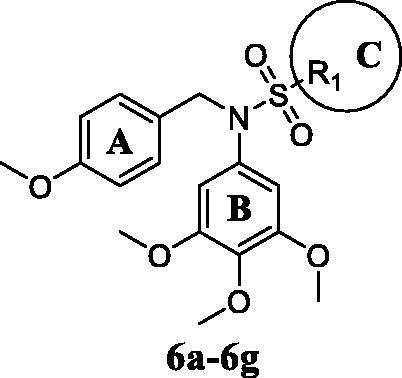					
Comp.	*R*_1_	IC_50_ (μM)^a^			
		MGC-803	HCT-116	PC-3	MCF-7
**6a**	4-F-phenyl	6.62 ± 0.82	16.7 ± 1.03	18.82 ± 0.31	>40
**6b**	4-Cl-phenyl	>40	>40	>40	>40
**6c**	4-CH_3_-phenyl	4.32 ± 0.65	6.18 ± 0.61	8.81 ± 0.98	11.24 ± 1.02
**6d**	Phenyl	>40	>40	>40	>40
**6e**	4-OCH_3_-phenyl	2.86 ± 1.08	3.48 ± 1.22	4.01 ± 1.67	7.68 ± 1.82
**6f**	2-thienyl	28.31 ± 2.86	38.12 ± 2.64	38.71 ± 2.87	26.11 ± 1.87
**6g**	3-pyridyl	38.23 ± 1.55	>40	16.88 ± 1.58	>40
**5-Fu**	–	6.82 ± 1.12	14.4 ± 1.73	17.1 ± 1.42	12.1 ± 1.28

^a^*In vitro* antiproliferative activity was assayed by exposure for 48 h.

According to our previous work, the sulphonyl groups are important for maintaining inhibitory activities. Therefore, we initially investigated the influence of the sulphonyl group part (C ring) on activity. Various *N*-aryl sulphonamide derivatives compound **6a–6g** (Table 1) were designed and synthesised with a 3,4,5-trimethoxyphenyl group at the N-1 position (B ring) and a 4-methoxyphenyl group (A ring). Same as we expected, most of the compounds **6a–6g** exhibited moderate to potent antiproliferative activity and were more sensitive to MGC-803 cells. When the C ring of compounds was replaced with electron-donating groups, these compounds exhibited better potency against cancer cells. However, the most active compound **6e** against MGC-803 cell was only at micromolar level (IC_50_ = 2.86 μM) and showed slightly worse potency than compound **1**(IC_50_ = 1.23 μM) and compound **2** (IC_50_ = 1.02 μM). Therefore, a series of compounds **9a–9i** were designed to obtain more effective activity based on compound **6e**.

Next, we investigated the influence of A ring moiety of compounds on activity based on compound **6e**. Compounds **9a–9i** were synthesised with various substituted groups of A ring, a 3,4,5-trimethoxyphenyl group at the N-1 position (B ring), and a 4–4-methoxyphenyl sulphonyl group of C ring (Table 2). Most of the tested compounds displayed moderate to high potency against four cancer cells especially MGC-803 cells.

**Table 2.** *In vitro* antiproliferative activity of compounds **9a–9i** against human cancer cells.

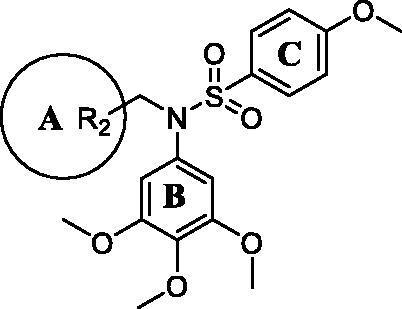					
Compounds	*R*_2_	IC_50_ (μM)^a^			
		MGC-803	HCT-116	PC-3	MCF-7
**9a**	Phenyl	8.57 ± 1.62	18.8 ± 1.72	16.2 ± 1.06	24.5 ± 0.61
**9b**	4-Cl-phenyl	19.2 ± 1.07	23.0 ± 5.25	36.1 ± 0.01	23.4 ± 1.53
**9c**	4-Br-phenyl	31.1 ± 0.79	>50	28.0 ± 2.76	33.8 ± 1.39
**9d**	4-CH_3_-phenyl	5.83 ± 1.75	11.9 ± 2.26	15.6 ± 1.57	41.8 ± 0.43
**9e**	3,4-(OCH_3_)_2_-phenyl	7.92 ± 2.26	8.6 ± 2.85	10.2 ± 0.52	22.9 ± 1.82
**9f**	3-pyridyl	1.31 ± 0.08	6.92 ± 0.79	11.3 ± 1.11	6.31 ± 1.11
**9g**	4-Cl-3-pyridyl	2.62 ± 0.33	24.9 ± 0.58	22.0 ± 1.94	>50
**9h**	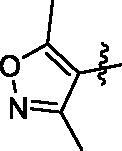	5.48 ± 0.58	12.1 ± 0.85	8.28 ± 3.40	11.5 ± 1.03
**9i**	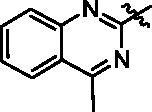	0.36 ± 0.02	0.70 ± 0.04	1.04 ± 0.13	0.81 ± 0.06
**5-Fu**	–	6.82 ± 1.12	14.4 ± 1.73	17.1 ± 1.42	12.1 ± 1.28

^a^*In vitro* antiproliferative activity was assayed by exposure for 48 h.

Compound **9i** with a 4-methy-quinazoline group of (R_2_) displayed most potent *in vitro* antiproliferative activity at nanomolar levels with IC_50_ values of 0.363 μM (MGC-803), 0.708 μM (HCT-116), 1.04 μM (PC-3), and 0.816 μM (MCF-7), respectively. The inhibitory activity of compounds **9a–9i** varied with its substituent groups of A ring. When the R_2_ of A ring was replaced with electron-donating groups, these compounds (**9b** and **9c)** exhibited weaker antiproliferative activity than compounds (**6e**, **9d**, and **9e**) with electron-donating groups of R_2_ at A ring part and compound **9a** with the phenyl group of A ring part. Then, the replacement of phenyl groups of R_2_ with nitrogen-containing heterocycles (**9f**, **9g**, and **9i**) resulted in improved potency against cancer cells than compound **6e**. Similarly, most of the tested compounds exhibited better inhibitory efficacy against MGC-803 cells than other cancer cell lines.

With compound **9i** in hand, we investigated the influence of sulphonyl group part (C ring) on activity. *N*-sulphonamide-quinazoline compounds **10a–10j** were synthesised based on compound **9i** with the quinazoline moiety (A ring). As in Table 3, it was observed that the majority of tested compounds exhibited moderate to high *in vitro* antiproliferative activity. The *in vitro* inhibitory efficacy of compounds **10a–10j** varied with its substituent groups of R_3_ (C ring). Compound **10b**, **10c**, and **10e** displayed potent *in vitro* antiproliferative activity against MGC-803 cells at nanomolar level with IC_50_ values of 0.92, 0.82, and 1.04 μM, respectively. Compounds **9i** and **10e** exhibited better efficacy against MGC-803 cells with electron-donating groups of R_3_ compared to compound **10a**. Compared to compounds **10d**, **10f**, and **10h**, compounds only with the 4-substituent groups of phenyls exhibited potent antiproliferative activity. However, the introduction of heterocyclic substituent groups of R_3_ did not improve efficacy. Importantly, when the R_2_ (A ring) of compounds were replaced with a 4-methy-quinazoline group, these compounds **10a–10j** exhibited improved potency against cancer cells than compounds **6a–6g** with the same substituent groups at the C ring. Similarly, it was more sensitive to MGC-803 cells for the most of tested compounds than other cancer cell lines, including compound **9i**.

**Table 3.** *In vitro* antiproliferative activity of compounds **10a–10j** against human cancer cells.

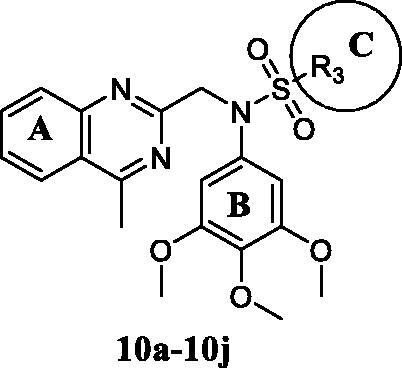					
Compounds	*R*_3_	IC_50_ (μM)^a^			
		MGC-803	HCT-116	PC-3	MCF-7
**10a**	Phenyl	6.62 ± 0.82	16.7 ± 1.03	28.2 ± 2.12	>50
**10b**	4-F-phenyl	0.92 ± 1.07	3.84 ± 0.14	4.72 ± 1.02	5.46 ± 0.87
**10c**	4-Br-phenyl	0.82 ± 0.11	1.49 ± 0.22	1.81 ± 0.26	1.40 ± 0.25
**10d**	2-Cl-phenyl	>50	18.7 ± 1.27	>50	22.1 ± 0.31
**10e**	4-CH_3_-phenyl	1.00 ± 0.08	2.48 ± 0.12	4.01 ± 0.07	1.68 ± 0.12
**10f**	2,4,6-(CH_3_)_3_-phenyl	>50	>50	>50	>50
**10g**	4-C(CH_3_)_3_-phenyl	12.8 ± 1.38	22.4 ± 1.67	18.9 ± 1.57	28.1 ± 1.82
**10h**	3,4-(OCH_3_)_2_-phenyl	14.2 ± 1.03	22.9 ± 1.34	36.6 ± 0.34	18.5 ± 0.81
**10i**	2-thienyl	16.8 ± 1.42	20.4 ± 1.92	27.8 ± 2.02	30.2 ± 1.81
**10j**	2-pyridyl	14.7 ± 1.88	24.8 ± 2.13	28.2 ± 1.98	34.2 ± 1.83
**5-Fu**	–	6.82 ± 1.12	14.4 ± 1.73	17.1 ± 1.42	12.1 ± 1.28

^a^*In vitro* antiproliferative activity was assayed by exposure for 48 h.

Since the 4-methy-quinazoline group of (R_2_) and 4-methoxyphenyl group of (R_3_) are important for maintaining inhibitory, we started to explore the effects of the sulphonamide group on antiproliferative activity. Therefore, *N*-aryl amide quinazoline derivatives **11a–11j** were assessed for their *in vitro* antiproliferative activity (Table 4). However, most *N*-aryl amide quinazoline derivatives exhibited weaker antiproliferative efficacy compared to *N*-aryl sulphonamide quinazoline derivatives. Similarly, compounds **11h** with a 4-methoxyphenyl group of (R_3_) exhibited best inhibitory activity against cancer cells than other substituent groups. Importantly, when the sulphonamide group was replaced with an amide group, the compound **11h** exhibited far below inhibitory potency than compound **9i** with the same substituent groups of A, B, and C ring.

**Table 4.** *In vitro* antiproliferative activities of compounds **11a–11j** against human cancer cells.

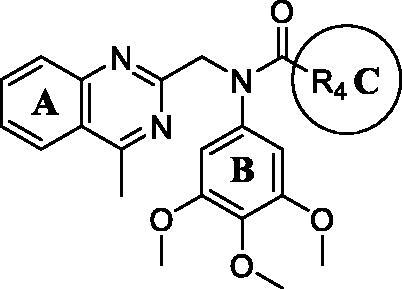					
Compounds	*R*_4_	IC_50_ (μmol/L)^a^			
		MGC-803	HCT-116	PC-3	MCF-7
**11a**	Phenyl	18.5 ± 1.17	19.8 ± 1.49	25.3 ± 1.78	25.3 ± 1.78
**11b**	4-F-phenyl	14.1 ± 1.01	18.9 ± 1.23	31.1 ± 2.04	29.7 ± 1.83
**11c**	4-Br-phenyl	13.6 ± 1.24	20.4 ± 1.23	26.8 ± 1.32	28.1 ± 1.47
**11d**	2-Cl-phenyl	>40	>40	>40	>40
**11e**	4-CH_3_-phenyl	10.8 ± 1.18	21. 1 ± 1.67	24.9 ± 1.41	18.6 ± 1.32
**11f**	2,4,6-(CH_3_)_3_-phenyl	32.8 ± 2.12	28.5 ± 2.64	>40	>40
**11g**	4-C(CH_3_)_3_-phenyl	>40	>40	>40	>40
**11h**	4-OCH_3_-phenyl	5.82 ± 1.17	10.4 ± 1.04	18.7 ± 1.38	22.4 ± 1.54
**11i**	3,4,5-(OCH_3_)_3_-phenyl	16.8 ± 2.02	20.4 ± 1.92	>40	>40
**11j**	2-thienyl	14.7 ± 1.88	24.8 ± 2.13	>40	>40
**5-Fu**	–	6.82 ± 1.12	14.4 ± 1.73	17.1 ± 1.42	12.1 ± 1.28

^a^*In vitro* antiproliferative activity was assayed by exposure for 48 h.

Trimethoxyphenyl (TMP) moiety is essential for many compounds to exhibited high potency against cancer cells^33^^,^^34^. Therefore, we started to investigate the influence of TMP moiety by replacing it with other groups (Table 5). As showed in Table 5, when the 3,4,5-trimethoxyphenyl moiety was replaced by other phenyl moieties, the compounds **14a–14d** exhibited very weak antiproliferative activity against three tested cancer cells with IC_50_ values >50 μM. These results indicated that 3,4,5-trimethoxyphenyl moiety was essential for maintaining inhibitory activity.

**Table 5.** *In vitro* antiproliferative activities of compounds **14a–14d** against human cancer cells.

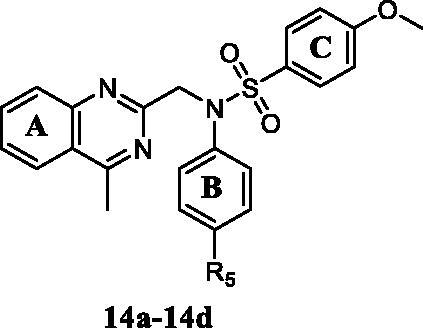					
Comp.	*R*_5_	IC_50_ (μmol/L)^a^			
		MGC-803	HCT-116	PC-3	MCF-7
**14a**	H	44.6 ± 1.41	46.9 ± 1.84	>50	>50
**14b**	Cl	34.7 ± 1.29	40.1 ± 2.51	38.8 ± 2.12	28.6 ± 2.18
**14c**	Br	>50	37.5 ± 0.71	>50	>50
**14d**	CH_3_	42.2 ± 2.44	>50	45.7 ± 2.88	>50
**5-Fu**	–	6.82 ± 1.12	14.4 ± 1.73	17.1 ± 1.42	12.1 ± 1.28

^a^*In vitro* antiproliferative activity was assayed by exposure for 48 h.

Furthermore, MGC-803 cells were more sensitive to the compounds than HCT-116, PC-3 cells MCF-7 cells. Moreover, we chose two other common gastric cancer cell lines (HGC-27 and SGC-7901) and another two normal cells GES-1 and HUEVC cells to examine for the inhibitory activity of compound **9i**. As shown in Table 6, compounds **9i** exhibited high activity against MGC-803, SGC-7901, and HGC-27 cells with IC_50_ values of 0.363, 0.376, and 0.458 μM, respectively. Compounds **9i** exhibited potent activity against GES-1 cells (IC_50_ = 0.894 μM) and moderate activity against HUEVC cells (IC_50_ = 26.19 μM).

**Table 6.** *In vitro* anti-proliferative activity of **9i** against gastric cancer cells (MGC-803, HGC-27, and SGC-7901 cells) and non-cancer cell lines (GES-1 and HUEVC cells).

Compd.	IC_50_ (μM)^a^				
	MGC-803	SGC-7901	HGC-27	GES-1	HUEVC
**9i**	0.363	0.376	0.458	0.894	26.19
**5-Fu**	6.81	3.88	4.94	10.18	18.49

^a^*In vitro* antiproliferative activity was assayed by exposure for 48 h.

**Figure 2.**
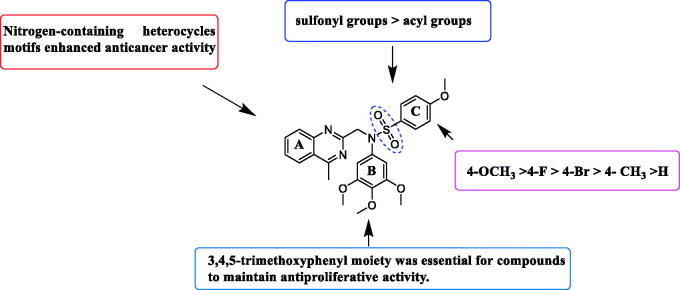
Summary of the structure-activity relationships.

Based on the above preliminary results of *in vitro* antiproliferative activity, the structure-activity relationships were summarised (Figure 2). A 3,4,5-trimethoxyphenyl moiety of B ring was essential for compounds to maintain inhibitory activity against cancer cells. Compounds with nitrogen-containing heterocycle at A ring showed improved activity against cancer cells than other compounds. The electron deficiency or richness of the phenyl group (C ring moiety) might affect *in vitro* antiproliferative activity of compounds partly. Moreover, most *N*-aryl amide quinazoline derivatives exhibited weaker antiproliferative efficacy compared to *N*-aryl sulphonamide quinazoline derivatives.

### 3.2. Compound 9i inhibited gastric cancer cells in vitro

**Figure 3.**
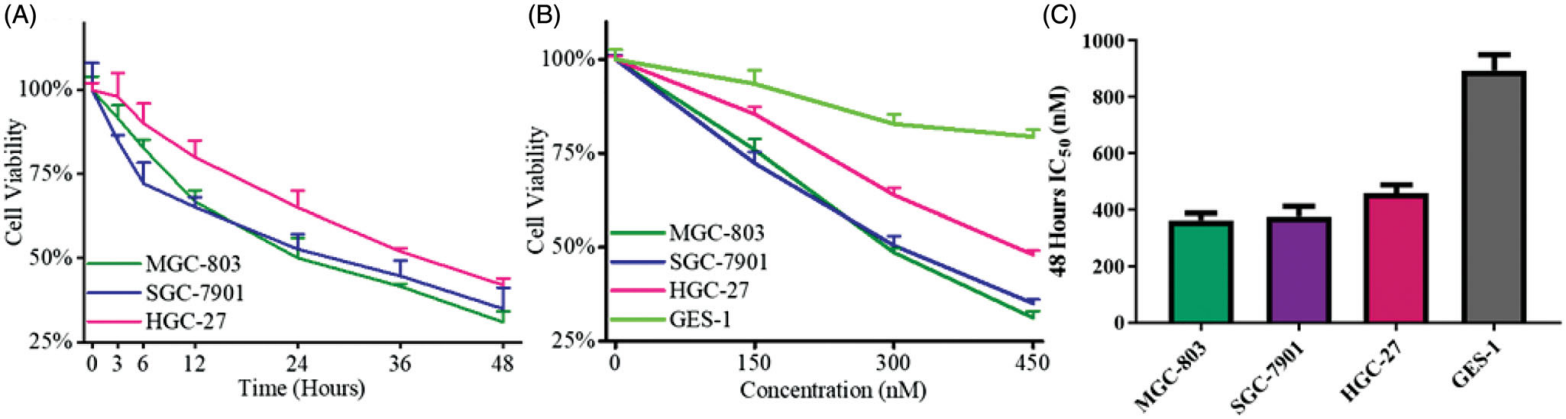
The inhibitory activity of **9i** against gastric cancer cells and normal gastric epithelial cells. (A) Cell viability of three gastric cancer cells after the treatment with 450 nM of **9i** at the different intervals; (B) Cell viability of three gastric cancer cells after the treatment with indicated concentrations of **9i** for 48 h; (C) 48 h IC_50_ values of **9i** against three gastric cancer cells and GES-1 cells.

Since compound **9i** presented high potency against cancer cells and MGC-803 cells was more sensitive to compound **9i**, compound **9i**, MGC-803, another two gastric cancer cells SGC-7901, HGC-27, and GES-1(normal gastric epithelial cells) were used for further detection. As shown in Figures 3(A,B), compound **9i** inhibited three gastric cancer cell lines in the dose and time-dependent manners. The inhibition ratios of compound **9i** against three gastric cancer cells were all above 50% after 48 h in the high dosage group. Moreover, MGC-803 and SGC-7901 cell lines were more sensitive and compound **9i** inhibited over 65% cell viability with high dose treatment for 48 h. Nevertheless, compound **9i** showed low cytotoxicity towards GES-1 cells. The 48 h IC_50_ values of MGC-803, SGC-7901, HGC-27, and GES-1 cells were 0.363, 0.376, 0.458, and 0.894 μM, respectively (Figure 3(C)). It could be summarised from these results that compound **9i** potently inhibited gastric cancer cells in a dose and time-dependent manners.

### 3.3. Compound 9i inhibited activation of YAP in gastric cancer cells

**Figure 4.**
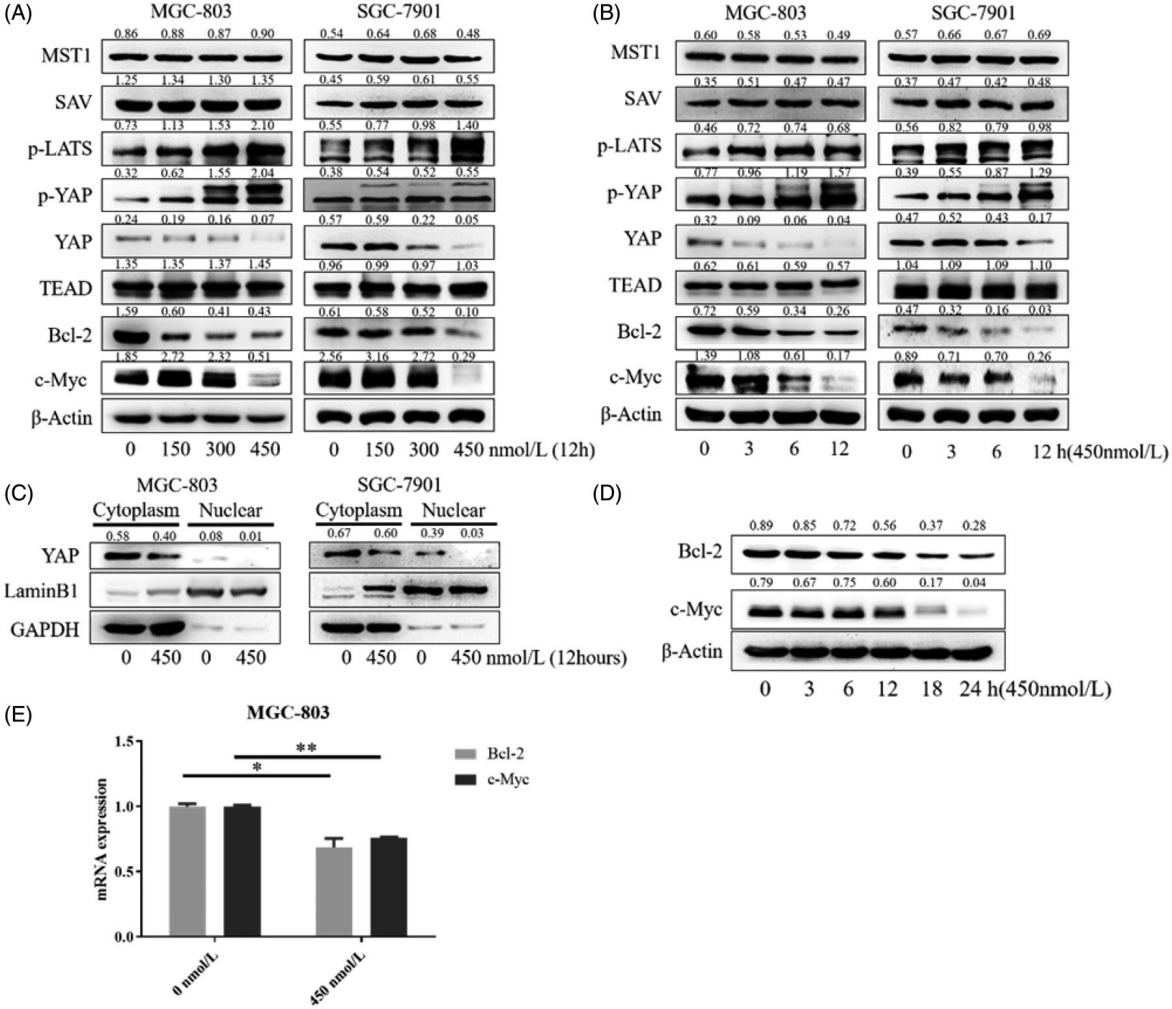
Influence of **9i** on the Hippo signalling pathway in two gastric cancer cells. Density ratios relative to its loading control protein (β-Actin/LaminB1/GAPDH) were shown on the top of each Western blotting band. (A) The levels of Hippo related proteins, cells were treated with indicated concentrations of **9i** for 12 h; (B) The levels of Hippo related proteins in different hours after the treatment with 450 nM of **9i**; (C) Level of YAP in the cytoplasm and nuclear, cells were treated for 12 h with 450 nM of **9i**; (D) Levels of Bcl-2 and c-Myc in MGC-803 cells after the treatment with 450 nM of **9i** for different hours; (E) mRNA levels of Bcl-2 and c-My in MGC-803 cells after the treatment with 450 nM of **9i** 12 h. Data were presented as mean ± *SD*.

Phosphorylation of YAP is the process of YAP inactivation. YAP protein is inactivated (phosphorylated) by Hippo cascade pathway in cells. As shown in Figures 4(A,B), compound **9i** up-regulated the phosphorylation of YAP in MGC-803 and SGC-7901 cells in a dose and time-dependent manner, resulting in a reduced level of YAP. We also investigated the influence of **9i** on upstream signal-regulated molecules of the Hippo pathway. The activation form of LATS, p-LATS was up-regulated, while other upstream proteins including MST1 and SAV did not show an obvious change. Transcription factor TEAD interacts with YAP to express downstream substrates, the level of TEAD was not changed by compound **9i** (Figures 4(A,B)). Yap entering the nucleus is a key step of substrates expression. With the treatment of compound **9i**, the level of YAP inside nuclear was decreased (Figure 4(C)). As the result of the decrease of endonclear activated YAP, Bcl-2, and c-Myc, which are two substrates of YAP-TEAD, were down-regulated at mRNA and protein levels in two gastric cancer cells (Figures 4(A,B,D,E)). These results suggested compound **9i** activated the Hippo signalling pathway from p-LATS then inhibited the activation of YAP and up-regulated the phosphorylation of YAP. As a result, the expression of Bcl-2 and c-Myc were down-regulated at mRNA and protein levels.

### 3.4. Compound 9i induced cell apoptosis in gastric cancer cells

**Figure 5.**
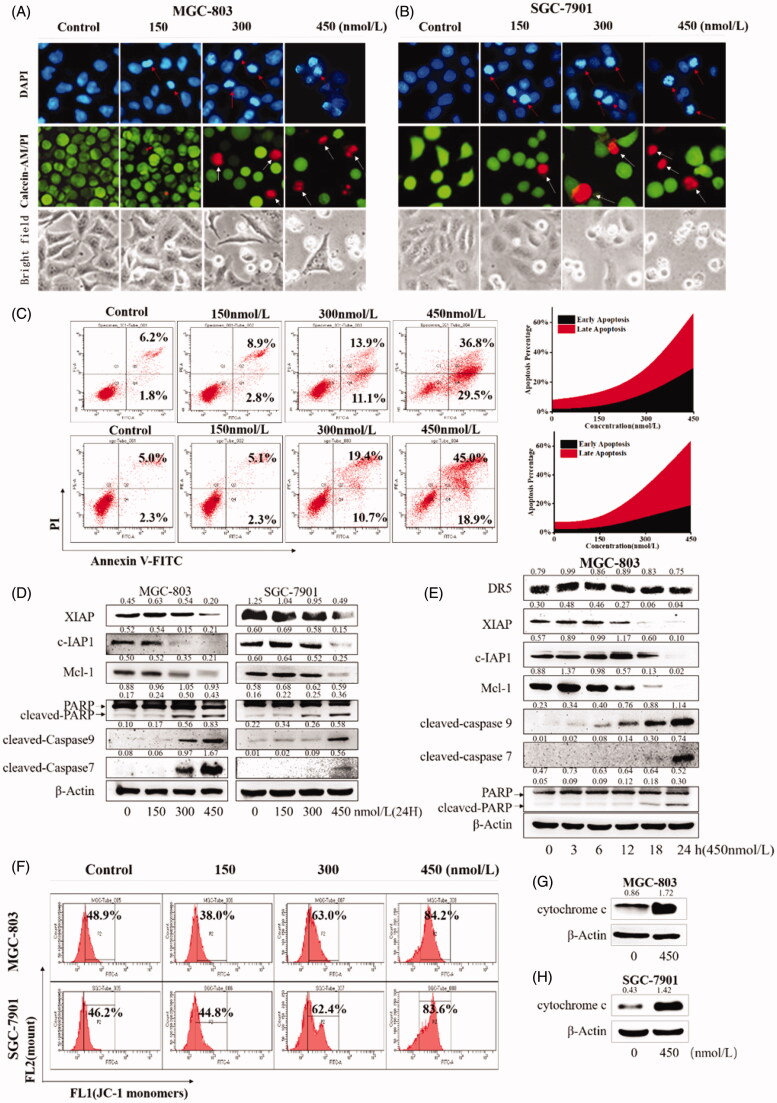
Compound **9i** induced apoptosis in MGC-803 and SGC-7901 cells. Density ratios relative to β-Actin were shown on the top of each Western blotting band. (A,B) Gastric cancer cells’ morphology change, cells were stained with DAPI, Calcein-AM/PI or observed in a bright field after the treatment with indicated concentrations of **9i** for 48 h; (C). Compound **9i** induced MGC-803 and SGC-7901 cells apoptosis at indicated concentrations for 48 h; (D) The levels of apoptosis related proteins in gastric cancer cells after the treatment with indicated concentrations of **9i** for 48 h; (E) The levels of apoptosis related proteins in MGC-803 cancer cells after the treatment with 450 nM of **9i** for different hours; (F) Mitochondrial membrane potential depolarisation induced by indicated concentrations of **9i** in gastric cancer cells for 48 h; (G,H) Level of cytochrome c in the cytoplasm, gastric cancer cells were treated for 48 h with 450 nM of **9i**.

Bcl-2 regulated by the Hippo/YAP plays an important role in cell apoptosis. Therefore, we next examined the effects of compound **9i** on cell apoptosis. MGC-803 and SGC-7901 cells were treated with compound **9i** for 48 h, as shown in Figures 5(A,B), the nuclei of gastric cancer are dense and fragmented, and cells became round, small, and was induced to die. To determine the influence of compound **9i** on cell death, MGC-803 and SGC-7901 cells treated with compound **9i** were stained with Annexin V-FITC/PI. As shown in Figure 5(C), the high dose compound **9i** treatment group induced over 60% of cell apoptosis at 48 h. As shown in Figure 5(D), some other apoptosis-related proteins were also regulated by compound **9i** in MGC-803 and SGC-7901 cells. Compound **9i** caused dose-dependent decreases of anti-apoptosis proteins c-IAP and Mcl-1. The cleavage of Caspase7, Caspase9, and PARP (which were the mark events of apoptosis) was up-regulated in MGC-803 and SGC-7901 cells (Figure 5D). Mitochondrial membrane potential depolarisation and translocation from the mitochondria to the cytoplasm of cytochrome *c* are two key steps involved in the intrinsic apoptosis pathway. As shown in Figures 5(F–H), compound **9i** depolarised mitochondrial membrane potential and induced the translocation of cytochrome c. However, as shown in Figure 5(E), compound **9i** did not make a significant change on the level of the extrinsic apoptosis-related protein DR5 in MGC-803 cells. In summary, these results indicated that compound **9i** induced cell-intrinsic apoptosis in MGC-803 and SGC-7901 cells.

### 3.5. Compound 9i inhibited cell proliferation in gastric cancer cells

**Figure 6.**
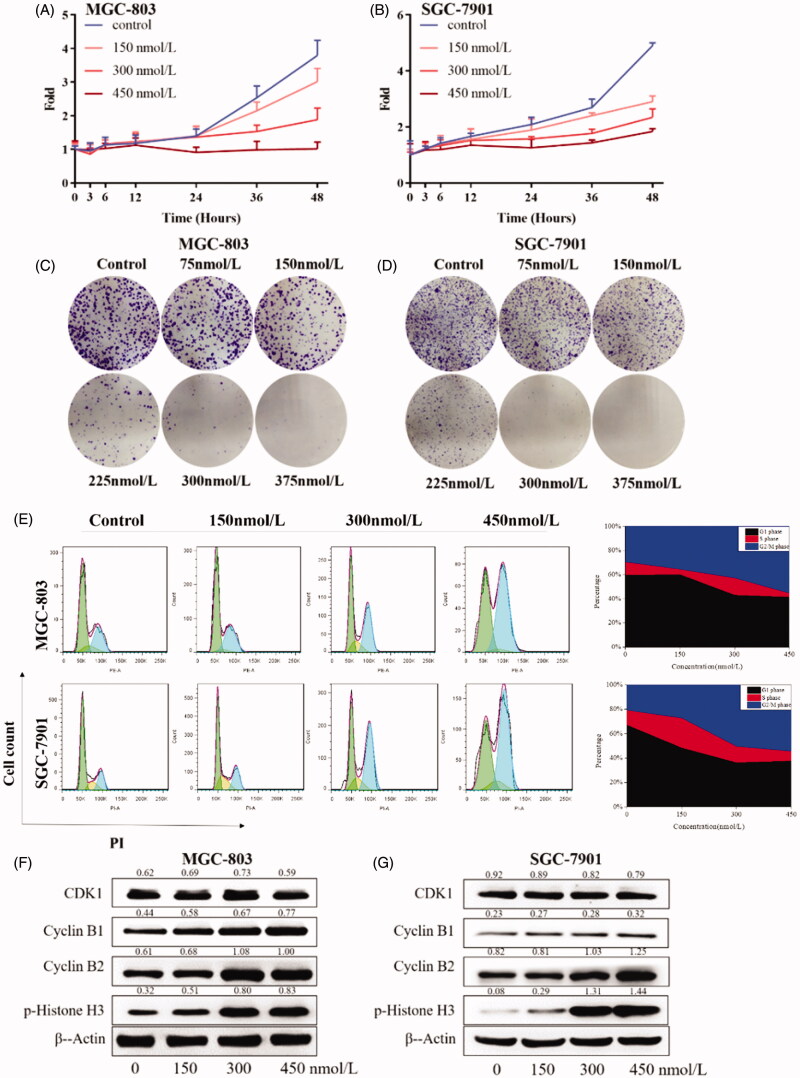
Gastric cancer cell proliferation inhibition inhibited by **9i**. Density ratios relative to β-Actin were shown on the top of each Western blotting band. (A,B) Cell growth curves of in MGC-803 and SGC-7901 cells after the treatment with indicated concentrations of **9i** for different hours; (C,D). Gastric cancer cell colony formation, cells were treated for 7 days; (E) Cell cycle distribution of MGC-803 and SGC-7901 after the treatment with indicated concentrations of **9i** for 24 h; (F,G) Cell cycle related proteins in gastric cancer cells after the treatment with indicated concentrations of **9i** for 24 h.

Because of the potent inhibitory activity on cell viability of compound **9i**, we next explored the effects of compound **9i** on cell proliferation in MGC-803 and SGC-7901 cells. As shown in Figures 6(A,B), compound **9i** inhibited the proliferation of two gastric cancer cells at the concentrations of 150, 300, and 450 nmol/L. Compound **9i** also exhibited potently inhibitory activity of cell colony formation. As shown in Figures 6(C,D), with the treatment of compound **9i**, the formatted colony was significantly decreased at a concentration as low as 150 nM. Cell proliferation inhibition could be caused by cell cycle arrestment, the treatment with compound **9i** for 24 h caused a remarkable and dose-dependent G2/M arrest in MGC-803 and SGC-7901 cells (Figure 6E). At protein levels, compound **9i** did not influence the levels of G2 phase-related protein CDK1, Cyclin B1, and Cyclin B2, but caused dose-dependent increases of the biomarker of M phase protein p-Histone H3 in MGC-803 and SGC-7901 cells (Figures 6(F,G)). These results suggested compound **9i** arrested in MGC-803 and SGC-7901 cells at the G2/M phase to inhibit cell proliferation.

### 3.6. Compound 9i inhibited cancer cells in vivo

**Figure 7.**
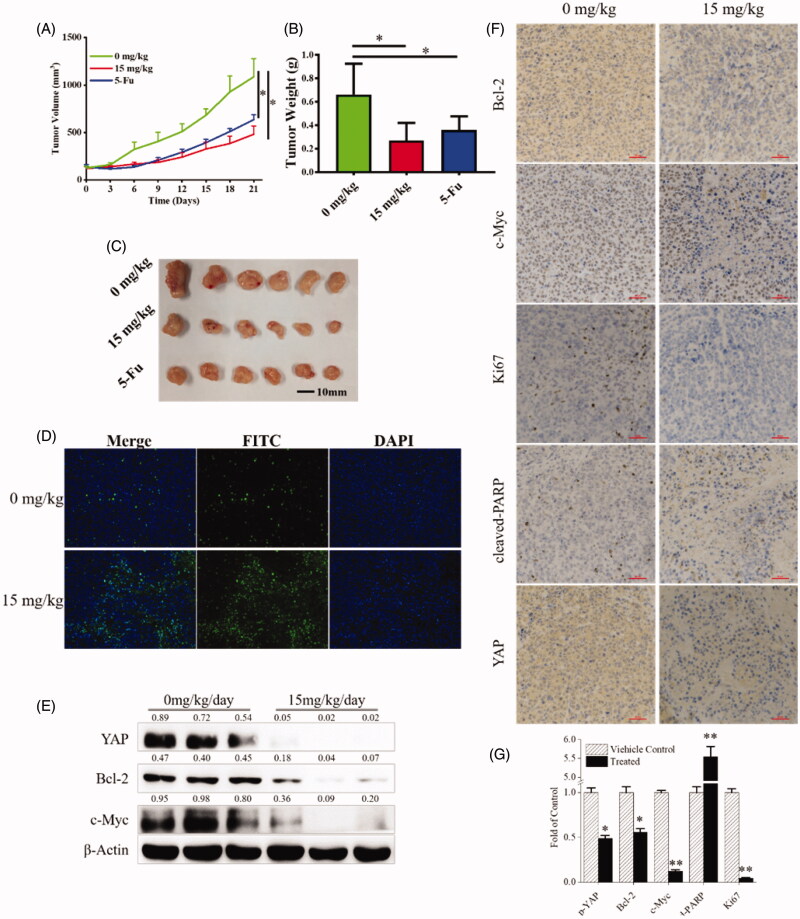
Anti-cancer activity of compound **9i** *in vivo*. (A). Tumour volume during the treatment; (B,C) Tumour weight and pictures, tumour tissues were obtained after mice were executed; (D) Tunnel level of tumour tissues; (E) Expression level of YAP, Bcl-2, c-Myc in tumour tissues. Density ratios relative to β-Actin were shown on the top of each Western blotting band; (F,G) Protein levels of Bcl-2, c-Myc, Ki67, cleaved-PARP and YAP in tumour tissues.

Since compound **9i** effectively inhibited gastric cancer cells *in vitro*, the antitumor efficiency *in vivo* was validated and a xenograft model bearing MGC-803 cells was established. When the tumour volume reached 100 mm^3^, vehicle control, compound **9i**, and **5-FU** were administered by daily intraperitoneal injection for 21 consecutive days. During the treatment, tumour volume and body weight of nude mice were measured every 3 days from the beginning of treatment. As shown in Figure 7(A), compound **9i** inhibited tumour growth *in vivo*. Tumour weight was measured after the execution of animals, the inhibition rate of the compound **9i** treated group was over 60% than the vehicle group (Figures 7(B,C)). Tunnel staining was used for cell apoptosis detection, as shown in Figure 7(D), compound **9i** increased the level of cell apoptosis. We also detected the levels of three Hippo/YAP related proteins including YAP, Bcl-2, and c-Myc in tissues, compound **9i** significantly down-regulated the expression of YAP, Bcl-2, and c-Myc, which was consistent with them at cell levels (Figure 7E). Next, the levels of YAP, Bcl-2, c-Myc, Ki67, and cleaved PARP in tissues were detected using immunochemistry to validate the results. As shown in Figures 7(F,G), compound **9i** downregulated the expression of YAP and its substrates Bcl-2, c-Myc, cell proliferation biomarker Ki67, while up-regulated the expression of YAP cell apoptosis biomarker cleaved-PARP, which was consistent with them at cell levels. The data indicated compound **9i** inhibited YAP to inhibit the tumour *in vivo*.

### 3.7. Compound 9i shows no significant toxicity in vivo

**Figure 8.**
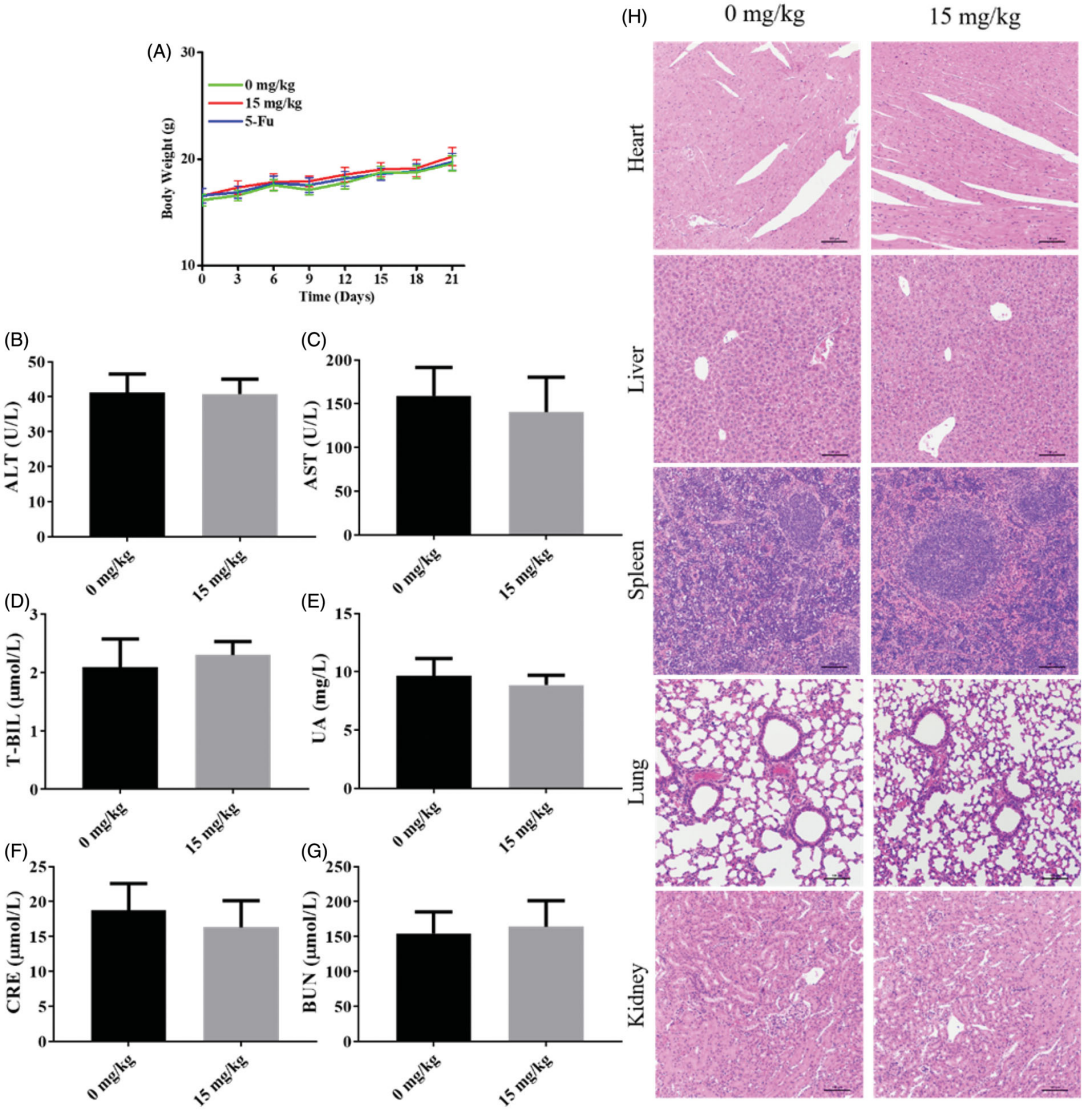
The safety of compound **9i** *in vivo*. (A) Body weight of mice during the treatment; (B–G) Blood biochemical indexes of blood samples; (H) Representative H&E staining of different organs.

The bodyweight of mice was recorded during the treatment every 3 days. The bodyweight of the drug-treated group did not show a significant difference from the vehicle control group (Figure 8A). Blood samples and organs were collected after mice were executed. There is no significant difference in blood biochemical indexes of alanine aminotransferase (ALT), glutamic oxalacetic transaminase (AST), total bilirubin (T-BIL), uric acid (UA), creatinine (CRE), blood urea nitrogen (BUN) between the drug-treated group and the vehicle control group, these results indicated that compound **9i** has low toxicity to functions of liver or kidney (Figures 8(B–G)). H&E staining was performed on organ samples, as shown in Figure 8(H), there was no obvious damage was observed in cell morphology (Figure 8(H)). These results above indicated that compound **9i** did not show obvious toxicity *in vivo.*

## 4. Conclusion

In this study, we reported novel *N*-aryl sulphonamide derivatives and their antiproliferative activity against four human cancer cell lines (MGC-803, HCT-116 cells, PC-3, and MCF-7). Compound **9i** with a 4-methy-quinazoline group showed high activity against cancer cells with IC_50_ values at nanomolar levels, especially MGC-803 and SGC-7901 cells. Compound **9i** inhibited MGC-803 and SGC-7901 cells in time and dose-dependent manners and exhibited low toxicity on GES-1 cells (Figure 3). Compound **9i** activated the Hippo signalling pathway from p-LATS then inhibited the activation of YAP and up-regulated the phosphorylation of YAP. As a result, the expression of Bcl-2 and c-Myc were down-regulated at mRNA and protein levels (Figure 4). Hippo pathway could regulate cell behaviours, such as cell apoptosis and proliferation. As the results indicated, compound **9i** induced cell apoptosis (Figure 5) and inhibited cell proliferation in MGC-803 and SGC-7901 cells (Figure 6). The antitumor efficiency *in vivo* was validated and a xenograft model bearing MGC-803 cells was established. Compound **9i** was effective in suppressing MGC-803 xenograft tumour growth in nude mice (Figure 7) and showed low toxicity to mice (Figure 8). Importantly, Compound **9i** also significantly down-regulated YAP *in vivo* (Figure 7).

**Figure 9.**
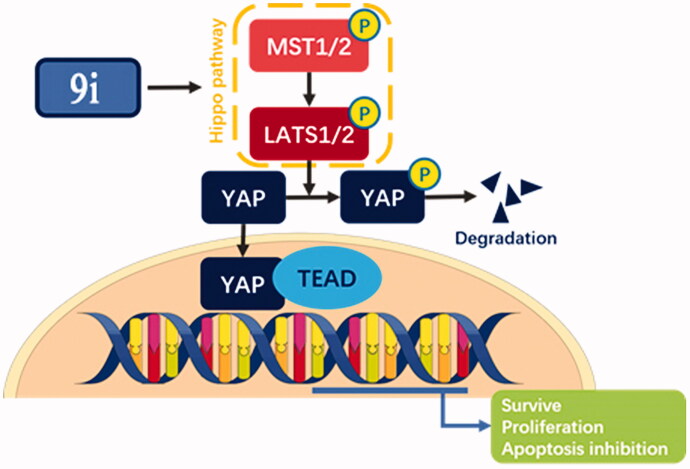
Model of a mechanism by which compound **9i** activated Hippo pathway to inhibit gastric cancer.

In summary, compound **9i** is an efficient compound that activated the Hippo pathway to inhibit YAP which led to the cell apoptosis and proliferation inhibition of gastric cancer cells *in vitro* and *in vivo* (Figure 9).

## 5. General information

All the chemical reagents were purchased from commercial suppliers (Energy chemical Company and Zhengzhou He Qi Company). Melting points were determined on an X-5 micro melting apparatus. NMR spectra data was recorded with a Bruker spectrometer. HRMS spectra data were obtained using a Waters Micromass spectrometer.

### 5.1. Synthesis of compound 5 and 8a–8i

To a solution of substituted anilines, **3** and **12a–12d** (1.0 mmol) in DMF (20 ml) was added K_2_CO_3_ (2.0 mmol) and substituted chloromethyl intermediates **4** and **7a–7i** (1.0 mmol). The reaction mixtures were stirred at 25 °C for 6 h. Then, the resulting solution was poured into ice water and extracted with ethyl acetate three times. And then, using saturated saltwater washed collected organic layers and magnesium sulphate anhydrous dried over organic layers. Organic layers were evaporated to give crude products. Crude products were purified by column chromatography (PE:EA = 2:1) to give intermediates **5**, **8a–8i**, and **13a–13d**.

### 5.2. Synthesis of compounds 6a–6g, 9a–9i, 10a–10j, 11a–11j, and 14a–14d

To a solution of intermediates **5**, **8a–8i**, and **13a–13d** (1.0 mmol) in DCM (20 ml) was added triethylamine (1.5 mmol) and sulphonyl chloride derivatives or acyl chloride derivatives (1.2 mmol). The reaction mixtures were stirred at 25 °C for 4 h. Then, organic layers were evaporated to give crude products. Crude products were purified by column chromatography (PE:EA = 2:1) to give compounds **6a–6g**, **9a–9i**, **10a–10j**, **11a–11j**, and **14a–14d**.

#### 5.2.1. 4-Fluoro-N-(4-methoxybenzyl)-N-(3,4,5-trimethoxyphenyl) benzene-sulphonamide (6a)

White powder, Yield, 62%, m.p. 156–157 °C.^1^H NMR (400 MHz, DMSO-*d_6_*) δ 7.78 (dd, *J* = 8.7, 5.2 Hz, 2H), 7.48 (t, *J* = 8.8 Hz, 2H), 7.18 (d, *J* = 8.6 Hz, 2H), 6.83 (d, *J* = 8.6 Hz, 2H), 6.25 (s, 2H), 4.68 (s, 2H), 3.69 (s, 3H), 3.59 (d, *J* = 10.5 Hz, 9H). ^13^C NMR (100 MHz, DMSO-*d_6_*) δ 165.86, 163.36, 158.56, 152.40, 136.98, 134.20, 134.17, 134.05, 130.69, 130.59, 129.60, 127.90, 116.52, 116.29, 113.66, 106.50, 59.97, 55.88, 54.94, 53.22. HR-MS (ESI): Calcd, C_23_H_24_FNO_6_S, [M + Na]^+^: 484.1201, found: 484.1196.

#### 5.2.2. 4-Chloro-N-(4-methoxybenzyl)-N-(3,4,5-trimethoxyphenyl) benzene-sulphonamide (6b)

White powder, Yield, 58%, m.p. 166–167 °C. ^1^H NMR (400 MHz, DMSO-*d_6_*) δ 7.71 (s, 4H), 7.17 (d, *J* = 8.5 Hz, 2H), 6.82 (d, *J* = 8.6 Hz, 2H), 6.26 (s, 2H), 4.68 (s, 2H), 3.69 (s, 3H), 3.59 (d, *J* = 9.5 Hz, 9H). ^13^C NMR (101 MHz, DMSO-*d_6_*) δ 158.57, 152.40, 138.11, 137.00, 136.62, 133.92, 129.61, 129.48, 129.37, 127.81, 113.67, 106.51, 59.98, 55.88, 54.94, 53.21. HR-MS (ESI): Calcd, C_23_H_24_ClNO_6_S, [M + Na]^+^: 500.0905, found: 500.0902.

#### 5.2.3. 4-Bromo-N-(4-methoxybenzyl)-N-(3,4,5-trimethoxyphenyl) benzene-sulphonamide (6c)

White powder, Yield, 66%, m.p. 153–154 °C. ^1^H NMR (400 MHz, DMSO-*d_6_*) δ 7.86 (d, *J* = 8.6 Hz, 2H), 7.63 (d, *J* = 8.6 Hz, 2H), 7.17 (d, *J* = 8.6 Hz, 2H), 6.82 (d, *J* = 8.7 Hz, 2H), 6.25 (s, 2H), 4.67 (s, 2H), 3.69 (s, 3H), 3.59 (d, *J* = 9.3 Hz, 9H). ^13^C NMR (101 MHz, DMSO-*d_6_*) δ 158.58, 152.40, 137.03, 133.90, 132.32, 129.61, 129.55, 127.81, 127.11, 113.67, 106.52, 59.98, 55.89, 54.94, 53.19. HR-MS (ESI): Calcd, C_23_H_24_BrNO_6_S, [M + Na]^+^: 544.0400, found: 544.0396.

#### 5.2.4. 4-Methoxy-N-(4-methoxybenzyl)-N-(3,4,5-trimethoxyphenyl) benzene-sulphonamide (6e)

White powder, Yield, 48%, m.p. 179–180 °C. ^1^H NMR (400 MHz, DMSO-*d_6_*) δ 7.64 (d, *J* = 8.8 Hz, 2H), 7.16 (t, *J* = 9.5 Hz, 4H), 6.82 (d, *J* = 8.5 Hz, 2H), 6.23 (s, 2H), 4.63 (s, 2H), 3.86 (s, 3H), 3.68 (s, 3H), 3.59 (d, *J* = 11.3 Hz, 9H).^13^C NMR (101 MHz, DMSO-*d_6_*) δ 162.73, 158.50, 152.32, 136.82, 134.45, 129.78, 129.55, 129.44, 128.13, 114.34, 113.62, 106.44, 59.97, 55.84, 55.73, 54.93, 53.09. HR-MS (ESI): Calcd, C_24_H_27_NO_7_S, [M + Na]^+^: 496.1400, found: 496.1397.

#### 5.2.5. N-(4-methoxybenzyl)-N-(3,4,5-trimethoxyphenyl) thiophene-2-sulphonamide (6f)

White powder, Yield, 56%, m.p. 181–182 °C. ^1^H NMR (400 MHz, DMSO-*d_6_*) δ 8.06 (dd, *J* = 5.0, 1.1 Hz, 1H), 7.66 (dd, *J* = 3.7, 1.1 Hz, 1H), 7.32–7.14 (m, 3H), 6.83 (d, *J* = 8.6 Hz, 2H), 6.27 (s, 2H), 4.70 (s, 2H), 3.69 (s, 3H), 3.60 (d, *J* = 6.9 Hz, 9H). ^13^C NMR (101 MHz, DMSO-*d_6_*) δ 158.60, 152.41, 137.81, 137.07, 133.97, 133.81, 133.22, 129.63, 128.01, 127.80, 113.68, 106.39, 59.97, 55.86, 54.95, 53.43. HR-MS (ESI): Calcd, C_21_H_23_NO_6_S_2_, [M + Na]^+^: 472.0859, found: 472.0856.

#### 5.2.6. N-(4-methoxybenzyl)-N-(3,4,5-trimethoxyphenyl) pyridine-3-sulphonamide (6g)

White powder, Yield, 48%, m.p. 162–163 °C.^1^H NMR (400 MHz, DMSO-*d_6_*) δ 8.95–8.81 (m, 2H), 8.15–8.03 (m, 1H), 7.68 (dd, *J* = 7.9, 4.8 Hz, 1H), 7.19 (d, *J* = 8.6 Hz, 2H), 6.83 (d, *J* = 8.6 Hz, 2H), 6.27 (s, 2H), 4.74 (s, 2H), 3.69 (s, 3H), 3.61 (s, 3H), 3.57 (s, 6H). ^13^C NMR (101 MHz, DMSO-*d_6_*) δ 158.61, 153.62, 152.49, 147.68, 137.11, 135.56, 134.26, 133.67, 129.65, 127.73, 124.31, 113.70, 106.57, 59.99, 55.90, 54.95, 53.31.HR-MS (ESI): Calcd, C_22_H_24_N_2_O_6_S, [M + H]^+^: 445.1428, found: 445.1424.

#### 5.2.7. N-benzyl-4-methoxy-N-(3,4,5-trimethoxyphenyl) benzene-sulfonamide (9a)

White powder, Yield, 64%, m.p. 161–162 °C. ^1^H NMR (400 MHz, DMSO-*d_6_*) δ 7.69–7.61 (m, 2H), 7.31–7.25 (m, 4H), 7.19–7.12 (m, 2H), 6.26 (s, 2H), 4.72 (s, 2H), 3.87 (s, 3H), 3.60 (s, 3H), 3.57 (s, 6H). ^13^C NMR (101 MHz, DMSO-*d_6_*) δ 162.78, 152.34, 136.87, 136.47, 134.54, 129.80, 129.39, 128.24, 128.14, 127.34, 114.36, 106.42, 59.97, 55.84, 55.75, 53.70. HR-MS (ESI): Calcd, C_23_H_25_NO_6_S, [M + Na]^+^: 466.1295, found: 466.1297.

#### 5.2.8. N-(4-chlorobenzyl)-4-methoxy-N-(3,4,5-trimethoxyphenyl) benzene-sulphonamide (9b)

White powder, Yield, 59%, m.p. 172–173 °C.^1^H NMR (400 MHz, DMSO-*d_6_*) δ 7.67–7.59 (m, 2H), 7.33 (d, *J* = 2.2 Hz, 4H), 7.22–7.12 (m, 2H), 6.27 (s, 2H), 4.71 (s, 2H), 3.86 (s, 3H), 3.60 (d, *J* = 6.7 Hz, 9H). ^13^C NMR (101 MHz, DMSO-*d_6_*) δ 162.83, 152.40, 136.93, 135.60, 134.40, 131.93, 129.99, 129.83, 129.16, 128.26, 114.38, 106.38, 59.98, 55.87, 55.75, 52.93. HR-MS (ESI): Calcd, C_23_H_24_ClNO_6_S, [M + Na]^+^: 500.0905, found: 500.0911.

#### 5.2.9. N-(4-bromobenzyl)-4-methoxy-N-(3,4,5-trimethoxyphenyl) benzene-sulphonamide (9c)

White powder, Yield, 38%, m.p. 187–188 °C. ^1^H NMR (400 MHz, DMSO-*d_6_*) δ 7.64 (d, *J* = 8.9 Hz, 2H), 7.48 (d, *J* = 8.4 Hz, 2H), 7.26 (d, *J* = 8.4 Hz, 2H), 7.16 (d, *J* = 8.9 Hz, 2H), 6.28 (s, 2H), 4.70 (s, 2H), 3.86 (s, 3H), 3.60 (d, *J* = 6.5 Hz, 9H). ^13^C NMR (101 MHz, DMSO-*d_6_*) δ 162.83, 152.39, 136.91, 136.03, 134.40, 131.18, 130.34, 129.83, 129.12, 120.47, 114.38, 106.35, 59.97, 55.87, 55.74, 52.97. HR-MS (ESI): Calcd, C_23_H_24_BrNO_6_S, [M + Na]^+^: 544.0400, found: 544.0397.

#### 5.2.10. 4-Methoxy-N-(4-methylbenzyl)-N-(3,4,5-trimethoxyphenyl) benzene-sulphonamide (9d)

White powder, Yield, 52%, m.p. 164–165 °C. ^1^H NMR (400 MHz, DMSO-*d_6_*) δ 7.63 (d, *J* = 8.8 Hz, 2H), 7.19–7.03 (m, 6H), 6.25 (s, 2H), 4.66 (s, 2H), 3.86 (s, 3H), 3.58 (d, *J* = 10.2 Hz, 9H). ^13^C NMR (101 MHz, DMSO-*d_6_*) δ 162.75, 152.31, 136.80, 136.47, 134.50, 133.32, 129.78, 129.35, 128.82, 128.13, 114.34, 106.37, 59.96, 55.83, 55.73, 53.36, 20.61. HR-MS (ESI): Calcd, C_24_H_27_NO_6_S, [M + H]^+^: 458.1632, found: 458.1625.

#### 5.2.11. N-(3,4-dimethoxybenzyl)-4-methoxy-N-(3,4,5-trimethoxyphenyl) benzene-sulphonamide (9e)

Light yellow powder, Yield, 49%, m.p. 136–137 °C. ^1^H NMR (400 MHz, DMSO-*d_6_*) δ 7.64 (d, *J* = 8.9 Hz, 2H), 7.16 (d, *J* = 8.9 Hz, 2H), 6.84–6.71 (m, 3H), 6.26 (s, 2H), 4.63 (s, 2H), 3.86 (s, 3H), 3.67 (d, *J* = 5.4 Hz, 6H), 3.59 (d, *J* = 7.7 Hz, 9H). ^13^C NMR (101 MHz, DMSO-*d_6_*) δ 162.76, 152.30, 148.41, 148.02, 136.81, 134.46, 129.82, 129.35, 128.39, 120.64, 114.34, 111.75, 111.35, 106.47, 59.97, 55.85, 55.74, 55.32, 53.32. HR-MS (ESI): Calcd, C_25_H_29_NO_8_S, [M + Na]^+^: 526.1506, found: 526.1514.

#### 5.2.12. 4-Methoxy-N-(pyridin-3-ylmethyl)-N-(3,4,5-trimethoxyphenyl) benzene-sulphonamide (9f)

White powder, Yield, 55%, m.p. 158–159 °C. ^1^H NMR (400 MHz, DMSO) δ 8.42 (dd, *J* = 6.4, 1.6 Hz, 2H), 7.77–7.60 (m, 3H), 7.32 (dd, *J* = 7.7, 4.8 Hz, 1H), 7.17 (d, *J* = 8.9 Hz, 2H), 6.27 (s, 2H), 4.76 (s, 2H), 3.87 (s, 3H), 3.59 (d, *J* = 6.7 Hz, 9H).^13^C NMR (101 MHz, DMSO) δ 162.88, 152.44, 149.37, 148.64, 136.97, 135.94, 134.24, 132.10, 129.91, 128.98, 123.45, 114.40, 106.41, 59.98, 55.88, 55.76, 51.22. HR-MS (ESI): Calcd, C_22_H_24_N_2_O_6_S, [M + H]^+^: 445.1428, found: 445.1423.

#### 5.2.13. N-((6-chloropyridin-3-yl)methyl)-4-methoxy-N-(3,4,5-trimethoxyphenyl) benzene-sulfonamide (9g)

White powder, Yield, 49%, m.p. 160–161 °C. ^1^H NMR (400 MHz, DMSO) δ 8.27 (d, *J* = 2.3 Hz, 1H), 7.80 (dd, *J* = 8.3, 2.5 Hz, 1H), 7.67–7.63 (m, 2H), 7.45 (d, *J* = 8.2 Hz, 1H), 7.22–7.12 (m, 2H), 6.28 (s, 2H), 4.76 (s, 2H), 3.87 (s, 3H), 3.61 (d, *J* = 4.8 Hz, 9H).^13^C NMR (101 MHz, DMSO) δ 162.94, 152.48, 149.48, 149.26, 139.65, 137.03, 134.12, 131.90, 129.94, 128.71, 124.06, 114.42, 106.38, 59.99, 55.91, 55.77, 50.39. HR-MS (ESI): Calcd, C_22_H_23_ClN_2_O_6_S, [M + H]^+^: 479.1038, found: 479.1039.

#### 5.2.14. N-((3,5-dimethylisoxazol-4-yl)methyl)-4-methoxy-N-(3,4,5-trimethoxyphenyl) benzene-sulfonamide (9h)

White powder, Yield, 51%, m.p. 125–126 °C.^1^H NMR (400 MHz, DMSO-*d_6_*) δ 7.65 (d, *J* = 8.9 Hz, 2H), 7.17 (d, *J* = 8.9 Hz, 2H), 6.21 (s, 2H), 4.52 (s, 2H), 3.86 (s, 3H), 3.61 (d, *J* = 15.5 Hz, 9H), 2.10 (s, 3H), 2.04 (s, 3H). ^13^C NMR (101 MHz, DMSO-*d_6_*) δ 167.43, 162.83, 159.40, 152.52, 137.36, 134.02, 129.90, 129.14, 114.41, 109.09, 106.68, 60.11, 55.94, 55.74, 10.14, 9.46. HR-MS (ESI): Calcd, C_22_H_26_N_2_O_7_S, [M + H]^+^: 463.1533, found: 463.1533.

#### 5.2.15. 4-Methoxy-N-((4-methylquinazolin-2-yl)methyl)-N-(3,4,5-trimethoxyphenyl) benzene-sulfonamide (9i)

Light yellow powder, Yield, 62%, m.p. 176–177 °C. ^1^H NMR (400 MHz, DMSO-*d_6_*) δ 8.17–8.12 (m,1H), 7.88 (ddd, *J* = 8.3, 6.9, 1.3 Hz, 1H), 7.77 (d, *J* = 8.0 Hz, 1H), 7.64–7.58 (m,3H), 7.03–6.98 (m,2H), 6.57 (s,2H), 5.04 (s,2H), 3.75 (s,3H), 3.53 (d, *J* = 7.5 Hz, 9H), 2.76 (s,3H).^13^C NMR (100 MHz, DMSO-*d_6_*) δ 168.76, 162.57, 160.79, 152.28, 148.90, 136.83, 135.78, 134.22, 130.92, 129.85, 127.79, 127.54, 125.68, 122.33, 114.00, 106.91, 59.93, 57.11, 55.71, 21.36. HR-MS (ESI) calcd, C_26_H_27_N_3_O_6_S [M + Na]^+^:532.1518, found: 532.1517.

#### 5.2.16. N-((4-methylquinazolin-2-yl)methyl)-N-(3,4,5-trimethoxyphenyl) benzene-sulphonamide (10a)

Light yellow powder, Yield, 66%, m.p. 178–179 °C. ^1^H NMR (400 MHz, DMSO-*d_6_*) δ 8.23 (d, *J* = 7.7 Hz, 1H), 7.96 (ddd, *J* = 8.3, 6.9, 1.3 Hz, 1H), 7.82 (d, *J* = 8.1 Hz, 1H), 7.78 (s,1H), 7.76 (d, *J* = 1.3 Hz, 1H), 7.70 (t, *J* = 7.4 Hz, 2H), 7.58 (t, *J* = 7.7 Hz, 2H), 6.61 (s,2H), 5.16 (s,2H), 3.59 (d, *J* = 1.6 Hz, 9H), 2.83 (s,3H).^13^C NMR (101 MHz, DMSO-*d_6_*) δ 168.82, 160.72, 152.29, 148.87, 139.32, 136.94, 135.41, 134.27, 132.92, 128.92, 127.76, 127.64, 127.57, 125.72, 122.34, 107.00, 59.93, 57.22, 55.67, 30.66, 21.37. HR-MS (ESI): Calcd, C_25_H_25_N_3_O_5_S[M + H]^+^:480.1593, found: 480.1592.

#### 5.2.17. 4-Fluoro-N-((4-methylquinazolin-2-yl)methyl)-N-(3,4,5-trimethoxyphenyl) benzene-sulfonamide (10b)

White powder, Yield, 64%, m.p. 162–163 °C.^1^H NMR (400 MHz, DMSO-*d_6_*) δ 8.22 (d, *J* = 8.2 Hz, 1H), 7.96 (t, *J* = 7.6 Hz, 1H), 7.89–7.79 (m, 3H), 7.70 (t, *J* = 7.5 Hz, 1H), 7.42 (t, *J* = 8.6 Hz, 2H), 6.66 (s,2H), 5.17 (s,2H), 3.60 (d, *J* = 7.2 Hz, 9H), 2.83 (s,3H). ^13^C NMR (101 MHz, DMSO-*d_6_*) δ 168.84, 165.70, 163.20, 160.61, 152.37, 148.84, 137.02, 135.85, 135.35, 134.29, 130.79, 130.69, 127.70, 127.58, 125.72, 122.32, 116.14, 115.91, 107.03, 59.93, 57.19, 55.72, 21.35. HR-MS (ESI): Calcd, C_25_H_24_FN_3_O_5_S [M + H]^+^:498.1499, found: 498.1498

#### 5.2.18. 4-Bromo-N-((4-methylquinazolin-2-yl)methyl)-N-(3,4,5-trimethoxyphenyl) benzene-sulfonamide (10c)

White powder, Yield, 58%, m.p. 170–171 °C. ^1^H NMR (400 MHz, DMSO-*d_6_*) δ 8.23 (d, *J* = 8.3 Hz, 1H), 7.96 (ddd, *J* = 8.3, 7.0, 1.3 Hz, 1H), 7.81–7.75 (m,3H), 7.71 (dd, *J* = 9.5, 2.3 Hz, 1H), 7.69–7.65 (m,2H), 6.71 (s,2H), 5.17 (s,2H), 3.62 (d, *J* = 10.1 Hz, 9H), 2.82 (s,3H).^13^C NMR (101 MHz, DMSO-*d_6_*) δ 168.82, 160.49, 152.39, 148.80, 138.83, 137.07, 135.36, 134.29, 131.94, 129.61, 127.70, 127.61, 126.80, 125.70, 122.31, 107.08, 59.94, 57.25, 55.73, 21.32. HR-MS (ESI): Calcd, C_25_H_24_BrN_3_O_5_S [M + H]^+^:558.0698, found:558.0699.

#### 5.2.19. 2-Chloro-N-((4-methylquinazolin-2-yl)methyl)-N-(3,4,5-trimethoxyphenyl) benzene-sulfonamide (10d)

Light yellow powder, Yield, 50%, m.p. 183–184 °C.^1^H NMR (400 MHz, DMSO-*d_6_*) δ 8.18 (d, *J* = 6.5 Hz, 1H), 7.97–7.91 (m,2H), 7.84 (d, *J* = 8.2 Hz, 1H), 7.73–7.66 (m,2H), 7.65–7.61 (m,1H), 7.50–7.40 (m,1H), 6.76 (s,2H), 5.36 (s,2H), 3.57 (d, *J* = 10.1 Hz, 9H), 2.85 (s,3H). ^13^C NMR (101 MHz, DMSO-*d_6_*) δ 168.82, 160.97, 153.28, 152.34, 148.87, 136.99, 134.78, 134.23, 132.18, 131.88, 129.83, 127.75, 127.49, 125.67, 106.96, 59.91, 57.87, 55.66, 21.39. HR-MS (ESI): Calcd, C_25_H_24_ClN_3_O_5_S [M + H]^+^:514.1203, found:514.1202.

#### 5.2.20. 4-Methyl-N-((4-methylquinazolin-2-yl)methyl)-N-(3,4,5-trimethoxyphenyl) benzene-sulfonamide (10e)

White powder, Yield, 65%, m.p. 182–183 °C. ^1^H NMR (400 MHz, DMSO-*d_6_*) δ 8.25 (t, *J* = 8.0 Hz, 1H), 8.00–7.94 (m, 1H), 7.83 (d, *J* = 8.2 Hz, 1H), 7.71 (t, *J* = 7.6 Hz, 1H), 7.64 (d, *J* = 8.2 Hz, 2H), 7.36 (d, *J* = 8.1 Hz, 2H), 6.64 (s,2H), 5.13 (s,2H), 3.62 (t, *J* = 14.6 Hz, 9H), 2.84 (s,3H), 2.39 (s,3H).^13^C NMR (101 MHz, DMSO-*d_6_*) δ 168.78, 160.73, 152.27, 148.88, 143.29, 136.86, 136.48, 135.65, 134.24, 129.31, 127.78, 127.64, 127.56, 125.69, 122.33, 106.94, 59.94, 57.15, 55.69, 21.34, 20.96. HRMS (ESI) calcd for C_26_H_27_N_3_O_5_S [M + H]^+^:494.1750, found:494.1749.

#### 5.2.21. 2,4,6-Trimethyl-N-((4-methylquinazolin-2-yl)methyl)-N-(3,4,5-trimethoxyphenyl) benzene-sulfonamide (10f)

White powder, Yield, 49%, m.p. 154–155 °C. ^1^H NMR (400 MHz, DMSO-*d_6_*) δ 8.12 (d, *J* = 8.3 Hz, 1H), 7.87 (t, *J* = 7.6 Hz, 1H), 7.77 (d, *J* = 8.4 Hz, 1H), 7.60 (t, *J* = 7.6 Hz, 1H), 6.83 (s,2H), 6.62 (s,2H), 5.10 (s,2H), 3.50 (s,9H), 2.77 (s,3H), 2.38 (s,6H), 2.08 (s,3H).^13^C NMR (101 MHz, DMSO-*d_6_*) δ 168.62, 160.78, 152.26, 148.84, 142.35, 139.92, 136.94, 135.38, 134.17, 132.88, 131.36, 127.78, 127.50, 125.61, 122.24, 107.27, 60.00, 56.24, 55.66, 22.55, 21.36, 20.26. HR-MS (ESI): Calcd, C_28_H_31_N_3_O_5_S[M + H]^+^:522.2063, found:522.2061.

#### 5.2.22. 4-(Tert-butyl)-N-((4-methylquinazolin-2-yl)methyl)-N-(3,4,5-trimethoxyphenyl) benzene-sulfonamide (10g)

White powder, Yield, 64%, m.p. 144–145 °C. ^1^H NMR (400 MHz, DMSO-*d_6_*) δ 8.21 (d, *J* = 8.3 Hz, 1H), 7.97–7.90 (m,1H), 7.78 (d, *J* = 8.3 Hz, 1H), 7.71–7.63 (m,3H), 7.56 (d, *J* = 8.6 Hz, 2H), 6.60 (s,2H), 5.15 (s,2H), 3.59 (d, *J* = 2.4 Hz, 9H), 2.81 (s,3H), 1.29 (s,9H).^13^C NMR (101 MHz, DMSO-*d_6_*) δ 168.73, 160.70, 155.92, 152.27, 148.85, 136.93, 136.51, 135.60, 134.17, 127.76, 127.60, 127.52, 125.67, 125.63, 122.30, 107.10, 59.93, 57.32, 55.59, 34.82, 30.73, 21.32. HR-MS (ESI): Calcd, C_29_H_33_N_3_O_5_S [M + H]^+^:536.2219, found: 536.2218.

#### 5.2.23. 3,4-Dimethoxy-N-((4-methylquinazolin-2-yl)methyl)-N-(3,4,5-trimethoxyphenyl)benzene-sulfonamide (10h)

Light yellow powder, Yield, 42%, m.p. 129–130 °C.^1^H NMR (400 MHz, DMSO-*d_6_*) δ 8.22 (d, *J* = 8.3 Hz, 1H), 7.96 (t, *J* = 7.6 Hz, 1H), 7.84 (d, *J* = 8.3 Hz, 1H), 7.70 (t, *J* = 7.6 Hz, 1H), 7.30 (dd, *J* = 8.5, 1.8 Hz, 1H), 7.07 (dd, *J* = 14.1, 5.2 Hz, 2H), 6.69 (s,2H), 5.11 (s,2H), 3.83 (s,3H), 3.63 (d, *J* = 6.0 Hz, 9H), 3.60 (s,3H), 2.82 (s,3H).^13^C NMR (101 MHz, DMSO-*d_6_*) δ 168.66, 160.81, 152.36, 152.25, 148.89, 148.28, 136.92, 135.90, 134.21, 130.52, 127.77, 127.54, 125.66, 122.30, 121.50, 110.97, 110.22, 107.04, 59.95, 57.10, 55.87, 55.75, 55.56, 21.34. HR-MS (ESI) calcd, C_27_H_29_N_3_O_7_S [M + H]^+^:540.1804, found:540.1803.

#### 5.2.24. N-((4-Methylquinazolin-2-yl)methyl)-N-(3,4,5-trimethoxyphenyl)thiophene-2-sulphonamide (10i)

White powder, Yield, 60%, m.p. 151–152 °C. ^1^H NMR (400 MHz, DMSO-*d_6_*) δ 8.23 (d, *J* = 8.2 Hz, 1H), 8.02 (dd, *J* = 5.0, 1.2 Hz, 1H), 7.99–7.94 (m,1H), 7.88 (d, *J* = 8.2 Hz, 1H), 7.74–7.68 (m,1H), 7.67 (dd, *J* = 3.6, 1.2 Hz, 1H), 7.23 (dd, *J* = 4.9, 3.8 Hz, 1H), 6.70 (s,2H), 5.15 (s,2H), 3.62 (d, *J* = 15.1 Hz, 9H), 2.86 (s,3H).^13^C NMR (100 MHz, DMSO-*d_6_*) δ 168.86, 160.57, 152.36, 148.92, 139.33, 137.10, 135.27, 134.26, 133.55, 133.20, 127.81, 127.71, 127.57, 125.71, 122.36, 106.90, 59.94, 57.20, 55.75, 21.40. HR-MS (ESI) calcd, C_23_H_23_N_3_O_5_S_2_ [M + H]^+^:486.1157, found:486.1156.

#### 5.2.25. N-((4-Methylquinazolin-2-yl)methyl)-N-(3,4,5-trimethoxyphenyl)pyridine-2-sulphonamide (10j)

White powder, Yield, 63%, M.p. 159–160 °C. ^1^H NMR (400 MHz, DMSO-*d_6_*) δ 8.89 (t, *J* = 3.6 Hz, 1H), 8.87 (dd, *J* = 4.8, 1.5 Hz, 1H), 8.21 (d, *J* = 7.8 Hz, 1H), 8.19–8.13 (m,1H), 7.95 (ddd, *J* = 8.3, 7.0, 1.3 Hz, 1H), 7.74 (d, *J* = 8.2 Hz, 1H), 7.71–7.67 (m,1H), 7.64 (dd, *J* = 8.0, 4.9 Hz, 1H), 6.67 (s,2H), 5.24 (s,2H), 3.60 (d, *J* = 0.9 Hz, 9H), 2.80 (s,3H).^13^C NMR (101 MHz, DMSO-*d_6_*) δ 168.93, 160.46, 153.29, 152.50, 148.78, 147.97, 137.24, 136.11, 135.68, 134.90, 134.34, 127.63, 125.73, 123.95, 122.33, 107.13, 59.94, 57.33, 55.73, 21.31. HR-MS (ESI) calcd, C_24_H_24_N_4_O_5_S [M + H]^+^:481.1546, found:481.1545.

#### 5.2.26. N-((4-Methylquinazolin-2-yl)methyl)-N-(3,4,5-trimethoxyphenyl) benzamide (11a)

Light yellow powder, Yield, 55%, m.p. 112–113 °C. ^1^H NMR (400 MHz, DMSO-*d_6_*) ^1^H NMR (400 MHz, DMSO) δ 8.23 (d, *J* = 7.7 Hz, 1H), 7.96 (ddd, *J* = 8.3, 6.9, 1.3 Hz, 1H), 7.82 (d, *J* = 8.1 Hz, 1H), 7.78 (s, 1H), 7.76 (d, *J* = 1.3 Hz, 1H), 7.70 (t, *J* = 7.4 Hz, 2H), 7.58 (t, *J* = 7.7 Hz, 2H), 6.61 (s, 2H), 5.16 (s, 2H), 3.59 (d, *J* = 1.6 Hz, 9H), 2.83 (s, 3H).^13^C NMR (101 MHz, DMSO) δ 168.82, 160.72, 152.29, 148.87, 139.32, 136.94, 135.41, 134.27, 132.92, 128.92, 127.76, 127.64, 127.57, 125.72, 122.34, 107.00, 59.93, 57.22, 55.67, 30.66, 21.37.HR-MS (ESI) calcd, C_26_H_25_N_3_O_4_ [M + H]^+^:444.1923, found:444.1924.

#### 5.2.27. 4-Fluoro-N-((4-methylquinazolin-2-yl)methyl)-N-(3,4,5-trimethoxyphenyl) benzamide (11b)

White powder, Yield, 64%, M.p. 162–163 °C. ^1^H NMR (400 MHz, DMSO-*d_6_*) δ 8.24 (d, *J* = 8.3 Hz, 1H), 7.95 (t, *J* = 6.2 Hz, 2H), 7.77–7.59 (m,1H), 7.58–7.41 (m,2H), 7.14 (t, *J* = 8.9 Hz, 2H), 6.77 (s,2H), 5.33 (s,2H), 3.58 (d, *J* = 9.4 Hz, 9H), 2.93 (s,3H). ^13^C NMR (101 MHz, DMSO-*d_6_*) δ 168.85, 163.47, 161.54, 161.01, 152.41, 149.12, 139.59, 135.83, 134.20, 133.19, 133.16, 130.34, 130.26, 127.88, 127.28, 125.70, 122.41, 114.84, 114.63, 105.85, 59.99, 55.77, 21.55. HR-MS (ESI) calcd, C_26_H_24_FN_3_O_4_ [M + Na]^+^:484.1649, found:484.1645.

#### 5.2.28. 4-Bromo-N-((4-methylquinazolin-2-yl)methyl)-N-(3,4,5-trimethoxyphenyl) benzamide (11c)

White powder, Yield, 54%, m.p. 182–183 °C. ^1^H NMR (400 MHz, DMSO-*d_6_*) δ 8.27 (d, *J* = 8.3 Hz, 1H), 8.00 (d, *J* = 16.6 Hz, 2H), 7.78–7.65 (m,1H), 7.52 (d, *J* = 8.3 Hz, 2H), 7.38 (d, *J* = 7.5 Hz, 2H), 6.77 (s,2H), 5.32 (s,2H), 3.58 (d, *J* = 8.8 Hz, 9H), 2.94 (s,3H). ^13^C NMR (101 MHz, DMSO-*d_6_*) δ 168.88, 161.45, 152.44, 149.15, 139.31, 136.04, 136.01, 134.21, 130.78, 129.84, 127.90, 127.32, 125.73, 122.64, 122.44, 105.98, 60.02, 55.84, 21.56. HR-MS (ESI) calcd, C_26_H_24_BrN_3_O_4_ [M + H]^+^: 522.1028, found:522.1029.

#### 5.2.29. 2-Chloro-N-((4-methylquinazolin-2-yl)methyl)-N-(3,4,5-trimethoxyphenyl) benzamide (11d)

White powder, Yield, 52%, m.p. 190–191 °C. ^1^H NMR (400 MHz, DMSO-*d_6_*) δ 8.28 (d, *J* = 8.3 Hz, 1H), 8.01 (d, *J* = 3.6 Hz, 2H), 7.75–7.69 (m,1H), 7.47–7.43 (m,1H), 7.42–7.36 (m,1H),7.31–7.23 (m,2H), 6.91 (s,2H), 5.32 (s,2H), 3.62 (s,6H), 3.51 (s,3H), 2.96 (s,3H). ^13^C NMR (101 MHz, DMSO-*d_6_*) δ 168.90, 167.21, 161.17, 152.16, 149.15, 138.20, 136.69, 136.12, 134.29, 130.07, 129.76, 129.12, 128.77, 127.83, 127.35, 126.65, 125.78, 122.46, 105.66, 59.90, 55.67, 54.64, 21.53. HR-MS (ESI) *m*/*z*: 478.1534 [M + H]^+^(calcd for 478.1533, C_26_H_24_ClN_3_O_4_).

#### 5.2.30. 4-Methyl-N-((4-methylquinazolin-2-yl)methyl)-N-(3,4,5-trimethoxyphenyl) benzamide (11e)

White powder, Yield, 51%, m.p. 196–197 °C. ^1^H NMR (400 MHz, DMSO-*d_6_*) δ 8.33 (d, *J* = 8.2 Hz, 1H), 8.09–7.95 (m,2H), 7.76 (ddd, *J* = 8.2, 6.0, 2.1 Hz, 1H), 7.36 (d, *J* = 7.8 Hz, 2H), 7.15 (d, *J* = 7.9 Hz, 2H), 6.80 (s,2H), 5.35 (s,2H), 3.62 (d, *J* = 6.2 Hz, 9H), 2.99 (s,3H), 2.31 (s,3H). ^13^C NMR (101 MHz, DMSO-*d_6_*) δ 168.81, 161.69, 152.30, 149.12, 138.91, 135.67, 134.22, 133.84, 128.23, 127.89, 127.85, 127.30, 125.75, 122.41, 105.87, 59.99, 55.75, 21.58, 20.84. HR-MS (ESI) calcd, C_27_H_27_N_3_O_4_ [M + Na]^+^:480.1899, found:480.1902.

#### 5.2.31. 2,4,6-Trimethyl-N-((4-methylquinazolin-2-yl)methyl)-N-(3,4,5-trimethoxyphenyl) benzamide (11f)

White powder, Yield, 44%, m.p. 209–210 °C. ^1^H NMR (400 MHz, DMSO-*d_6_*) δ 8.28 (d, *J* = 8.3 Hz, 1H), 8.02–7.97 (m,1H), 7.93 (d, *J* = 7.9 Hz, 1H), 7.72 (t, *J* = 7.6 Hz, 1H), 6.80 (s,2H), 6.74 (s, 2H), 5.34 (s,2H), 3.59 (s,6H), 3.51 (s,3H), 2.94 (s,3H), 2.37 (s,6H), 2.15 (s,3H).^13^C NMR (101 MHz, DMSO-*d_6_*) δ 151.89, 138.18, 137.03, 134.41, 134.34, 133.51, 127.57, 127.49, 125.83, 104.93, 59.91, 55.62, 54.77, 21.44, 20.55, 19.26. HR-MS (ESI) calcd, C_29_H_31_N_3_O_4_ [M + Na]^+^:508,2212, found:508.2209.

#### 5.2.32. 4-(Tert-butyl)-N-((4-methylquinazolin-2-yl)methyl)-N-(3,4,5-trimethoxyphenyl) benzamide (11g)

White powder, Yield, 47%, m.p. 117–118 °C. ^1^H NMR (400 MHz, DMSO-*d_6_*) δ 8.25 (d, *J* = 8.3 Hz, 1H), 8.02–7.89 (m,2H), 7.69 (ddd, *J* = 8.2, 5.6, 2.5 Hz, 1H), 7.33 (dd, *J* = 17.6, 8.2 Hz, 4H), 6.72 (s,2H), 5.33 (s,2H), 3.55 (d, *J* = 1.7 Hz, 9H), 2.93 (s,3H), 1.22 (s,9H).^13^C NMR (101 MHz, DMSO-*d_6_*) δ 169.77, 168.77, 161.70, 152.32, 152.02, 149.15, 139.76, 135.76, 134.17, 133.81, 127.90, 127.72, 127.25, 125.70, 124.39, 122.41, 105.92, 59.99, 55.78, 34.39, 30.85, 21.56. HR-MS (ESI) calcd, C_30_H_33_N_3_O_4_ [M + H]^+^:500.2549, found:500.2548.

#### 5.2.33. 4-Methoxy-N-((4-methylquinazolin-2-yl)methyl)-N-(3,4,5-trimethoxyphenyl) benzamide (11h)

White powder, Yield, 61%, m.p0.165–166 °C. ^1^H NMR (400 MHz, DMSO-*d_6_*) δ 8.25 (d, *J* = 8.2 Hz, 1H), 8.00–7.93 (m,2H), 7.69 (ddd, *J* = 8.2, 5.9, 2.2 Hz, 1H), 7.38 (d, *J* = 8.7 Hz, 2H), 6.84 (d, *J* = 8.8 Hz, 2H), 6.75 (s,2H), 5.31 (s,2H), 3.73 (s,3H), 3.58 (d, *J* = 6.3 Hz, 9H), 2.93 (s,3H). ^13^C NMR (101 MHz, DMSO-*d_6_*) δ 169.37, 168.73, 161.82, 159.98, 152.39, 149.15, 140.10, 135.76, 134.15, 129.86, 128.67, 127.89, 127.24, 125.69, 122.41, 113.00, 105.89, 60.01, 55.89, 55.81, 55.12, 21.55. HR-MS (ESI) calcd, C_27_H_27_N_3_O_5_ [M + Na]^+^: 496.1848, found:496.1844.

#### 5.2.34. 3,4,5-Trimethoxy-N-((4-methylquinazolin-2-yl)methyl)-N-(3,4,5-trimethoxyphenyl) benzamide (11i)

White powder, Yield, 66%, m.p. 129–130 °C. ^1^H NMR (400 MHz, DMSO-*d_6_*) δ 8.25 (d, *J* = 8.2 Hz, 1H), 7.97 (d, *J* = 3.7 Hz, 2H), 7.76–7.59 (m, 1H), 6.83 (s, 2H), 6.77 (s, 2H), 5.33 (s, 2H), 3.65 (d, *J* = 2.2 Hz, 9H), 3.61 (s, 6H), 3.58 (s, 3H), 2.94 (s, 3H).^13^C NMR (101 MHz, DMSO-*d_6_*) δ 169.17, 168.80, 161.68, 152.50, 152.09, 149.13, 139.96, 138.34, 136.01, 134.21, 131.51, 127.87, 127.28, 125.70, 122.41, 106.02, 60.03, 59.98, 55.91, 55.79, 21.53. HRMS (ESI): 534.2240 [M + H]^+^ (calcd for 534.2241, C_29_H_31_N_3_O_7_).

#### 5.2.35. N-((4-methylquinazolin-2-yl)methyl)-N-(3,4,5-trimethoxyphenyl)thiophene-2-carboxamide (11j)

White solid, Yield, 48%, m.p. 136–137 °C.^1^H NMR (400 MHz, DMSO-*d_6_*) δ 8.26 (d, *J* = 8.3 Hz, 1H), 7.96 (d, *J* = 3.6 Hz, 2H), 7.69 (dd, *J* = 10.9, 4.7 Hz, 2H), 7.07 (s,2H), 6.97 (dd, *J* = 5.8, 2.9 Hz, 1H), 6.87 (s,1H), 5.26 (s,2H), 3.70 (s,6H),3.67 (d, *J* = 1.8 Hz, 3H),2.92 (s,3H).^13^C NMR (101 MHz, DMSO-*d_6_*) δ 168.83, 161.52, 161.48, 153.03, 149.07, 138.56, 137.56, 137.53,134.21,131.96,131.71,127.84,127.29,127.00, 125.74, 122.41, 107.19, 60.21, 56.47, 55.97, 21.56. HRMS (ESI): 472.1307 [M + Na]^+^ (calcd for 472.1310, C_24_H_23_N_3_O_4_S).

#### 5.2.36. 4-Methoxy-N-((4-methylquinazolin-2-yl)methyl)-N-phenylbenzene -sulphonamide (14a)

White powder, Yield, 66%, m.p. 85–86 °C. ^1^H NMR (400 MHz, DMSO-*d_6_*) δ 8.21 (d, *J* = 8.2 Hz, 1H), 7.94 (t, *J* = 7.6 Hz, 1H), 7.85 (d, *J* = 8.2 Hz, 1H), 7.68 (dd, *J* = 13.8, 8.1 Hz, 3H), 7.32–7.16 (m, 5H), 7.08 (d, *J* = 8.9 Hz, 2H), 5.13 (s, 2H), 3.84 (s, 3H), 2.84 (s, 3H). ^13^C NMR (101 MHz, DMSO-*d_6_*) δ 168.81, 162.53, 160.71, 148.91, 140.05, 134.14, 130.45, 129.63, 128.69, 128.44, 127.92, 127.53, 127.39, 125.64, 122.31, 114.15, 56.68, 55.66, 21.41.HR-MS (ESI): Calcd, C_23_H_21_N_3_O_3_S, [M + H]^+^: 420.1376, found: 420.1376.

#### 5.2.37. N-(4-bromophenyl)-4-methoxy-N-((4-methylquinazolin-2-yl)methyl)benzene sulphonamide (14b)

White powder, Yield, 66%, m.p. 169–170 °C. ^1^H NMR (400 MHz, DMSO-*d_6_*) δ 8.21 (d, *J* = 7.9 Hz, 1H), 7.95 (ddd, *J* = 8.3, 6.9, 1.3 Hz, 1H), 7.84 (d, *J* = 8.1 Hz, 1H), 7.73–7.60 (m, 3H), 7.37–7.27 (m, 4H), 7.14–7.03 (m, 2H), 5.12 (s, 2H), 3.84 (s, 3H), 2.84 (s, 3H). ^13^C NMR (101 MHz, DMSO-*d_6_*) δ 168.93, 162.65, 160.47, 148.87, 138.96, 134.20, 131.85, 130.19, 130.02, 129.62, 128.73, 127.89, 127.60, 125.66, 122.32, 114.27, 56.41, 55.67, 21.40.HR-MS (ESI): Calcd, C_23_H_20_ClN_3_O_3_S, [M + H]^+^: 454.0987, found: 454.0983.

#### 5.2.38. N-(4-chlorophenyl)-4-methoxy-N-((4-methylquinazolin-2-yl)methyl)benzene-sulphonamide (14c)

White powder, Yield, 66%, m.p. 167–168 °C. ^1^H NMR (400 MHz, DMSO-*d_6_*) δ 8.20 (d, *J* = 8.2 Hz, 1H), 7.94 (t, *J* = 7.2 Hz, 1H), 7.84 (d, *J* = 8.2 Hz, 1H), 7.68 (dd, *J* = 14.6, 8.0 Hz, 3H), 7.48 (d, *J* = 8.7 Hz, 2H), 7.25 (d, *J* = 8.7 Hz, 2H), 7.08 (d, *J* = 8.9 Hz, 2H), 5.12 (s, 2H), 3.84 (s, 3H), 2.84 (s, 3H). ^13^C NMR (101 MHz, DMSO-*d_6_*) δ 168.85, 162.49, 160.67, 148.85, 137.23, 136.98, 134.18, 130.41, 129.62, 129.22, 128.41, 127.86, 127.58, 125.59, 122.28, 114.12, 56.76, 55.62, 21.35, 20.44. HR-MS (ESI): Calcd, C_23_H_20_BrN_3_O_3_S, [M + H]^+^: 498.0482, found: 498.0492.

#### 5.2.39. 4-Methoxy-N-((4-methylquinazolin-2-yl)methyl)-N-(p-tolyl)benzene-sulphonamide (14d)

White powder, Yield, 66%, m.p. 129–130 °C. ^1^H NMR (400 MHz, DMSO-*d_6_*) δ 8.18 (d, *J* = 8.2 Hz, 1H), 7.96–7.80 (m, 2H), 7.66 (dd, *J* = 11.8, 8.4 Hz, 3H), 7.07 (dt, *J* = 15.5, 8.3 Hz, 6H), 5.07 (s, 2H), 3.83 (s, 3H), 2.83 (s, 3H), 2.18 (s, 3H). ^13^C NMR (101 MHz, DMSO-*d_6_*) δ 168.92, 162.65, 160.46, 148.87, 139.43, 134.19, 131.68, 130.45, 130.02, 129.62, 127.89, 127.59, 125.65, 122.32, 120.34, 114.28, 56.34, 55.66, 21.40. HR-MS (ESI): Calcd, C_24_H_23_N_3_O_3_S, [M + H]^+^: 434.1533, found: 434.1538.

### 5.3. Cell culture

Cell lines used were cultured in humidified incubator in a humidified atmosphere of 5% CO2 and 95% air. The RPMI-1640 medium was supplemented with 10% foetal bovine serum, penicillin (100 U/ml) and streptomycin (0.1 mg/ml).

### 5.4. MTT assay

Cell lines were seeded into 96-well plates and incubated for 24 h. Then cells were treated with different concentrations of compounds. And after another 48 h, MTT reagent (20 μL per well) was added to each well and then incubated at 37 °C for 4 h. After the suspension was discarded, formazan was then dissolved with DMSO. Absorbencies of formazan solution were measured at 490 nm. The IC_50_ values of tested compounds were calculated by SPSS version 17.0.

### 5.5. Western blotting analysis

2 gastric cancer cells were seeded in dishes and treated with **9i** or DMSO. After 48 h, MGC-803 and SGC-7901 cells were collected and then lysed. The denatured lysates of each groups were electrophoretic separated in SDS-PAGE. Proteins were then transferred onto PVDF membranes from gels. After blocking for 2 h, membranes were incubated with primary antibodies conjugation. Then, the membranes were washed and incubated with 2^nd^ antibodies. At last, specific proteins were detected.

### 5.6. Xenograft studies

A human gastric cancer subcutaneous transplantation tumour model was established with MGC-803 cells. After the tumour volume reaches 100 mm3 the MGC-803 bearing mice were randomised into 3 groups and intraperitoneal injection with normal saline or drugs continuously for 21 days. Tumour volume was measured every 3 days (Length × Width2/2). Mice were executed after treatment. 500 μL blood samples was collected each mouse, samples were then centrifuged at 2000 g for 10 min for biochemistry analysis. Tumour and organs tissues were harvested for H&E staining or immunochemistry detection. The experiments in the study were performed comply with the protocols approved by the Institutional Animal Care and Use Committee, Zhengzhou University.

### 5.7. General methods

In this work, some other assays including colony formation assay, cell apoptosis assay and immunostaining assay were referred to our previous work^35^^,^^36^.

## Supplementary Material

Supplemental Material

## References

[1] Harvey KF, Pfleger CM, Hariharan IK. The Drosophila Mst ortholog, hippo, restricts growth and cell proliferation and promotes apoptosis. Cell 2003;114:457–67. 12941274 10.1016/s0092-8674(03)00557-9

[2] Huang J, Wu S, Barrera J, et al. The Hippo signaling pathway coordinately regulates cell proliferation and apoptosis by inactivating Yorkie, the Drosophila homolog of YAP. Cell 2005;122:421–34. 16096061 10.1016/j.cell.2005.06.007

[3] Staley BK, Irvine KD. Hippo signaling in Drosophila: recent advances and insights. Dev Dyn 2012;241:3–15. 22174083 10.1002/dvdy.22723 PMC3426292

[4] Plouffe SW, Hong AW, Guan K. Disease implications of the Hippo/YAP pathway. Trends Mol Med 2015;21:212–22. 25702974 10.1016/j.molmed.2015.01.003 PMC4385444

[5] Harvey KF, Zhang X, Thomas DM. The Hippo pathway and human cancer. Nat Rev Cancer 2013;13:246–57. 23467301 10.1038/nrc3458

[6] Hong AW, Meng Z, Guan K. The Hippo pathway in intestinal regeneration and disease. Nat Rev Gastroenterol Hepatol 2016;13:324–37. 27147489 10.1038/nrgastro.2016.59 PMC5642988

[7] Zhao B, Tumaneng K, Guan K. The Hippo pathway in organ size control, tissue regeneration and stem cell self-renewal. Nat Cell Biol 2011;13:877–83. 21808241 10.1038/ncb2303 PMC3987945

[8] Yu F, Guan K. The hippo pathway: regulators and regulations. Genes Dev 2013;27:355–71. 23431053 10.1101/gad.210773.112 PMC3589553

[9] Li H-L, Li Q-Y, Jin M-J, et al. A review: hippo signaling pathway promotes tumor invasion and metastasis by regulating target gene expression. J Cancer Res Clin Oncol 2021;147:1569–85., 33864521 10.1007/s00432-021-03604-8 PMC11801896

[10] Chen C, Zhu D, Zhang H, et al. YAP-dependent ubiquitination and degradation of β-catenin mediates inhibition of Wnt signalling induced by Physalin F in colorectal cancer. Cell Death Dis 2018;9:591 29789528 10.1038/s41419-018-0645-3 PMC5964149

[11] Kim M, Kim T, Johnson RL, Lim D. Transcriptional co-repressor function of the hippo pathway transducers YAP and TAZ. Cell Rep 2015;11:270–82. 25843714 10.1016/j.celrep.2015.03.015

[12] Dupont S, Morsut L, Aragona M, et al. Role of YAP/TAZ in mechanotransduction. Nature 2011;474:179–183. 21654799 10.1038/nature10137

[13] Moroishi T, Hansen CG, Guan K. The emerging roles of YAP and TAZ in cancer. Nat Rev Cancer 2015;15:73–9. 25592648 10.1038/nrc3876 PMC4562315

[14] Zhao C, Zeng C, Ye S, et al. Yes-associated protein (YAP) and transcriptional coactivator with a PDZ-binding motif (TAZ): a nexus between hypoxia and cancer. Acta Pharma Sin B 2020;10:947–60. 10.1016/j.apsb.2019.12.010 PMC7332664 32642404

[15] Han Y. Analysis of the role of the Hippo pathway in cancer. J Transl Med 2019;17:116–7. 30961610 10.1186/s12967-019-1869-4 PMC6454697

[16] Maugeri-Saccà M, Barba M, Pizzuti L, et al. The Hippo transducers TAZ and YAP in breast cancer: oncogenic activities and clinical implications. Expert Rev Mol Med 2015;17:e14. 26136233 10.1017/erm.2015.12

[17] Wang Y, Dong Q, Zhang Q, et al. Overexpression of yes-associated protein contributes to progression and poor prognosis of non-small-cell lung cancer. Cancer Sci 2010;101:1279–1285. 20219076 10.1111/j.1349-7006.2010.01511.x PMC11158334

[18] Wang L, Shi S, Guo Z, et al. Overexpression of YAP and TAZ is an independent predictor of prognosis in colorectal cancer and related to the proliferation and metastasis of colon cancer cells. PLOS One 2013;8:e65539. 23762387 10.1371/journal.pone.0065539 PMC3677905

[19] Qiao Y, Li T, Zheng S, Wang H. The Hippo pathway as a drug target in gastric cancer. Cancer Lett 2018;420:14–25. 29408652 10.1016/j.canlet.2018.01.062

[20] Zhang L, Yang S, Chen X, et al. The Hippo pathway effector YAP regulates motility, invasion, and castration-resistant growth of prostate cancer cells. Mol Cell Biol 2015;35:1350–62. 25645929 10.1128/MCB.00102-15 PMC4372700

[21] Zhao B, Wei X, Li W, et al. Inactivation of YAP oncoprotein by the Hippo pathway is involved in cell contact inhibition and tissue growth control. Genes Dev 2007;21:2747–2761. 17974916 10.1101/gad.1602907 PMC2045129

[22] Johnson RL, Halder G. The two faces of Hippo: targeting the Hippo pathway for regenerative medicine and cancer treatment. Nat Rev Drug Discov 2014;13:63–79. 24336504 10.1038/nrd4161 PMC4167640

[23] Park HW, Guan K. Regulation of the Hippo pathway and implications for anticancer drug development. Trends Pharma Sci 2013;34:581–9. 10.1016/j.tips.2013.08.006 PMC3929107 24051213

[24] Nakatani K, Maehama T, Nishio M, et al. Targeting the Hippo signalling pathway for cancer treatment. J Biochem 2016;161:237–44. 10.1093/jb/mvw074 28003431

[25] Smith S, Sessions RB, Shoemark DK, et al. Antiproliferative and antimigratory effects of a novel YAP-TEAD interaction inhibitor identified using *in silico* molecular docking. J Med Chem 2019;62:1291–305. 30640473 10.1021/acs.jmedchem.8b01402 PMC6701825

[26] Dokla EME, Fang C, Chu P, et al. Targeting YAP degradation by a novel 1,2,4-oxadiazole derivative via restoration of the function of the Hippo pathway. ACS Med Chem Lett 2020;11:426–32. 32292545 10.1021/acsmedchemlett.9b00501 PMC7153020

[27] Lu W, Wang J, Li Y, et al. Discovery and biological evaluation of vinylsulfonamide derivatives as highly potent, covalent TEAD autopalmitoylation inhibitors. Eur J Med Chem 2019;184:111767. 31622854 10.1016/j.ejmech.2019.111767

[28] Qiao J, Lin G, Xia A, et al. Discovery of 1,8-disubstituted-[1,2,3]triazolo[4,5-*c*]quinoline derivatives as a new class of Hippo signaling pathway inhibitors. Bioorg Med Chem Lett 2019;29:2595–603. 31400941 10.1016/j.bmcl.2019.08.001

[29] Lei D, Chengcheng L, Xuan Q, et al. Quercetin inhibited mesangial cell proliferation of early diabetic nephropathy through the Hippo pathway. Pharmacol Res 2019;146:104320. 31220559 10.1016/j.phrs.2019.104320

[30] Zhang Y, Xin C, Qiu J, Wang Z. Essential oil from pinus koraiensis pinecones inhibits gastric cancer cells via the HIPPO/YAP signaling pathway. Molecules 1975;24:1639–41. 10.3390/molecules24213851 PMC6864528 31731517

[31] Fu D-J, Yang J-J, Li P, et al. Bioactive heterocycles containing a 3,4,5-trimethoxyphenyl fragment exerting potent antiproliferative activity through microtubule destabilization. Eur J Med Chem 2018;157:50–61. 30075402 10.1016/j.ejmech.2018.07.060

[32] Song J, Gao Q-L, Wu B-W, et al. Discovery of tertiary amide derivatives incorporating benzothiazole moiety as anti-gastric cancer agents *in vitro* via inhibiting tubulin polymerization and activating the Hippo signaling pathway. Eur J Med Chem 2020;203:112618. 32682200 10.1016/j.ejmech.2020.112618

[33] Hagras M, Deeb MRAE, Elzahabi HSA, et al. Discovery of new quinolines as potent colchicine binding site inhibitors: design, synthesis, docking studies, and anti-proliferative evaluation. J Enzyme Inhibit Med Chem 2021;36:640–58. 10.1080/14756366.2021.1883598 PMC7889231 33588683

[34] El-Naggar AM, Eissa IH, Belal A, El-Sayed AA. Design, eco-friendly synthesis, molecular modeling and anticancer evaluation of thiazol-5(4*H*)-ones as potential tubulin polymerization inhibitors targeting the colchicine binding site. RSC Adv 2020;10:2791–811. 10.1039/c9ra10094f PMC9048505 35496078

[35] Jian S, Gao Q-L, Wu B-W, et al. Novel tertiary sulfonamide derivatives containing benzimidazole moiety as potent anti-gastric cancer agents: design, synthesis and SAR studies. Eur J Med Chem 2019;183:111731. 31577977 10.1016/j.ejmech.2019.111731

[36] Song J, Cui X, Wu B, et al. Discovery of 1,2,4-triazine-based derivatives as novel neddylation inhibitors and anticancer activity studies against gastric cancer MGC-803 cells. Bioorg Med Chem Lett 2020;30:126791. 31740251 10.1016/j.bmcl.2019.126791

