# Supplementary material for: Discovery of N-aryl sulphonamide-quinazoline derivatives as anti-gastric cancer agents in vitro and in vivo via activating the Hippo signalling pathway
Source: J Enzyme Inhib Med Chem. 2021 Aug 23;36(1):1715–31. doi: 10.1080/14756366.2021.1958211 (PMC8386742; doi:10.1080/14756366.2021.1958211)
Supplement: Supplemental Material [file IENZ_A_1958211_SM0265.pdf]

Supplementary Information for

ORIGINAL ARTICLE

Discovery of N-aryl sulfonamide-quinazoline derivatives as anti-gastric cancer agents in vitro and in vivo via activating the Hippo signaling pathway

Jin-Bo Niu[a, 1, *], Jian Song[c, 1, *], Yuan Liu[c, 1], Chun-Hua Quan[c, 1], Guang-Xi Yu[b], Jia-Jia Yang[e], Yin-Ru Li[a], Yan-Bing Zhang[b], Ying-Qiu Qi[b], Cheng-Yun Jin[a, c, *], Sai-Yang Zhang[a, b, d, *]

a. The Third Affiliated Hospital of Zhengzhou University, Zhengzhou, 450001, China

b. School of Basic Medical Sciences, Zhengzhou University, Zhengzhou 450001, China

c. School of Pharmaceutical Sciences, Institute of Drug Discovery & Development, Key Laboratory of Advanced Drug Preparation Technologies (Ministry of Education), Zhengzhou University, Zhengzhou 450001, China

d. Henan Institute of Advanced Technology, Zhengzhou University, Zhengzhou 450001, China

e. Department of pharmacy, Zhengzhou people's Hospital, Zhengzhou 450001, China

* Corresponding authors: Jin-Bo Niu (niujinbo@zzu.edu.cn), Jian Song (mumuandzz@163.com), Cheng-Yun Jin (cyjin@zzu.edu.cn), Sai-Yang Zhang (saiyangz@zzu.edu.cn)

[1] These authors contributed equally to this work

* Corresponding authors: cyjin@zzu.edu.cn, saiyangz@zzu.edu.cn

1 These authors contributed equally to this work

In this work, compound **9i** was design and synthesized by SAR study of our reported tertiary amide derivatives compound **1** and compound **2**. Therefore, we tested the influence of compound **9i** on tubulin polymerization and AKT/mTOR and ERK pathways. As showed in **Supporting Figure S1**, compound **9i** shows no strong influence on tubulin polymerization and P38, ERK, AKT, mTOR and Wnt pathways. Fortunately, we found compound **9i** showed an obvious activation activity on hippo pathway. Therefore, we here reported N-sulfonamide-quinazoline derivatives as novel anti-gastric cancer agents via activating the Hippo signaling pathway *in vitro* and *in vivo*.

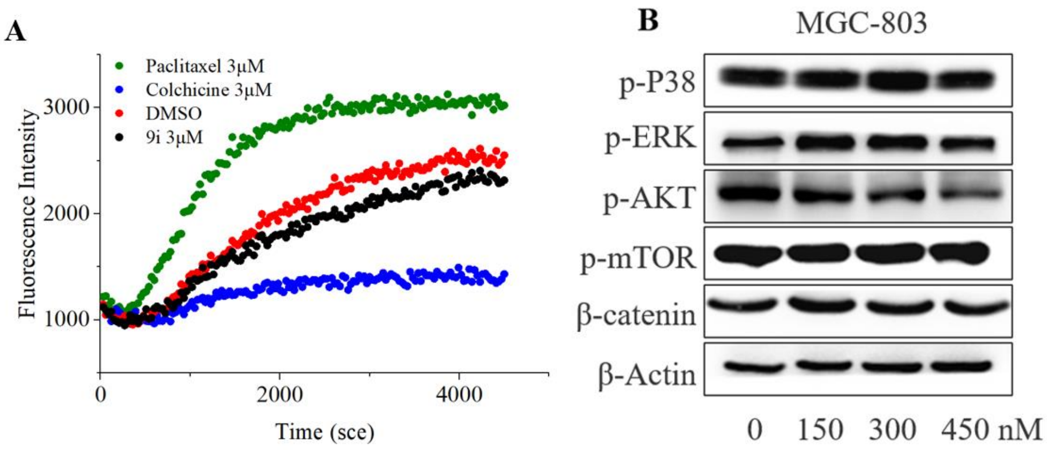

Supporting Figure S1. A. Activity of compound **9i** on tubulin polymerization. Tubulin polymerization extracellular, concentrations of Paclitaxel, Colchicine and **9i** were 3 μmol/L; Activity of compound **9i** on P38, ERK, AKT, mTOR and Wnt pathways, cells were incubated with 20, 40 and 60 nM **9i** for 48 hours

- ^{1}H, ^{13}C-NMR and HRMS of Compound **6a**

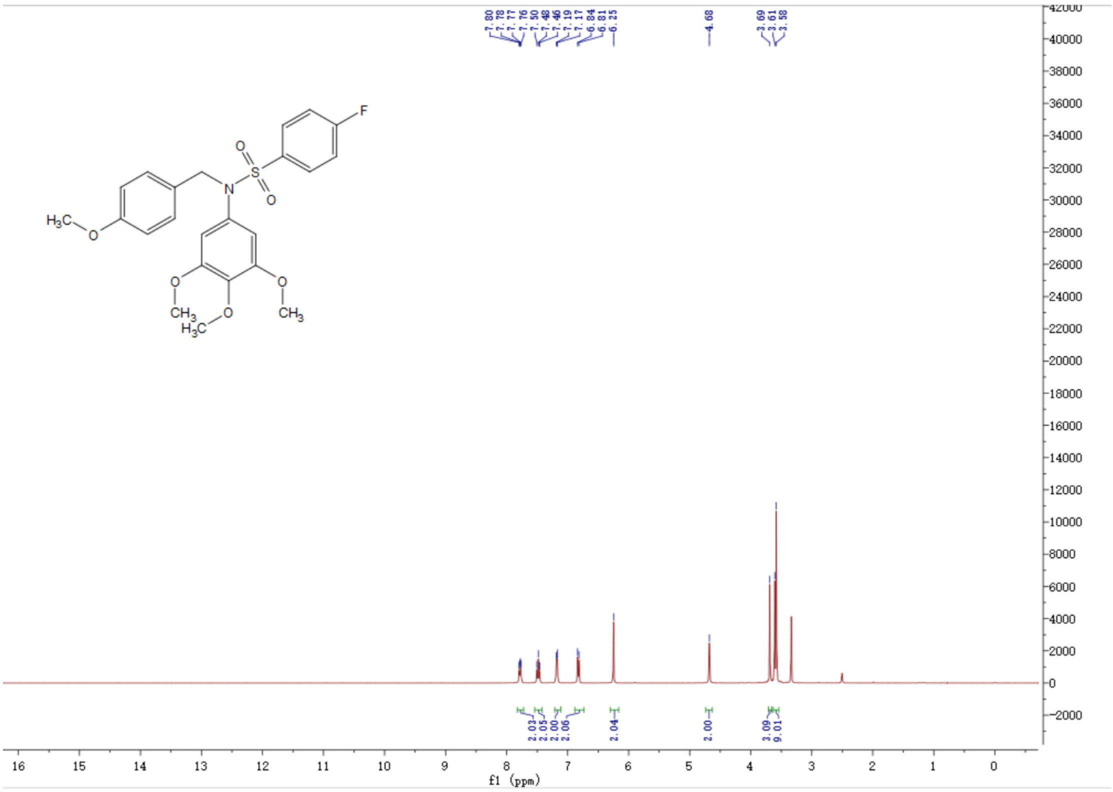

Figure S2. ^{1}H NMR spectrum of compound **6a** (400 MHz, DMSO-*d*6)

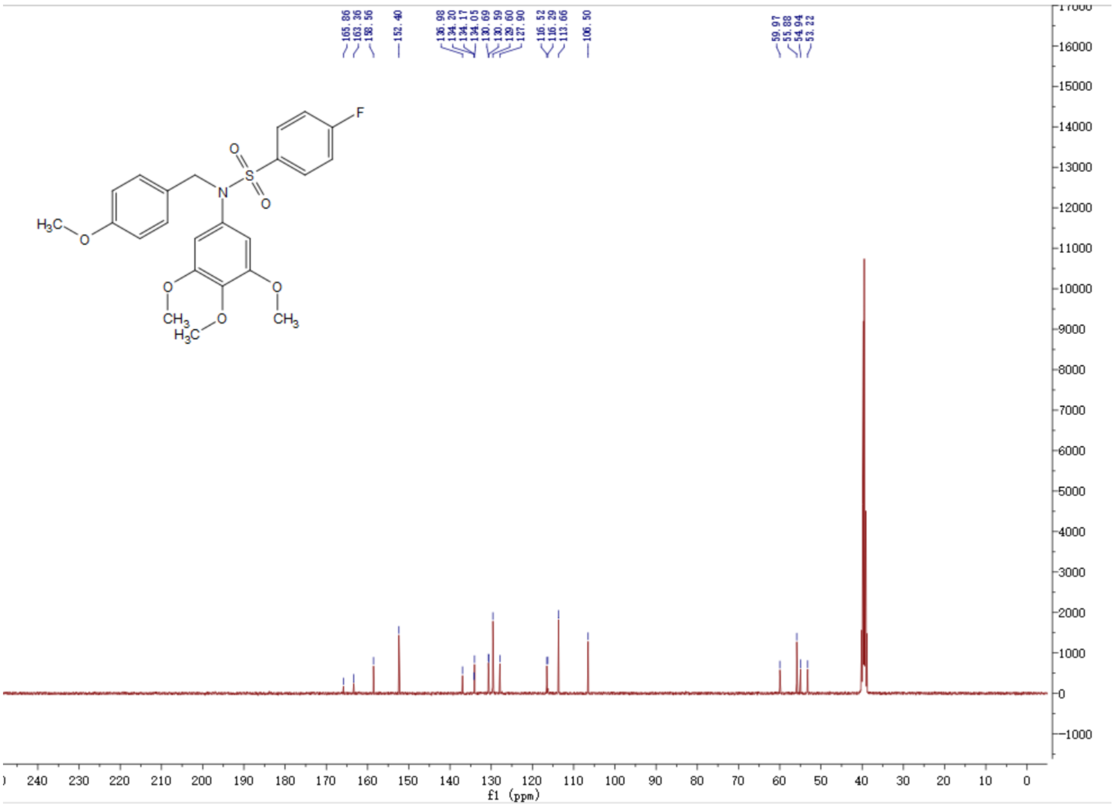

Figure S3. ^{13}C NMR spectrum of compound **6a** (100 MHz, DMSO-*d*6)

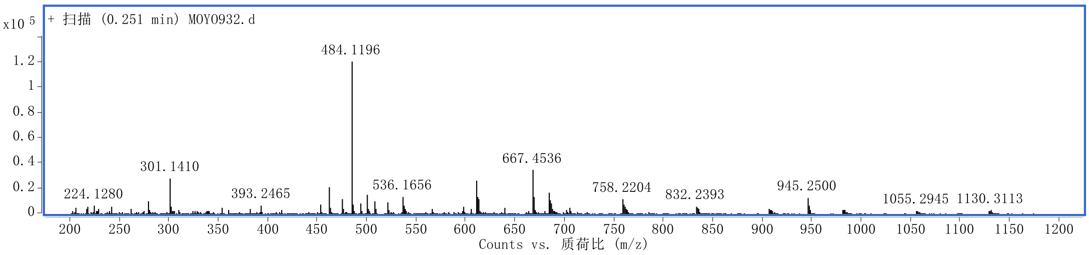

Figure S4. HRMS spectrum of compound **6a**

- ^{1}H, ^{13}C-NMR and HRMS of Compound **6b**

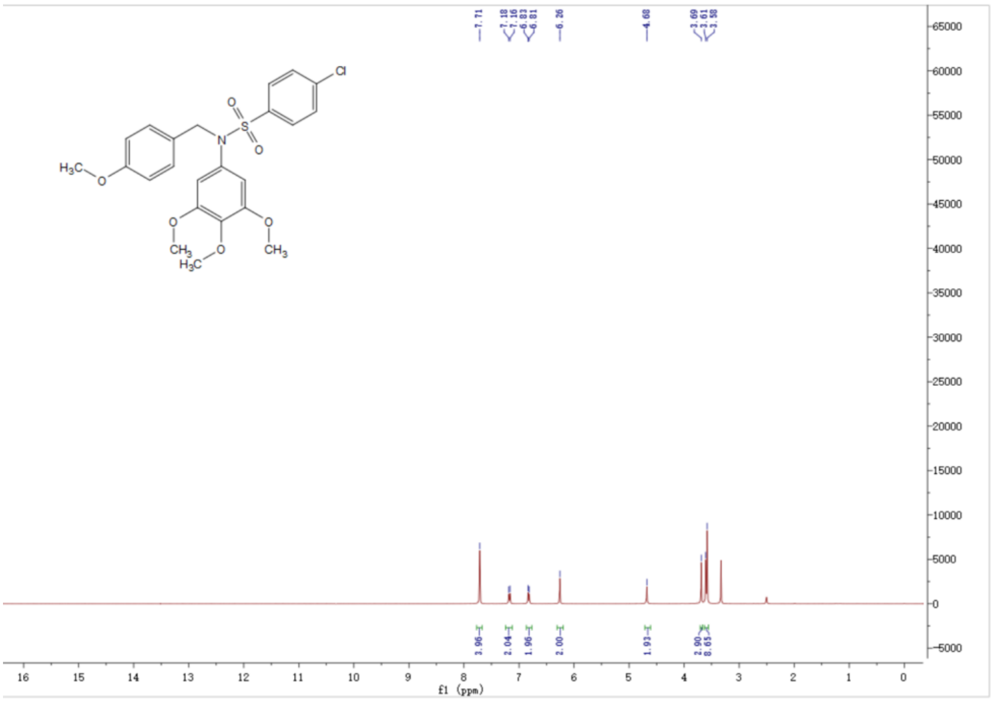

Figure S5. ^{1}H NMR spectrum of compound **6b** (400 MHz, DMSO-d_6)

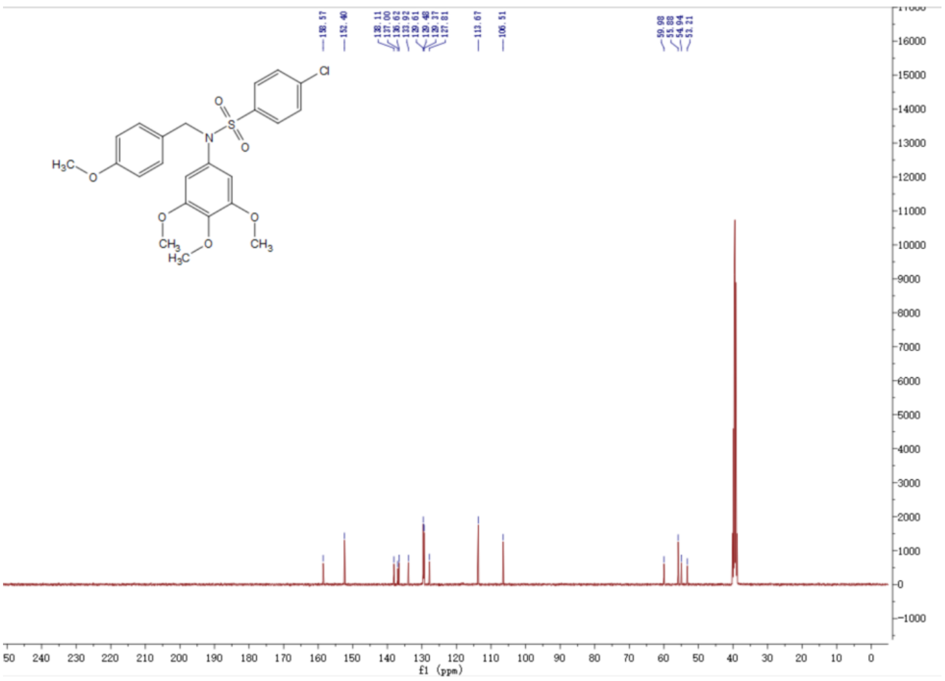

Figure S6. ^{13}C NMR spectrum of compound **6b** (100 MHz, DMSO-d_6)

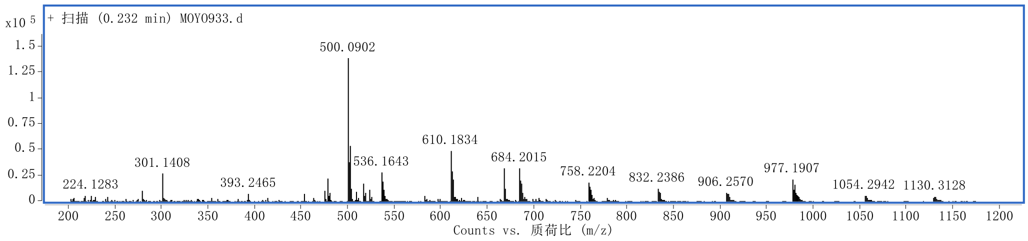

Figure S7. HRMS spectrum of compound **6b**

- ^{1}H, ^{13}C-NMR and HRMS of Compound **6c**

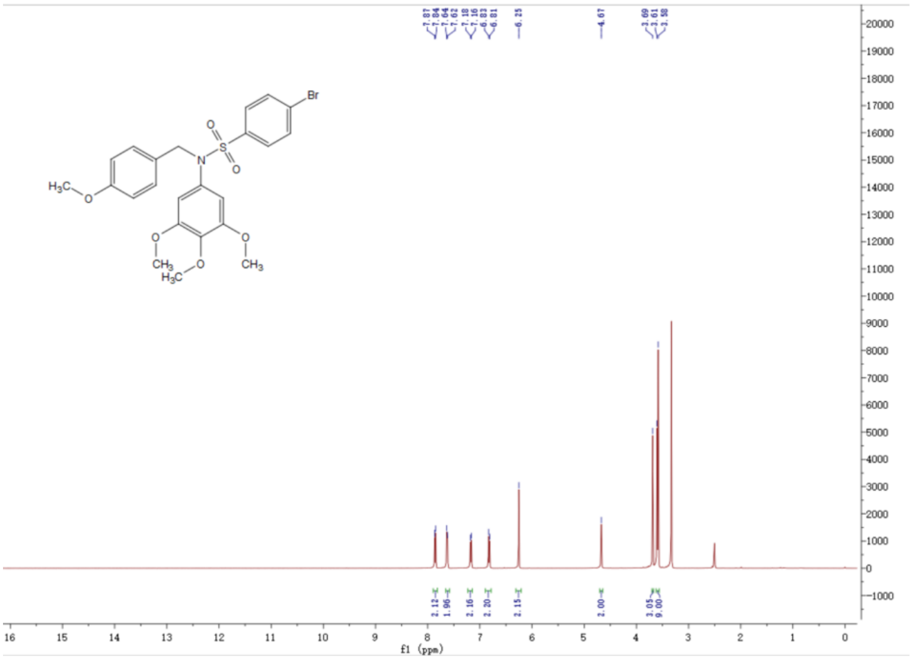

Figure S8. ^{1}H NMR spectrum of compound **6c** (400 MHz, DMSO-d_6)

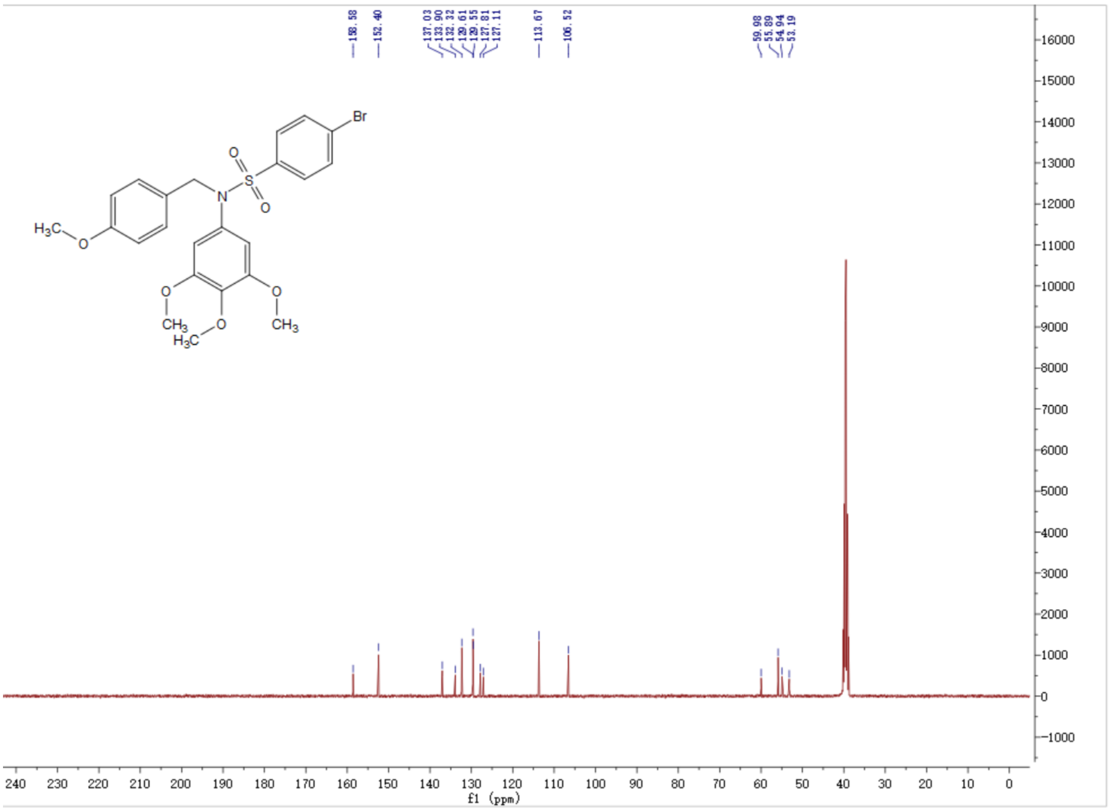

Figure S9. ^{13}C NMR spectrum of compound **6c** (100 MHz, DMSO-d_6)

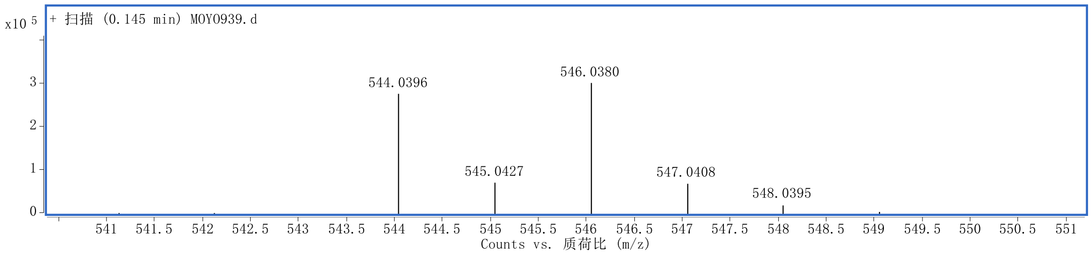

Figure S10. HRMS spectrum of compound **6c**

- ^{1}H, ^{13}C-NMR and HRMS of Compound **6e**

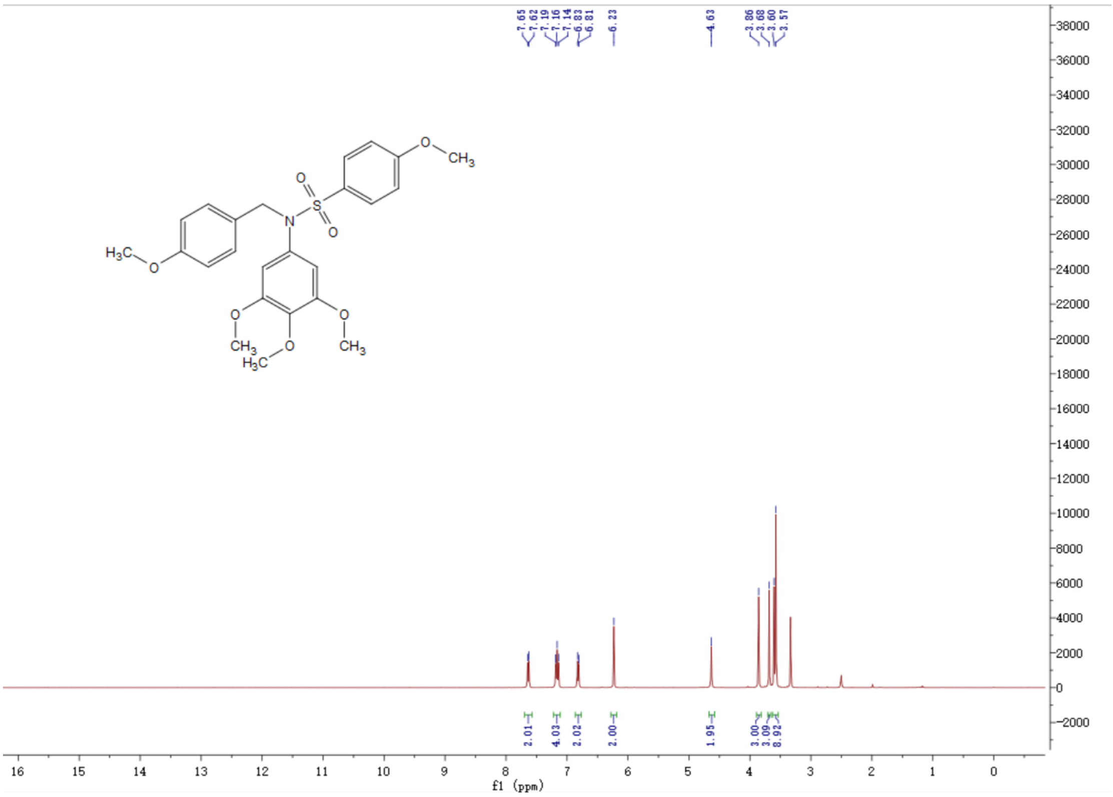

Figure S11. ^{1}H NMR spectrum of compound **6e** (400 MHz, DMSO-d_6)

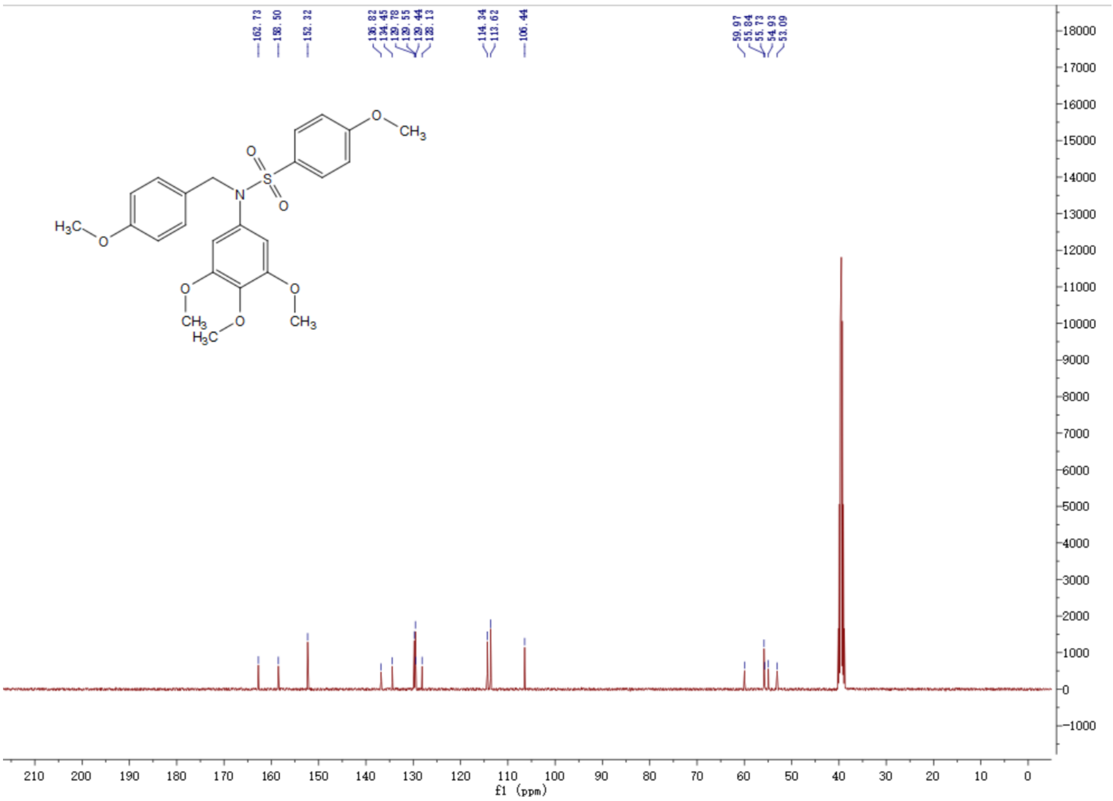

Figure S12. ^{13}C NMR spectrum of compound **6e** (100 MHz, DMSO-d_6)

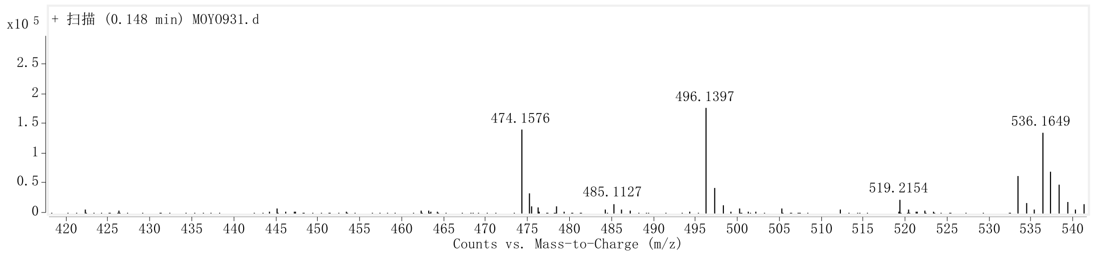

Figure S13. HRMS spectrum of compound **6e**

- ^{1}H, ^{13}C-NMR and HRMS of Compound **6f**

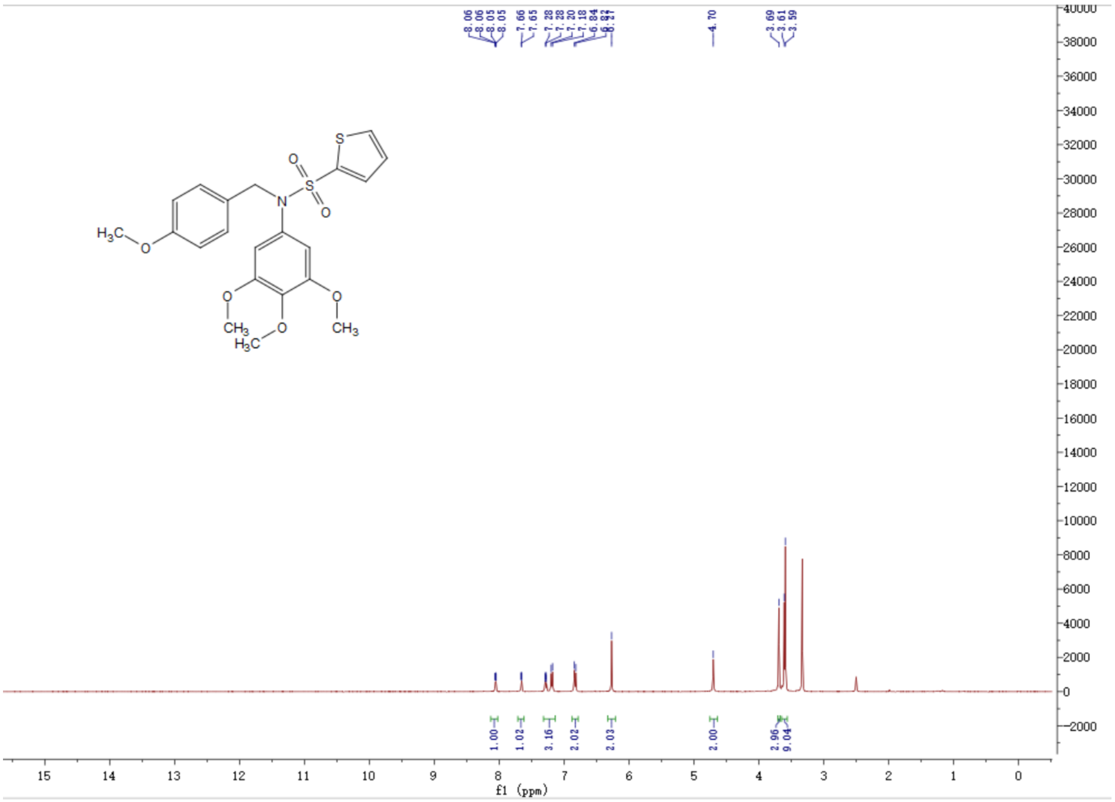

Figure S14. ^{1}H NMR spectrum of compound **6f** (400 MHz, DMSO-d_6)

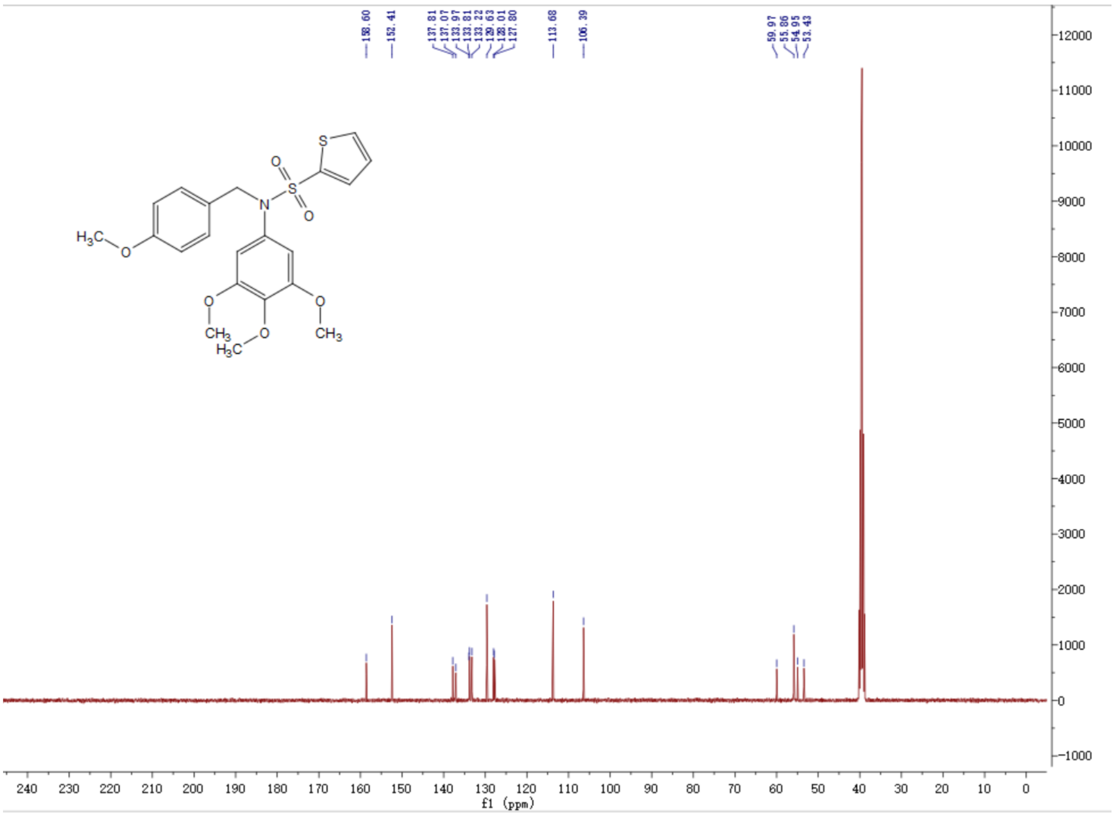

Figure S15. ^{13}C NMR spectrum of compound **6f** (100 MHz, DMSO-d_6)

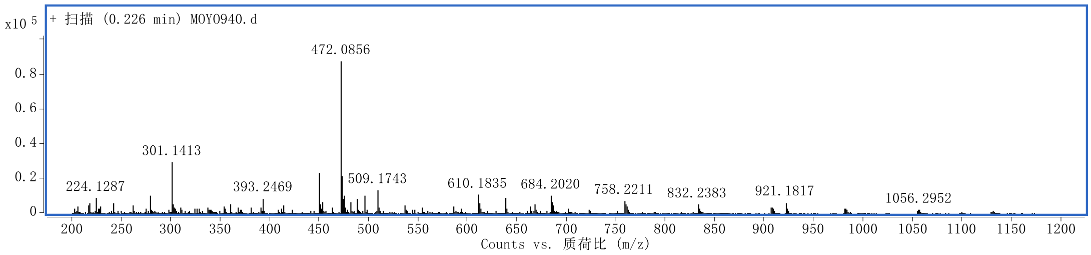

Figure S16. HRMS spectrum of compound **6f**

- ^{1}H, ^{13}C-NMR and HRMS of Compound **6g**

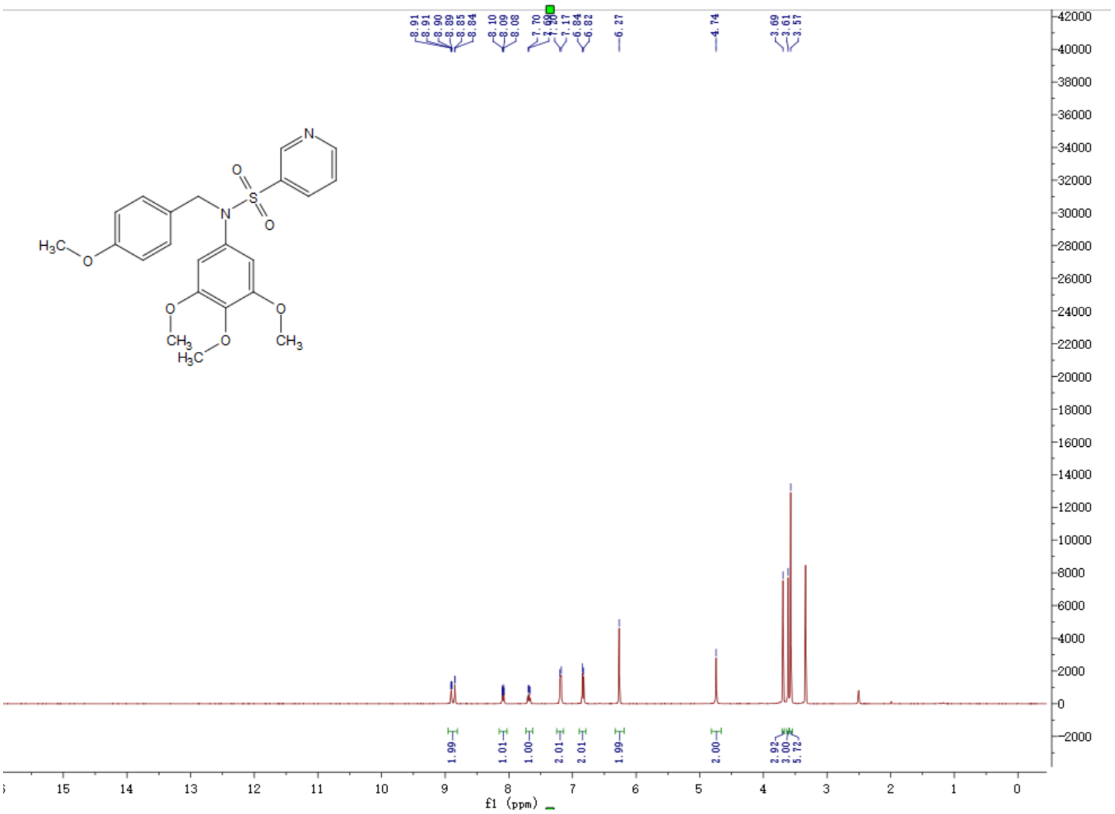

Figure S17. ^{1}H NMR spectrum of compound **6g** (400 MHz, DMSO-*d*6)

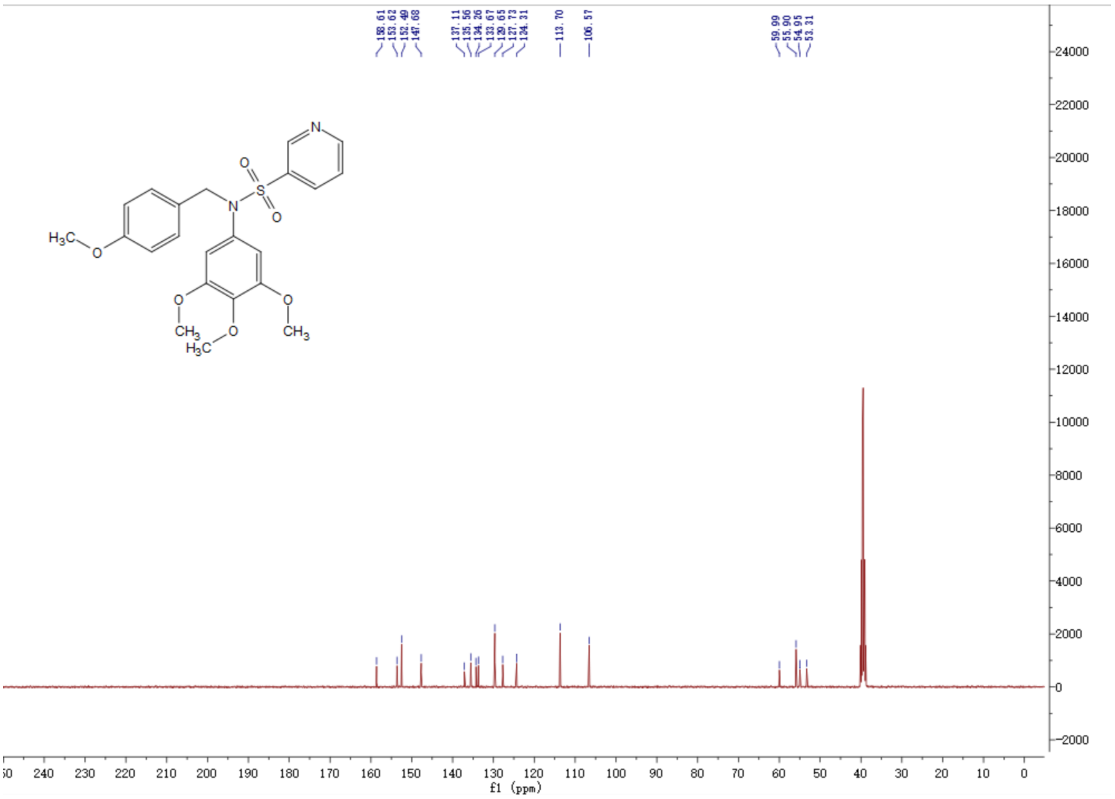

Figure S18. ^{13}C NMR spectrum of compound **6g** (100 MHz, DMSO-*d*6)

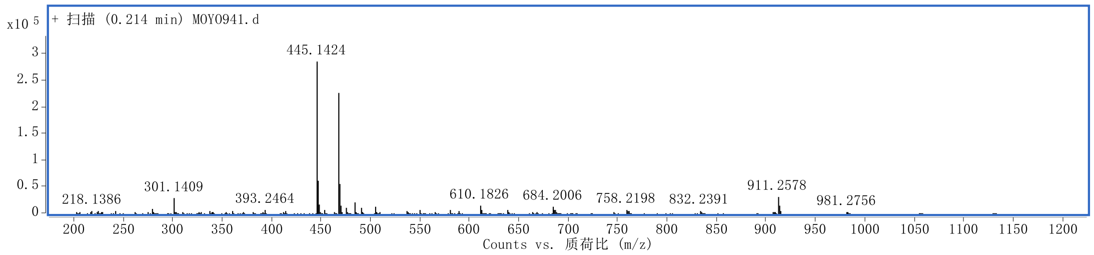

Figure S19. HRMS spectrum of compound **6g**

- ^{1}H, ^{13}C-NMR and HRMS of Compound **9a**

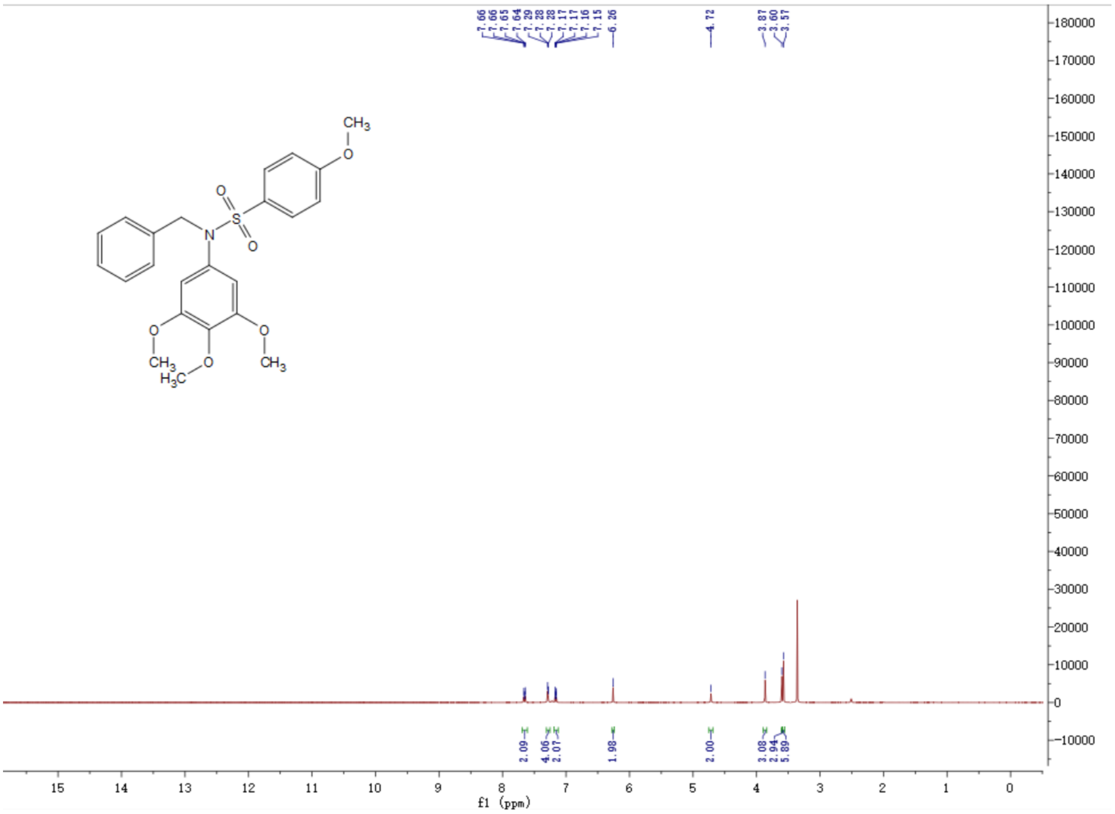

Figure S20. ^{1}H NMR spectrum of compound **9a** (400 MHz, DMSO-*d6*)

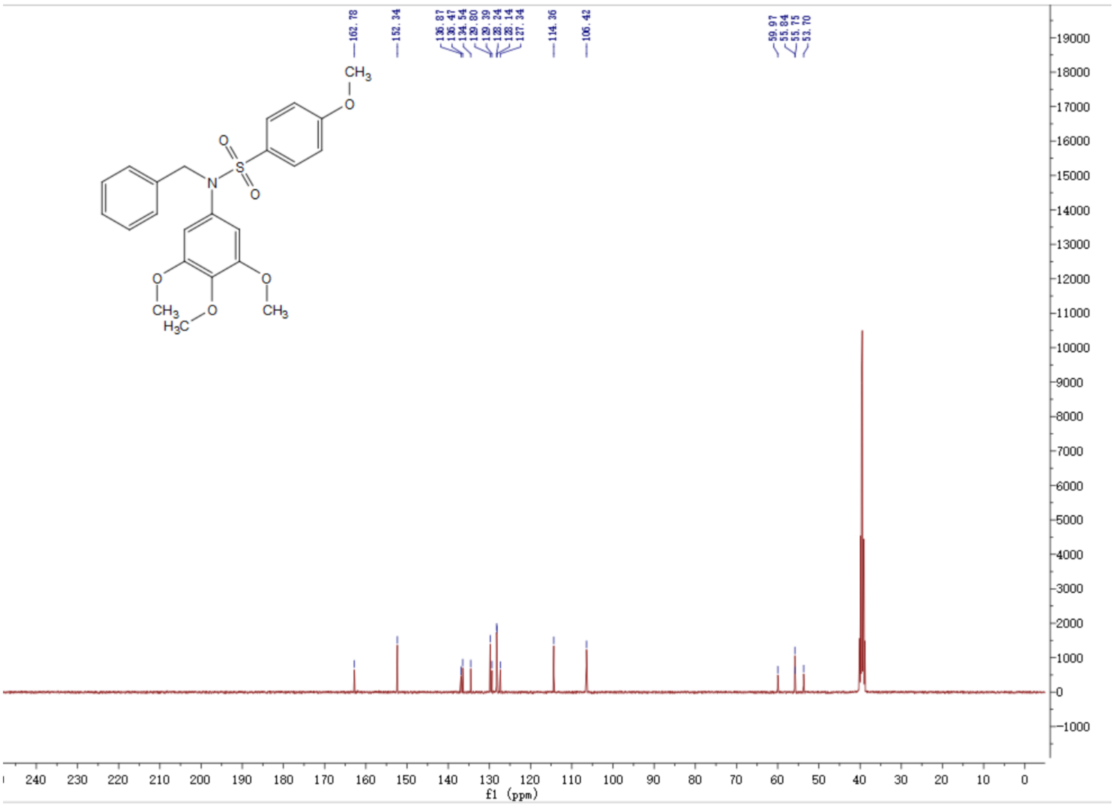

Figure S21. ^{13}C NMR spectrum of compound **9a** (100 MHz, DMSO-*d6*)

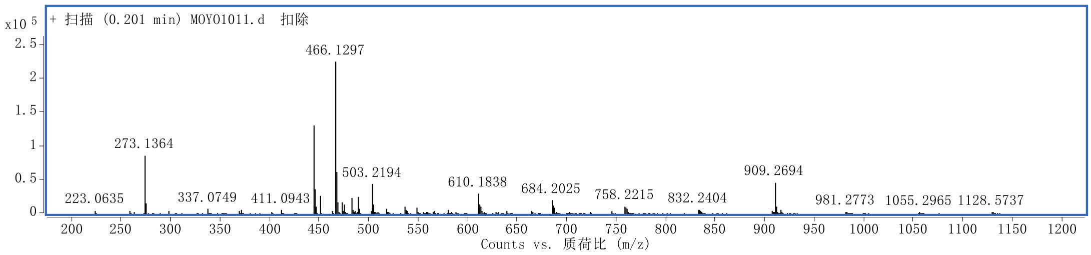

Figure S22. HRMS spectrum of compound **9a**

- ^{1}H, ^{13}C-NMR and HRMS of Compound **9b**

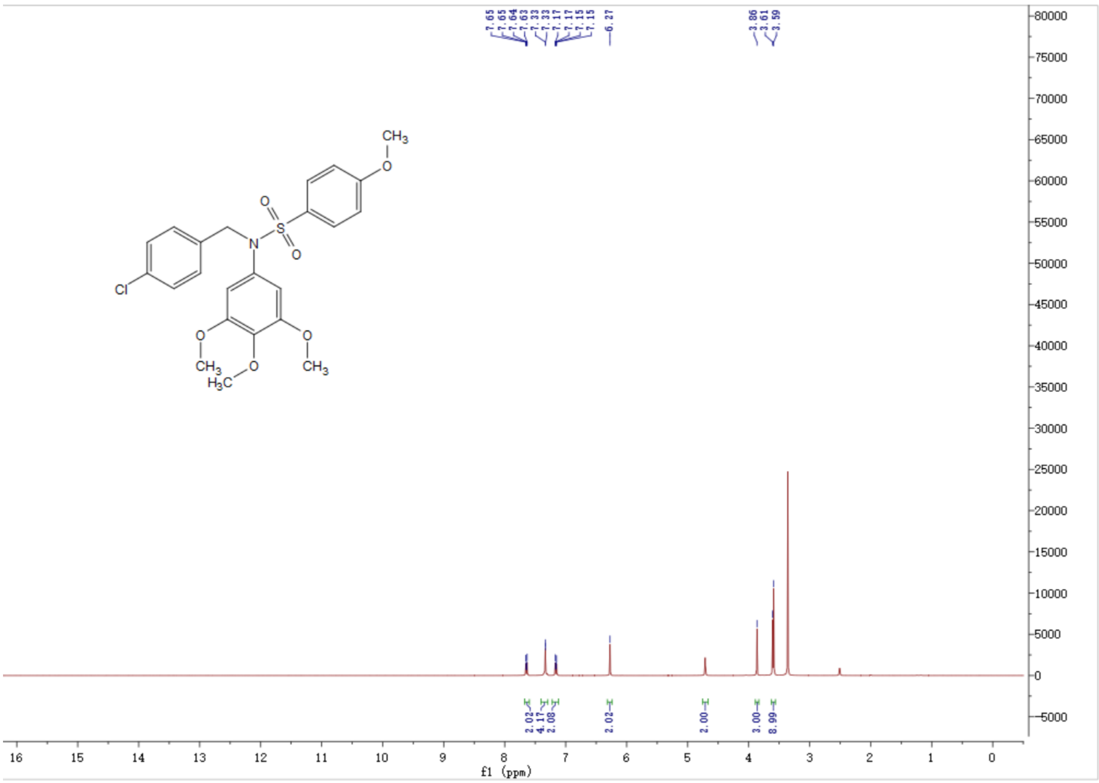

Figure S23. ^{13}C NMR spectrum of compound **9b** (100 MHz, DMSO-*d6*)

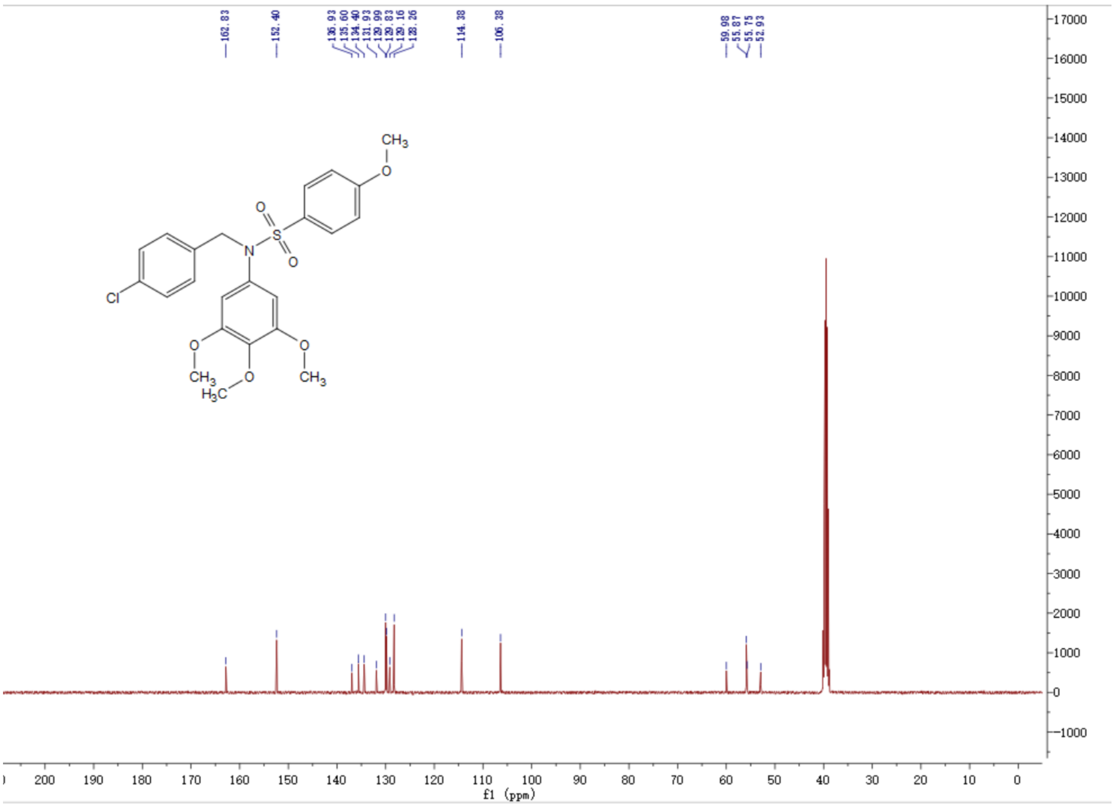

Figure S24. ^{1}H NMR spectrum of compound **9b** (400 MHz, DMSO-*d6*)

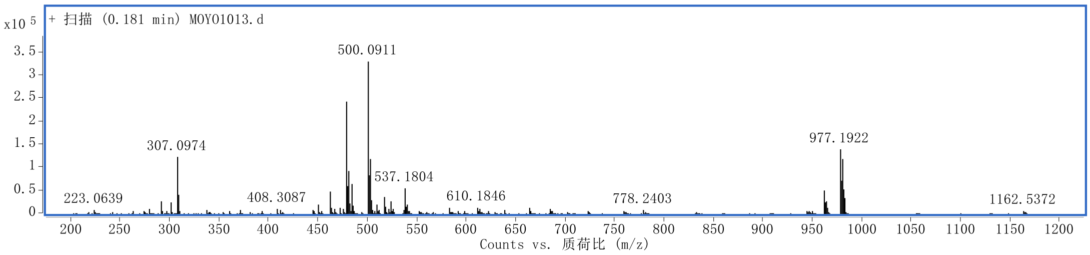

Figure S25. HRMS spectrum of compound **9b**

- ^{1}H, ^{13}C-NMR and HRMS of Compound **9c**

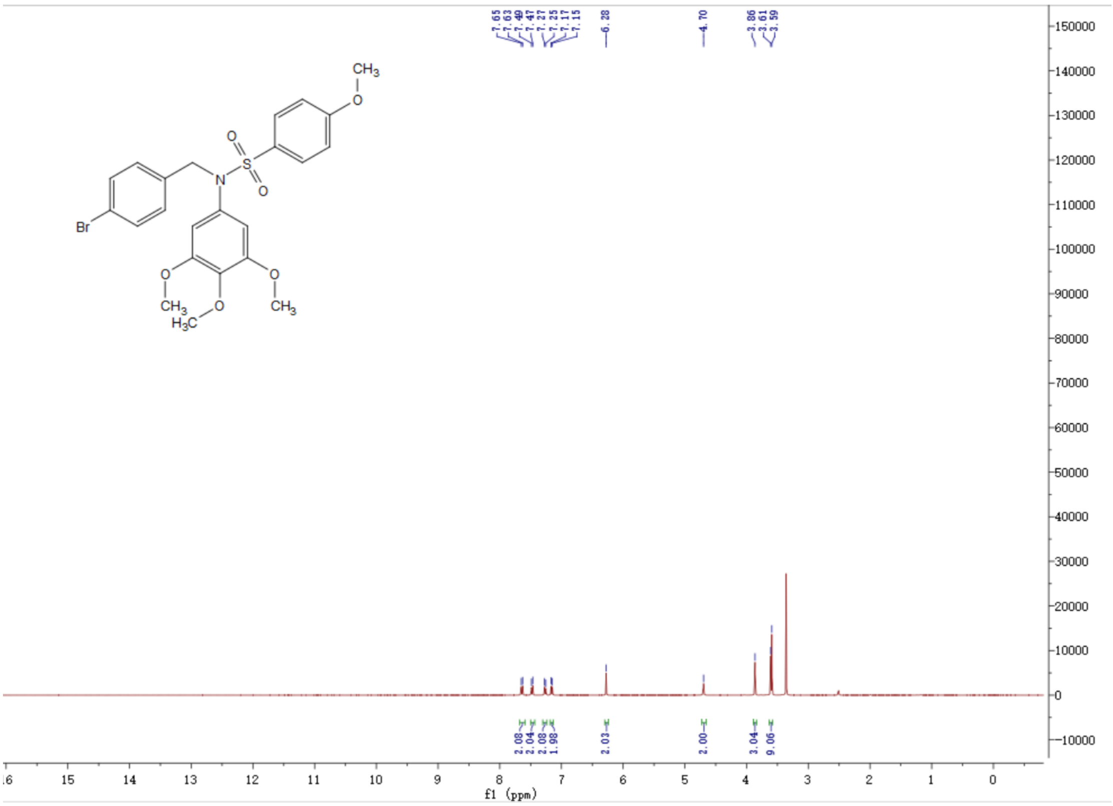

Figure S26. ^{1}H NMR spectrum of compound **9c** (400 MHz, DMSO-d_6)

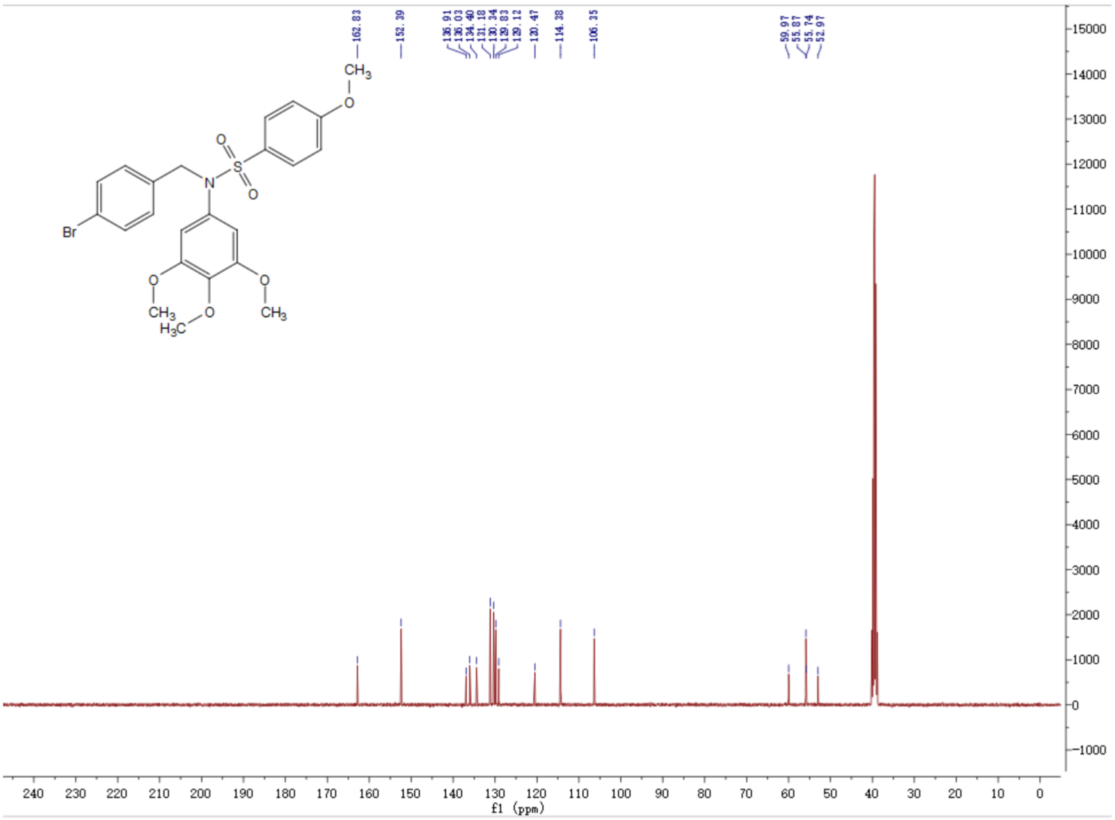

Figure S27. ^{13}C NMR spectrum of compound **9c** (100 MHz, DMSO-*d6*)

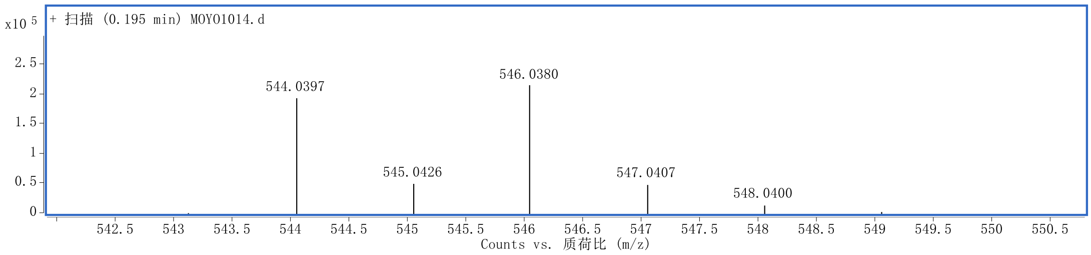

Figure S28. HRMS spectrum of compound **9c**

- ^{1}H, ^{13}C-NMR and HRMS of Compound **9d**

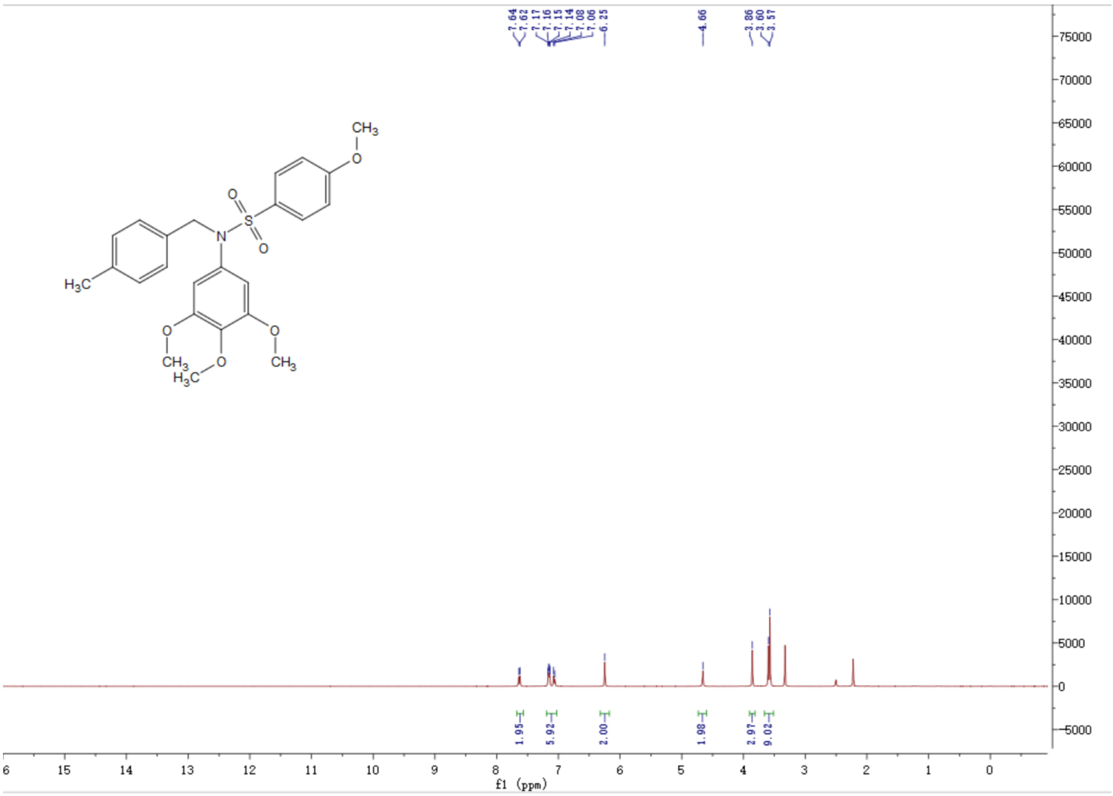

Figure S29. ^{1}H NMR spectrum of compound **9d** (400 MHz, DMSO-*d6*)

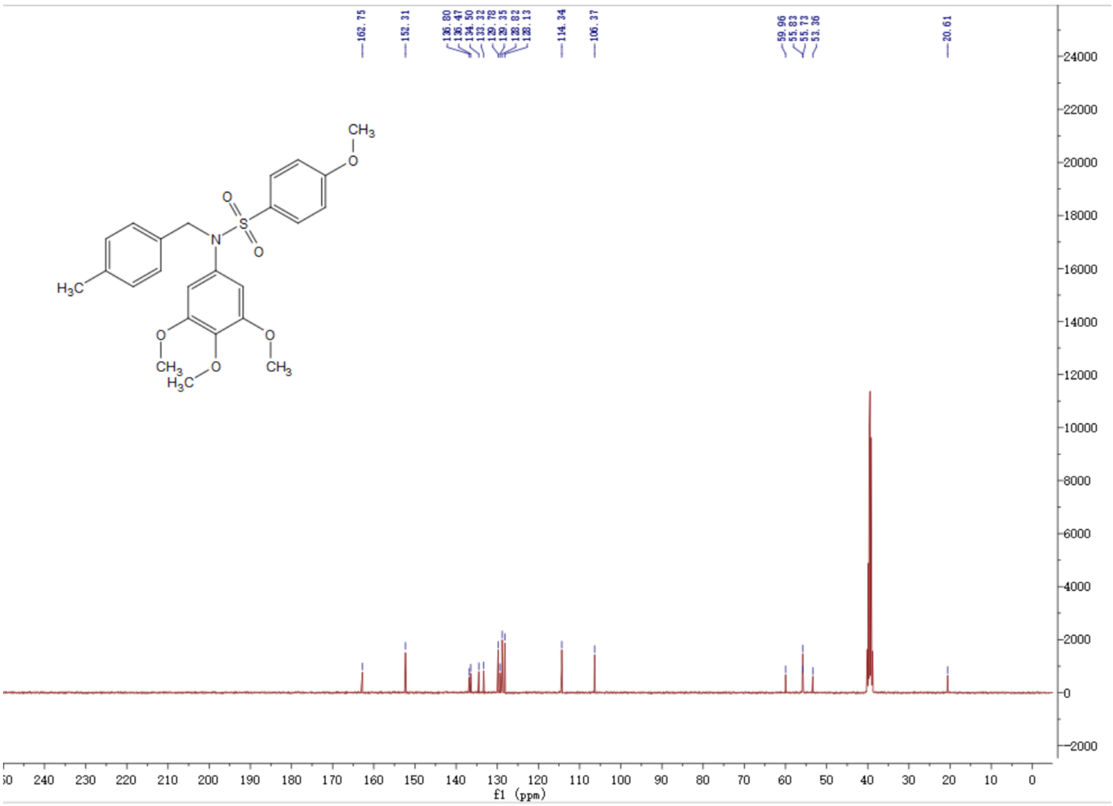

Figure S30. ^{13}C NMR spectrum of compound **9d** (100 MHz, DMSO-*d6*)

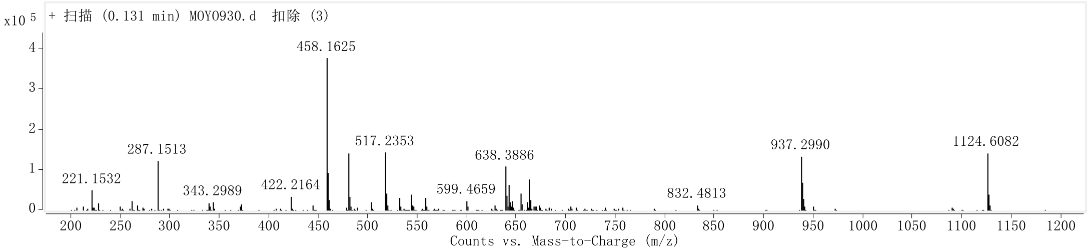

Figure S31. HRMS spectrum of compound **9d**

- ^{1}H, ^{13}C-NMR and HRMS of Compound **9e**

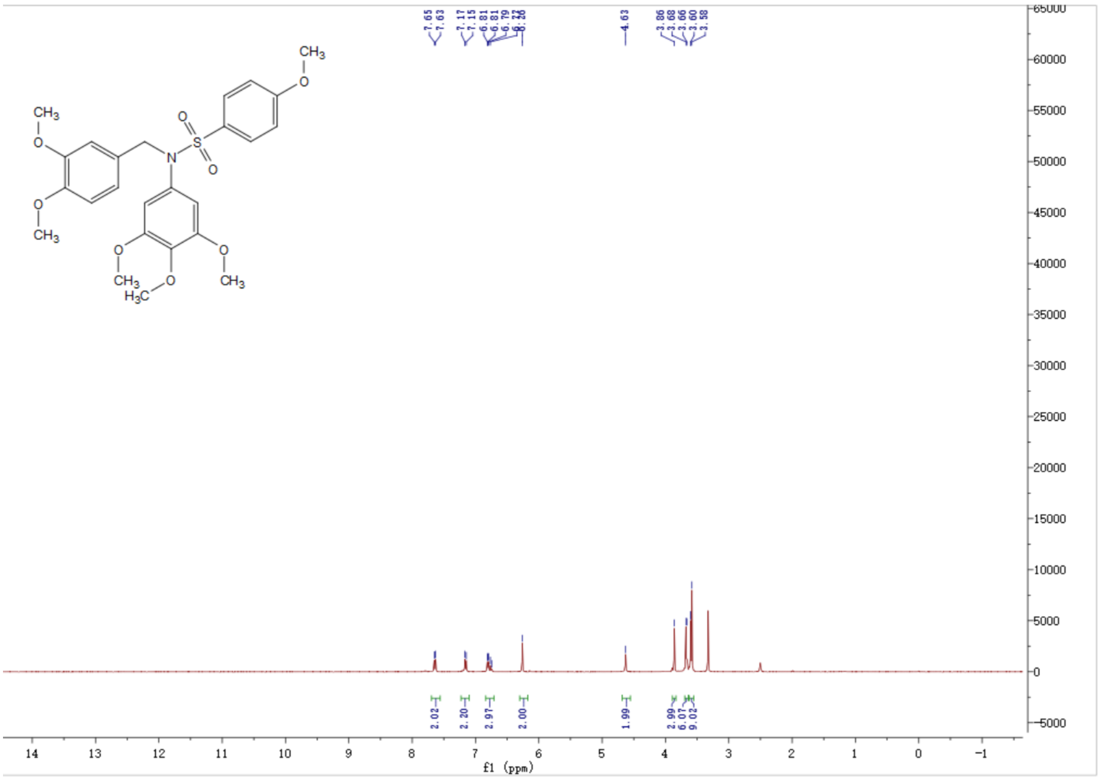

Figure S32. ^{13}C NMR spectrum of compound **9e** (100 MHz, DMSO-*d*6)

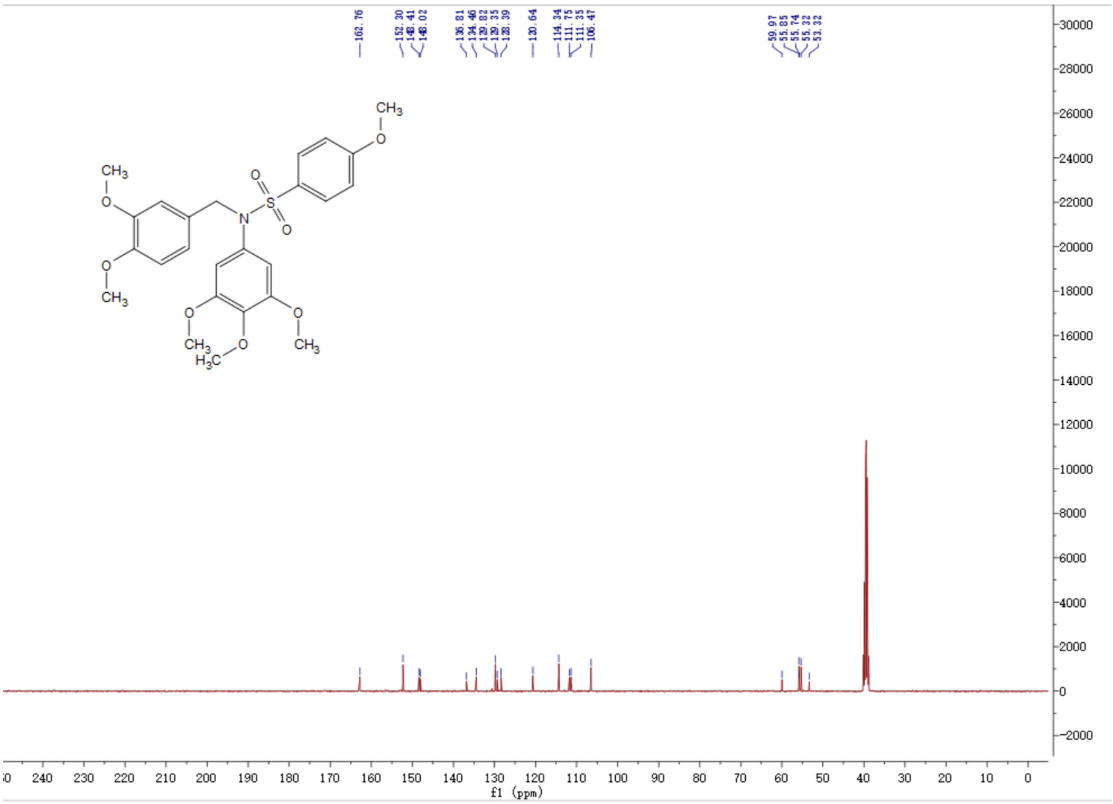

Figure S33. ^{1}H NMR spectrum of compound **9e** (400 MHz, DMSO-d_6)

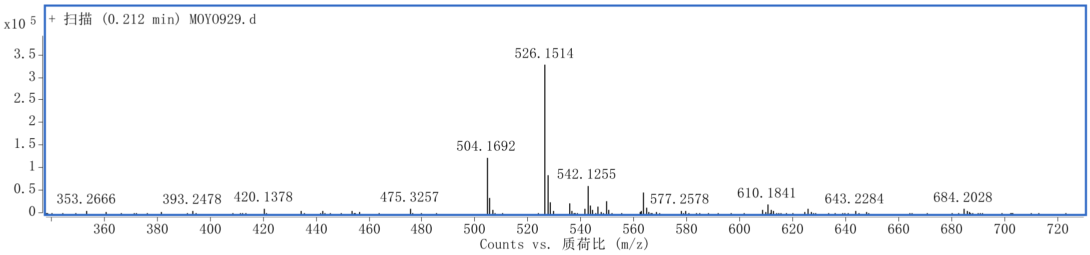

Figure S34. HRMS spectrum of compound **9e**

- ^{1}H, ^{13}C-NMR and HRMS of Compound **9f**

9h

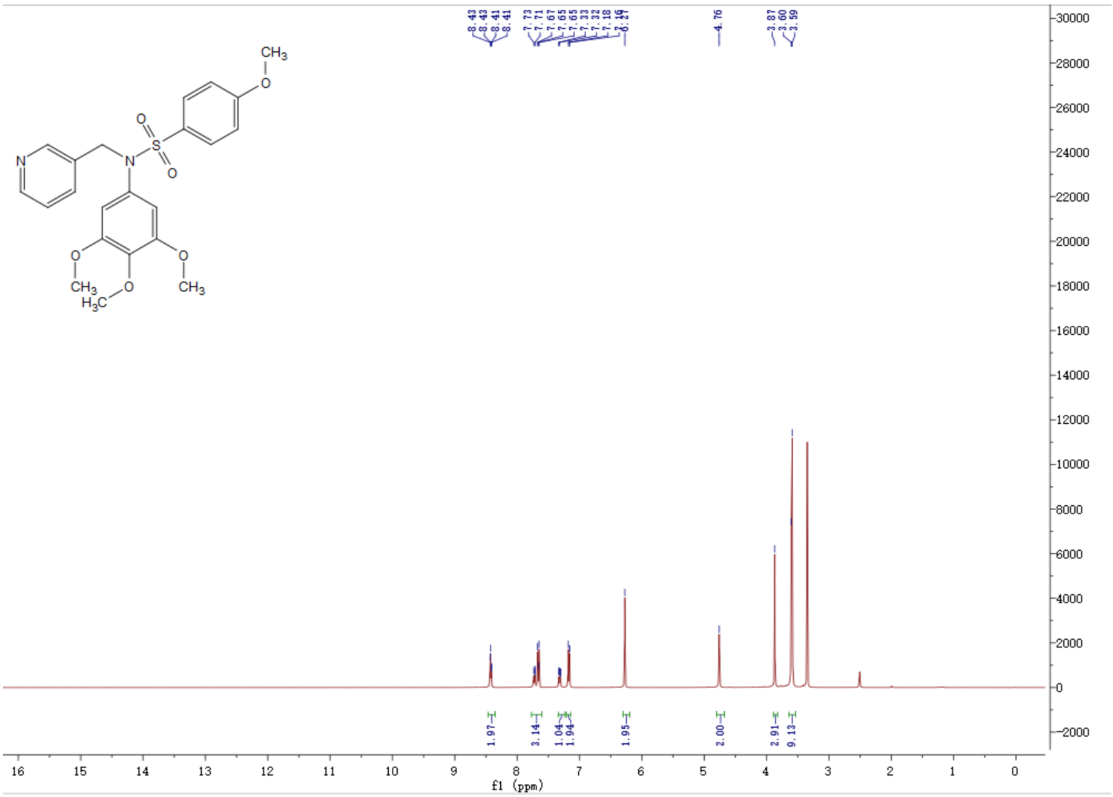

Figure S35. ^{1}H NMR spectrum of compound **9f** (400 MHz, DMSO-*d6*)

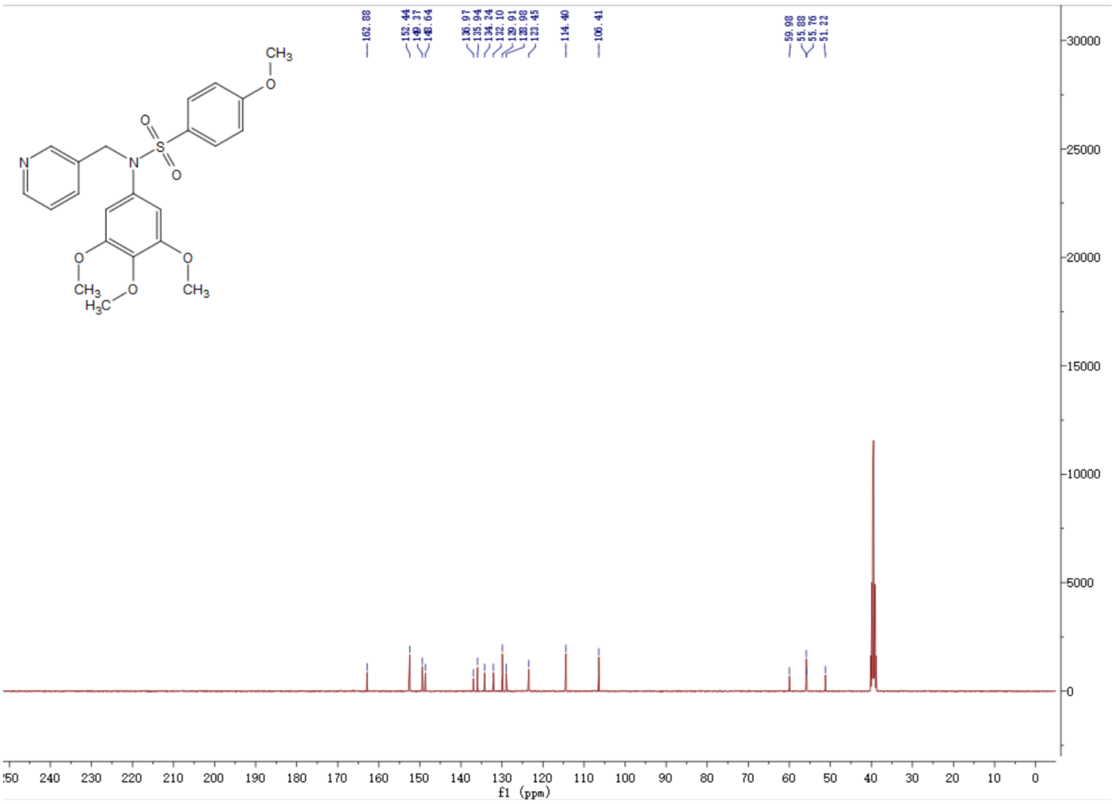

Figure S36. ^{13}C NMR spectrum of compound **9f** (100 MHz, DMSO-*d6*)

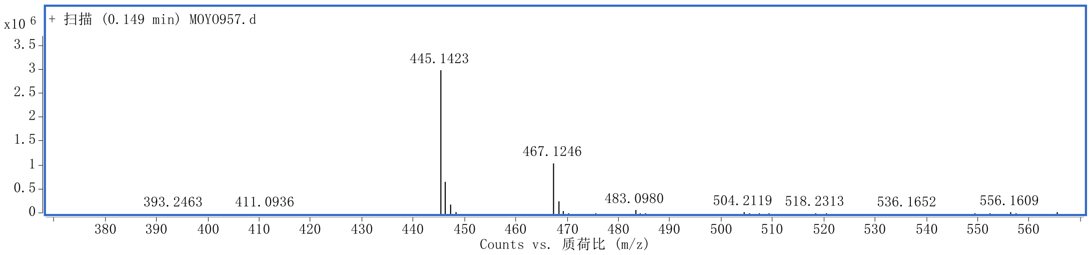

Figure S37. HRMS spectrum of compound **9f**

- ^{1}H, ^{13}C-NMR and HRMS of Compound **9g**

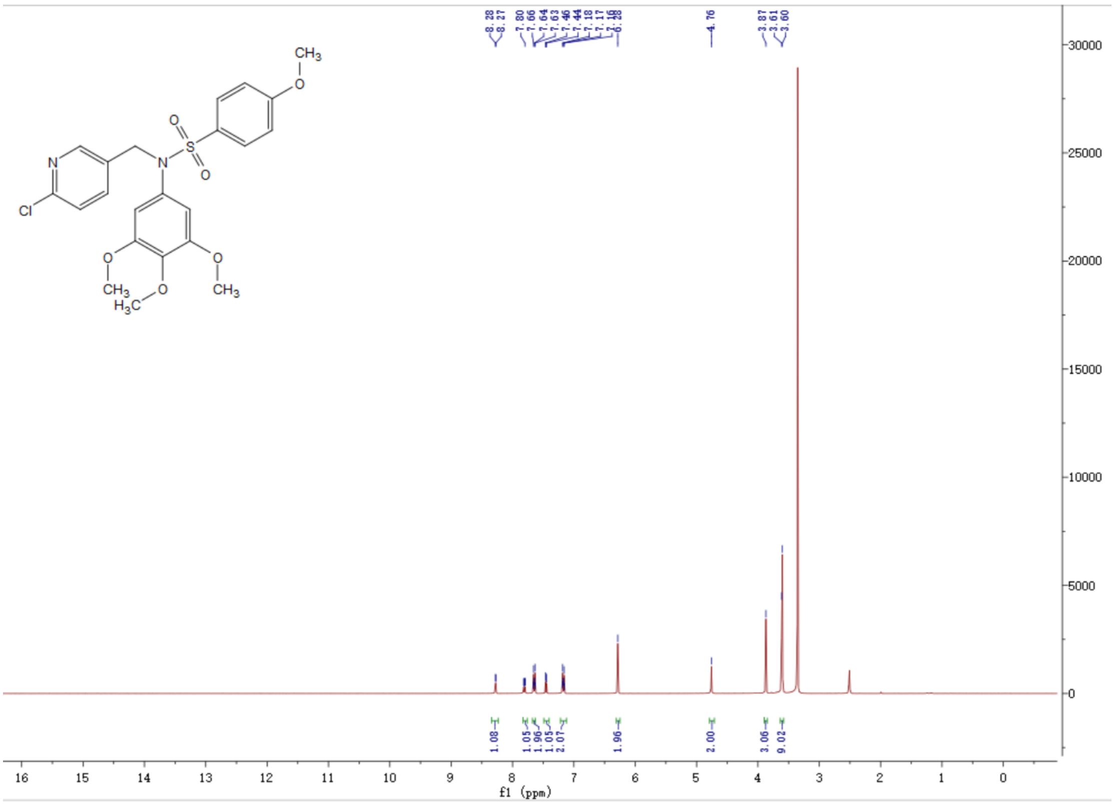

Figure S38. ^{13}C NMR spectrum of compound **9g** (100 MHz, DMSO-*d6*)

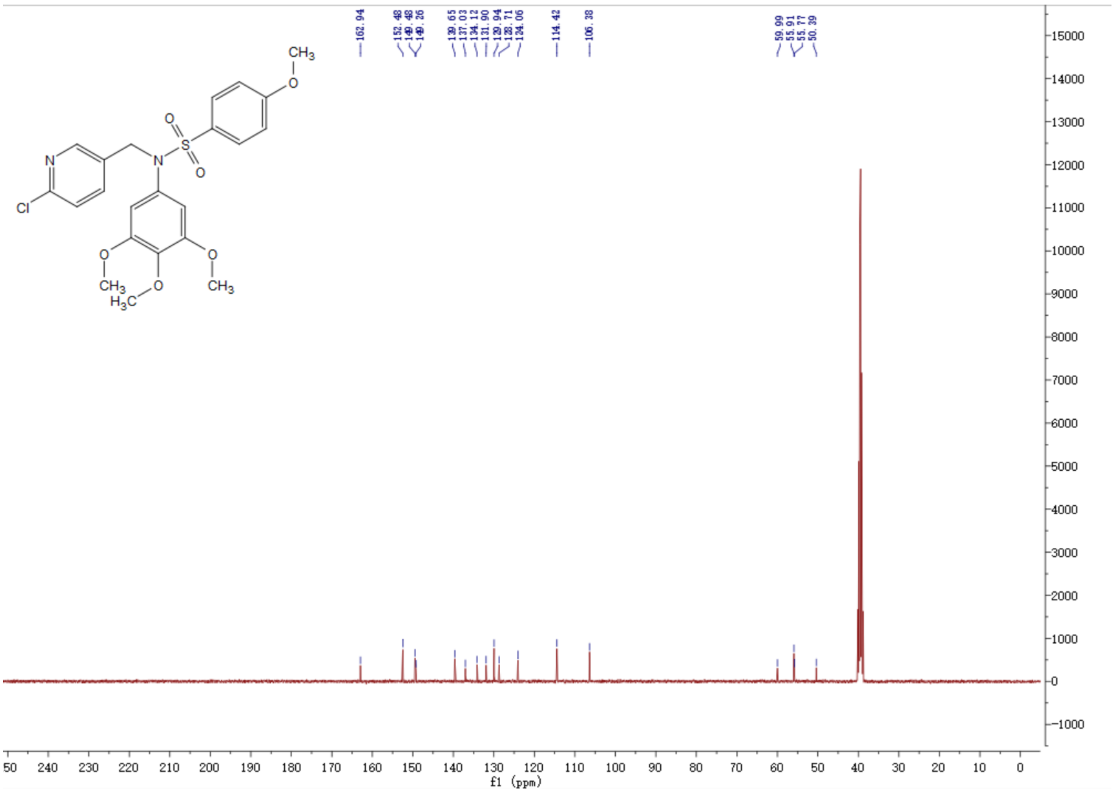

Figure S39. ^{1}H NMR spectrum of compound **9g** (400 MHz, DMSO-d_6)

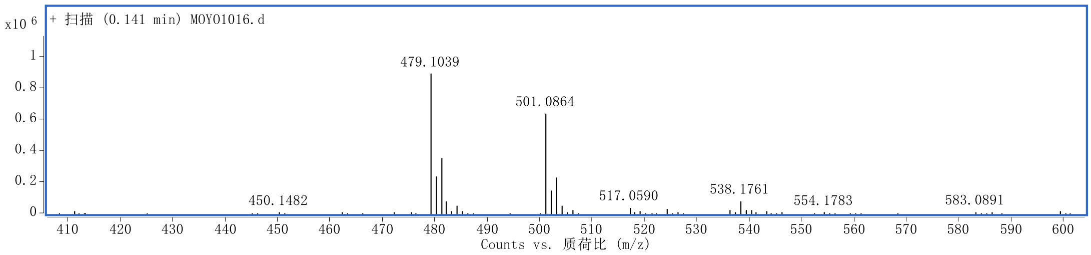

Figure S40. HRMS spectrum of compound **9g**

- ^{1}H, ^{13}C-NMR and HRMS of Compound **9h**

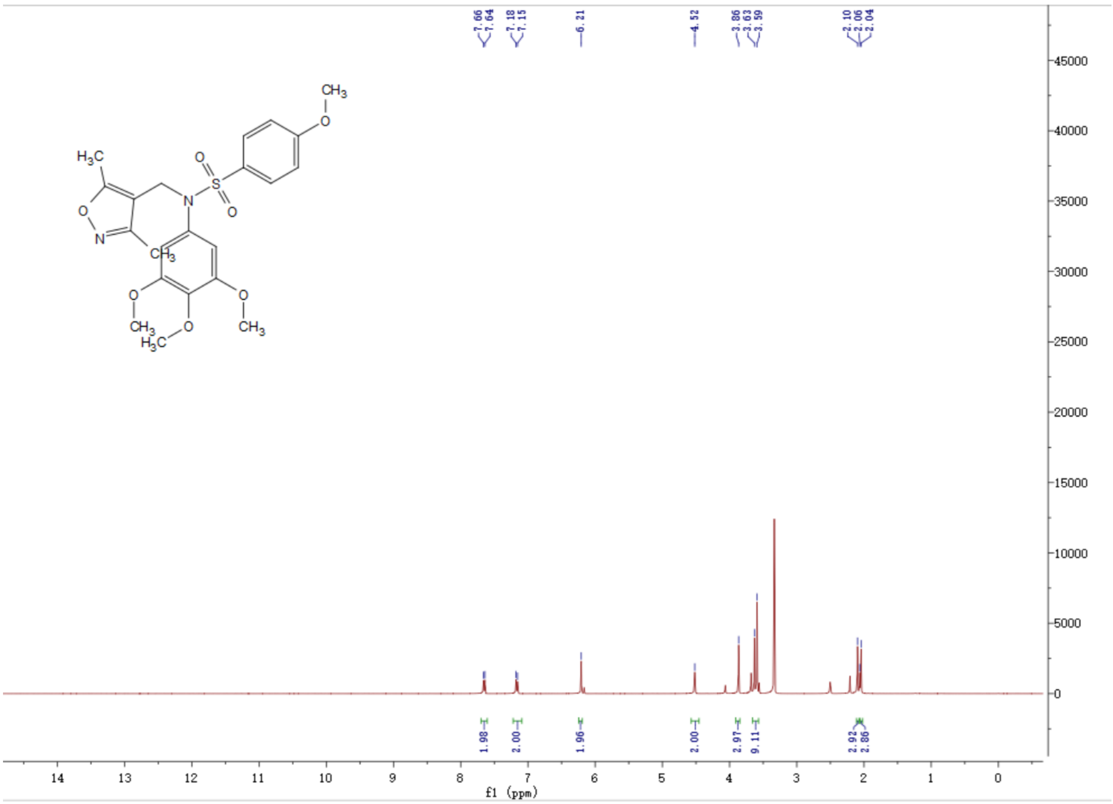

Figure S41. ^{1}H NMR spectrum of compound **9h** (400 MHz, DMSO-d_6)

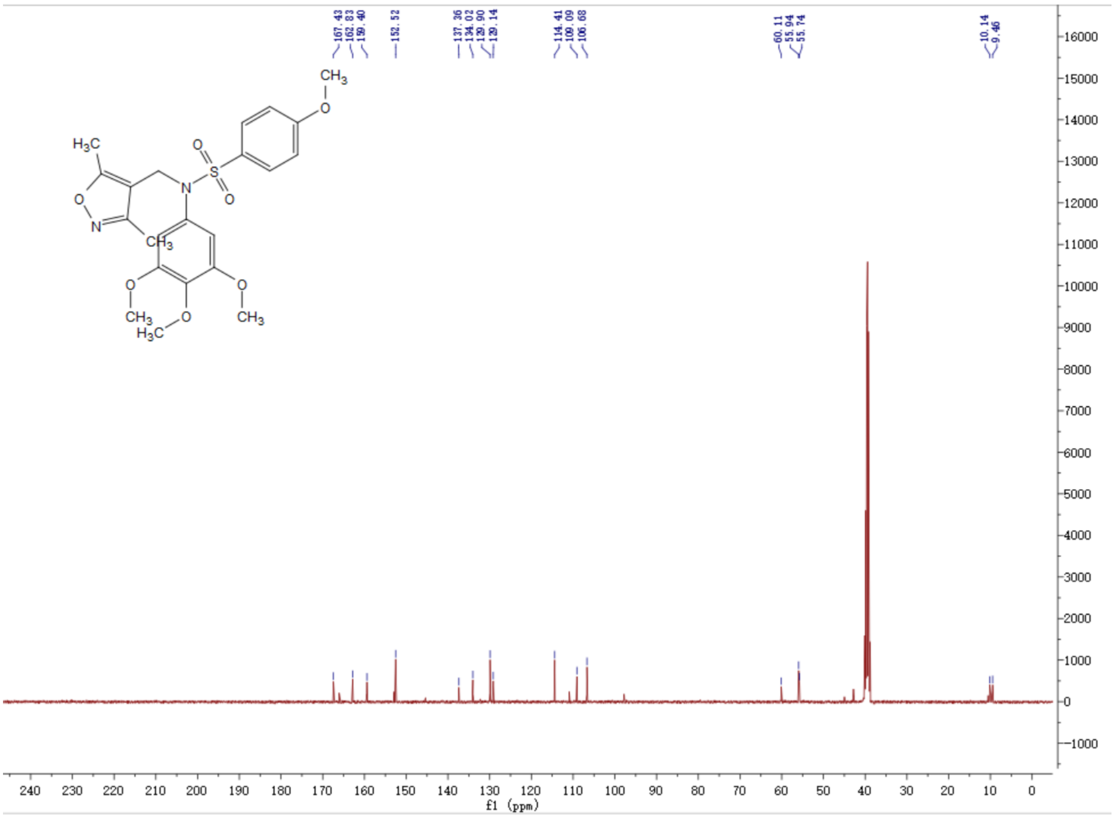

Figure S42. ^{13}C NMR spectrum of compound **9h** (100 MHz, DMSO-d_6)

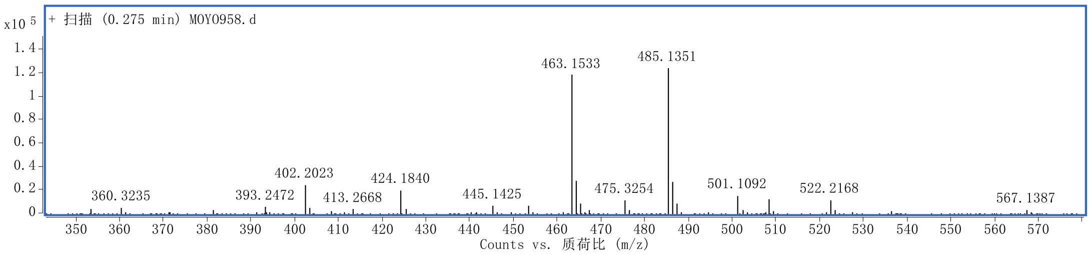

Figure S43. HRMS spectrum of compound **9h**

- ^{1}H, ^{13}C-NMR and HRMS of Compound **9i**

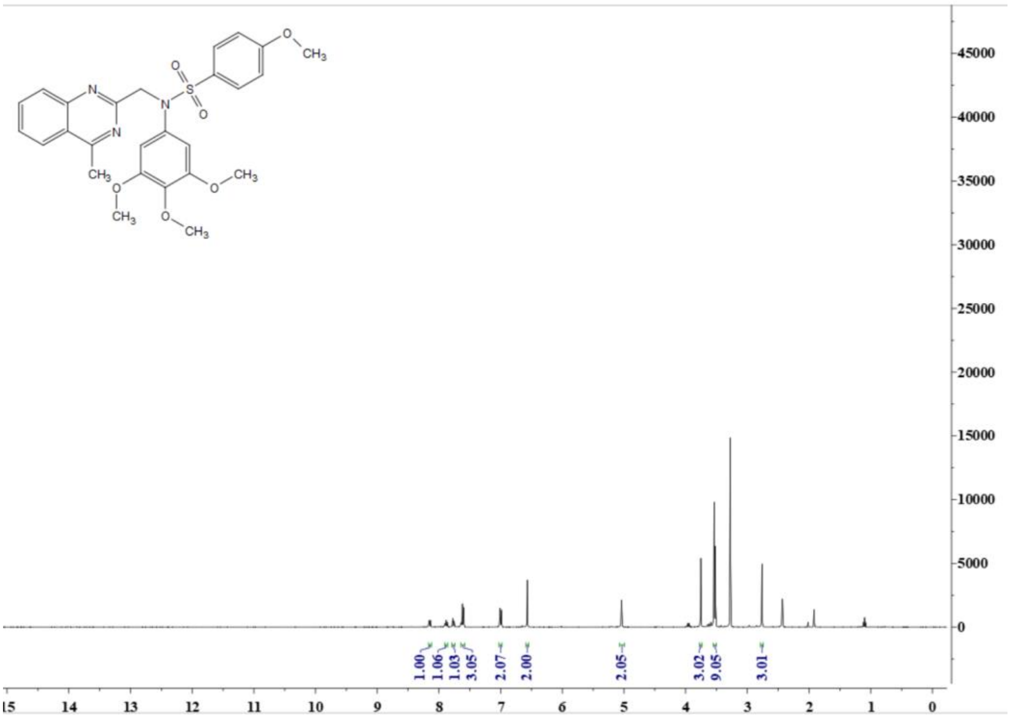

Figure S44. ^{1}H NMR spectrum of compound **9i**(400 MHz, DMSO-d_6)

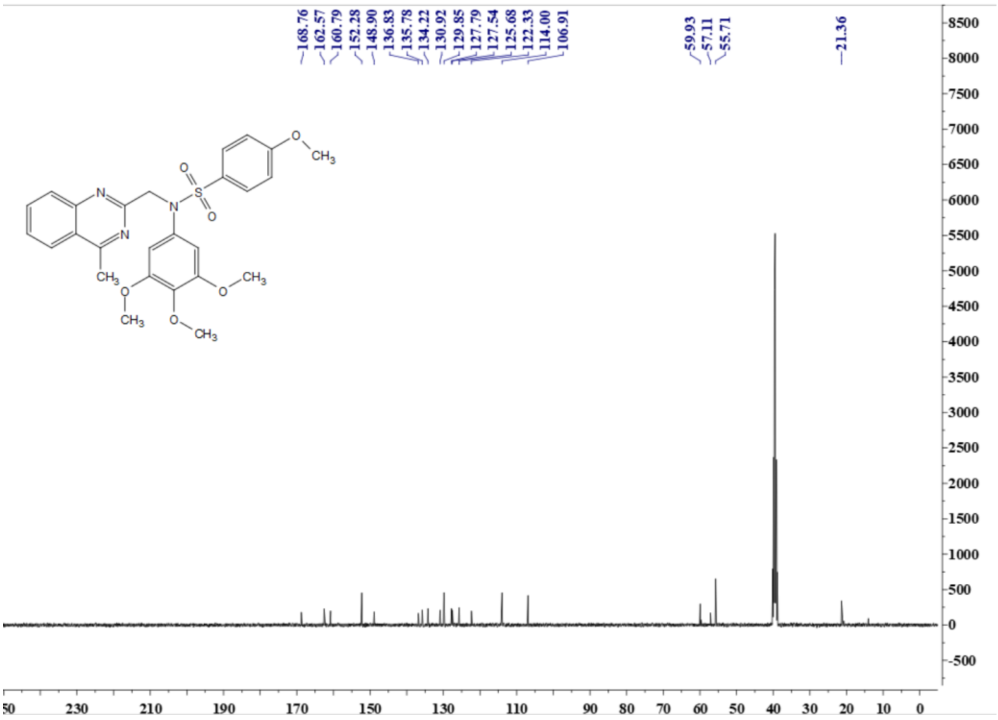

Figure S45. ^{13}C NMR spectrum of compound **9i**(100 MHz, DMSO-*d6*)

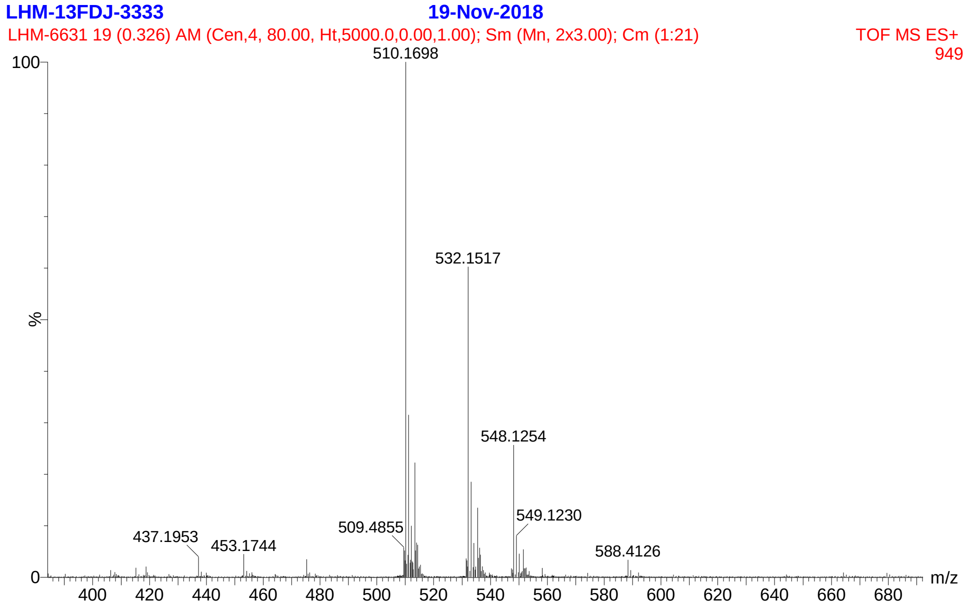

Figure S46. HRMS spectrum of compound **9i**

- ^{1}H, ^{13}C-NMR and HRMS of Compound **10a**

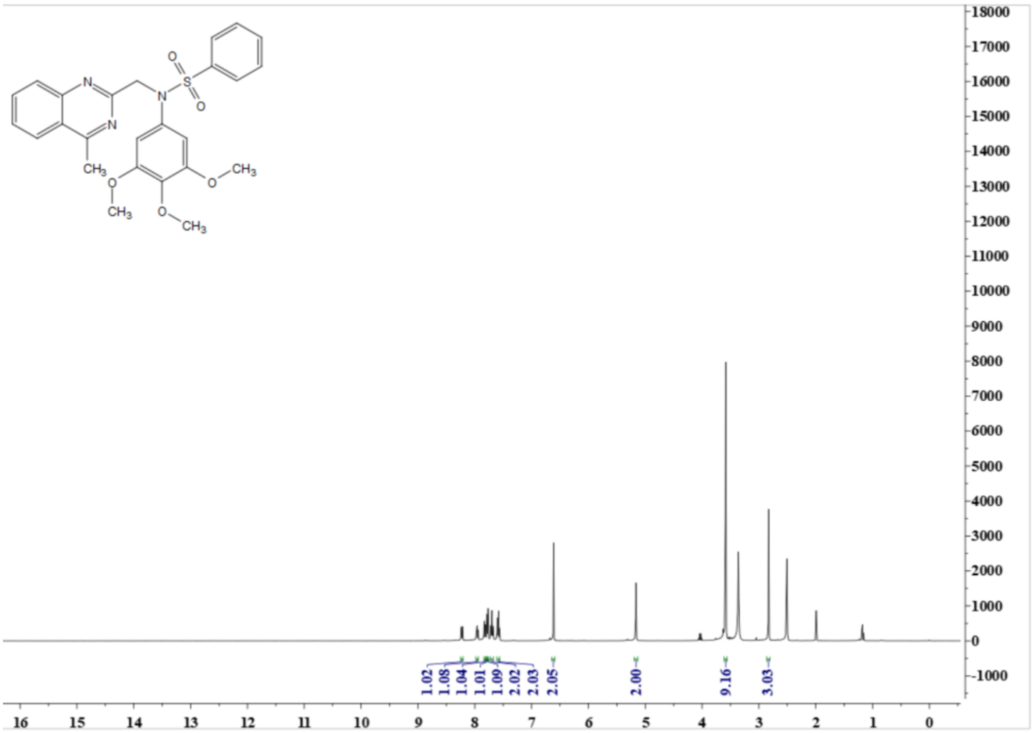

Figure S47. ^{1}H NMR spectrum of compound **10a** (400 MHz, DMSO-d_6)

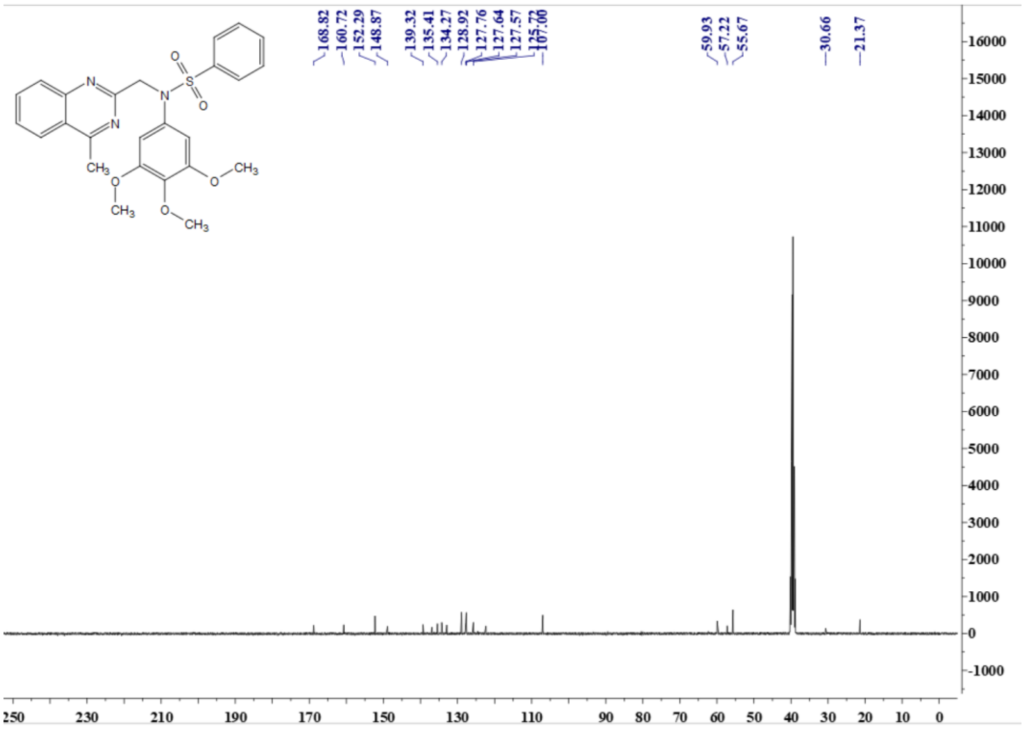

Figure S48. ^{13}C NMR spectrum of compound **10a** (100 MHz, DMSO-d_6)

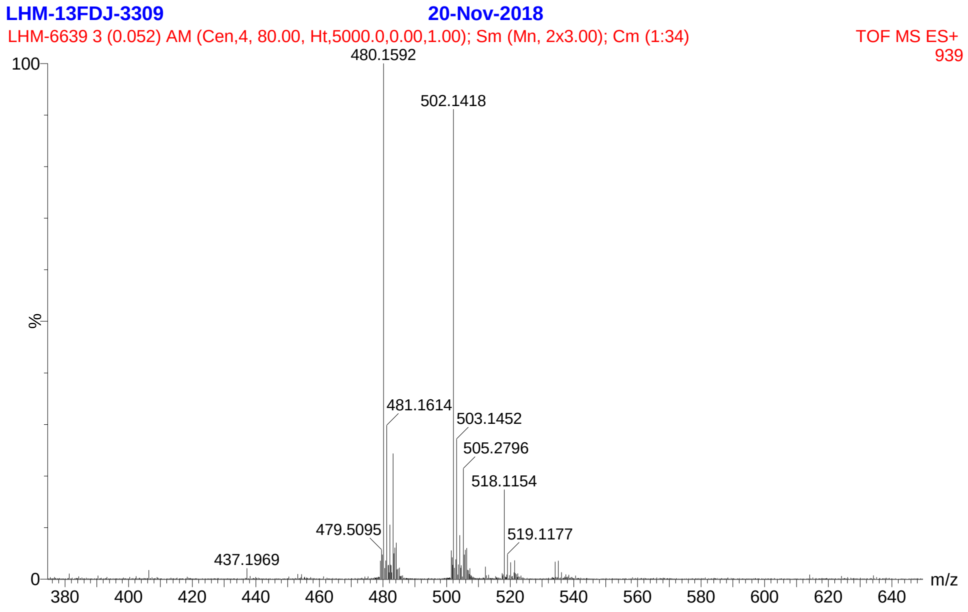

Figure S49. HRMS spectrum of compound **10a**

- ^{1}H, ^{13}C-NMR and HRMS of Compound **10b**

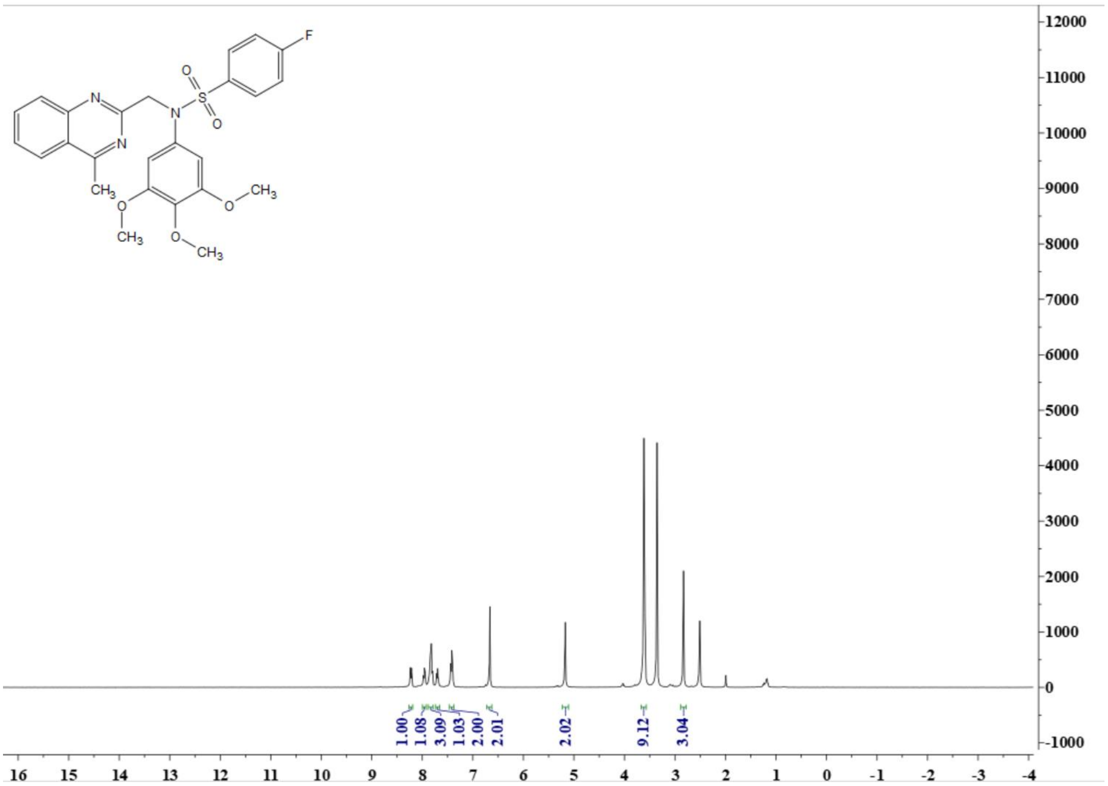

Figure S50. ^{1}H NMR spectrum of compound **10b** (400 MHz, DMSO-d_6)

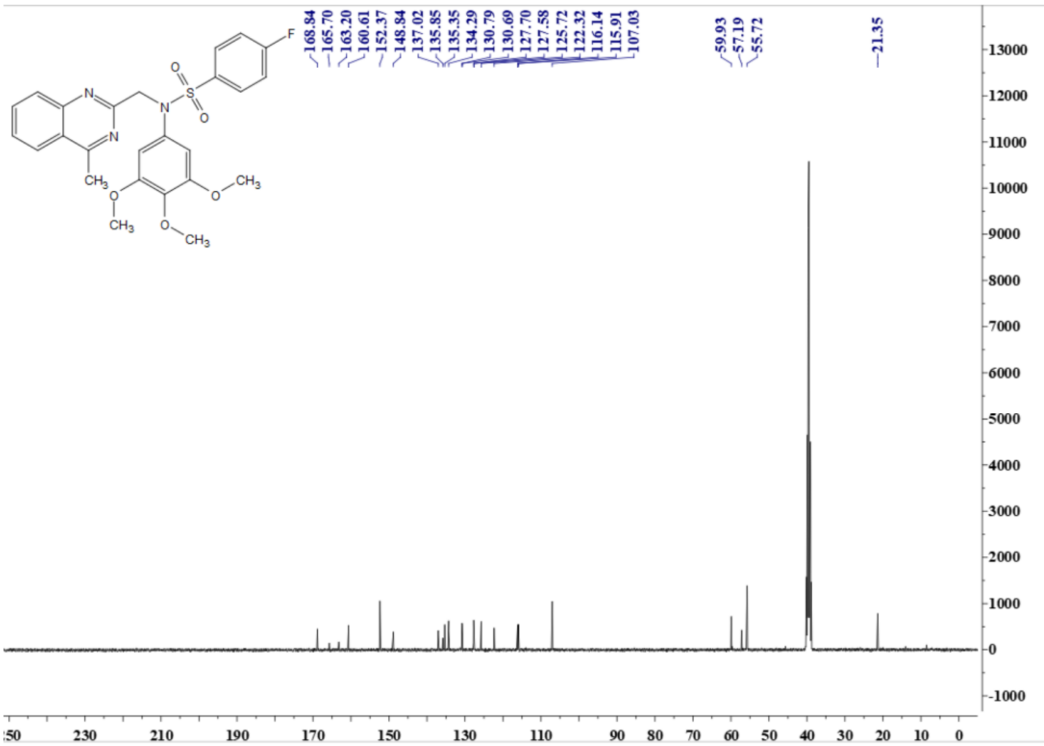

Figure S51. ^{13}C NMR spectrum of compound **10b** (100 MHz, DMSO-d_6)

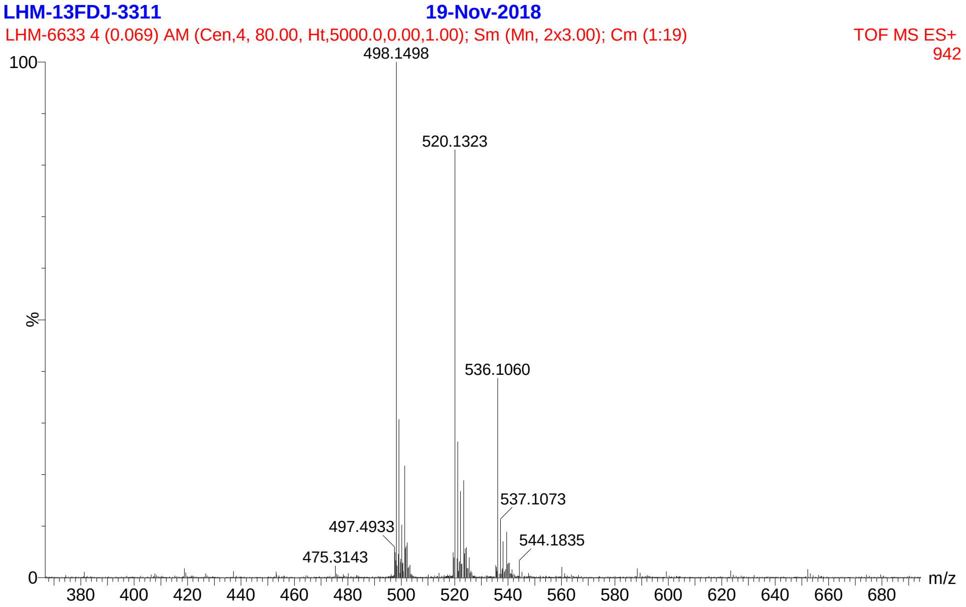

Figure S52. HRMS spectrum of compound **10b**

- ^{1}H, ^{13}C-NMR and HRMS of Compound **10c**

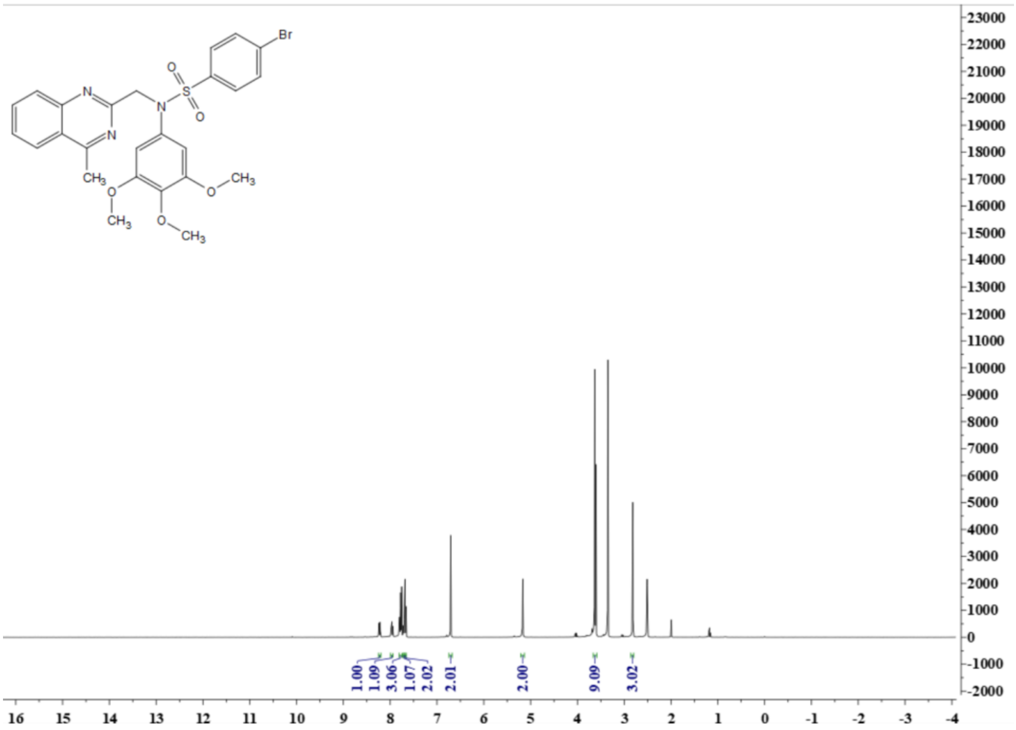

Figure S53. ^{1}H NMR spectrum of compound **10c** (400 MHz, DMSO-*d6*)

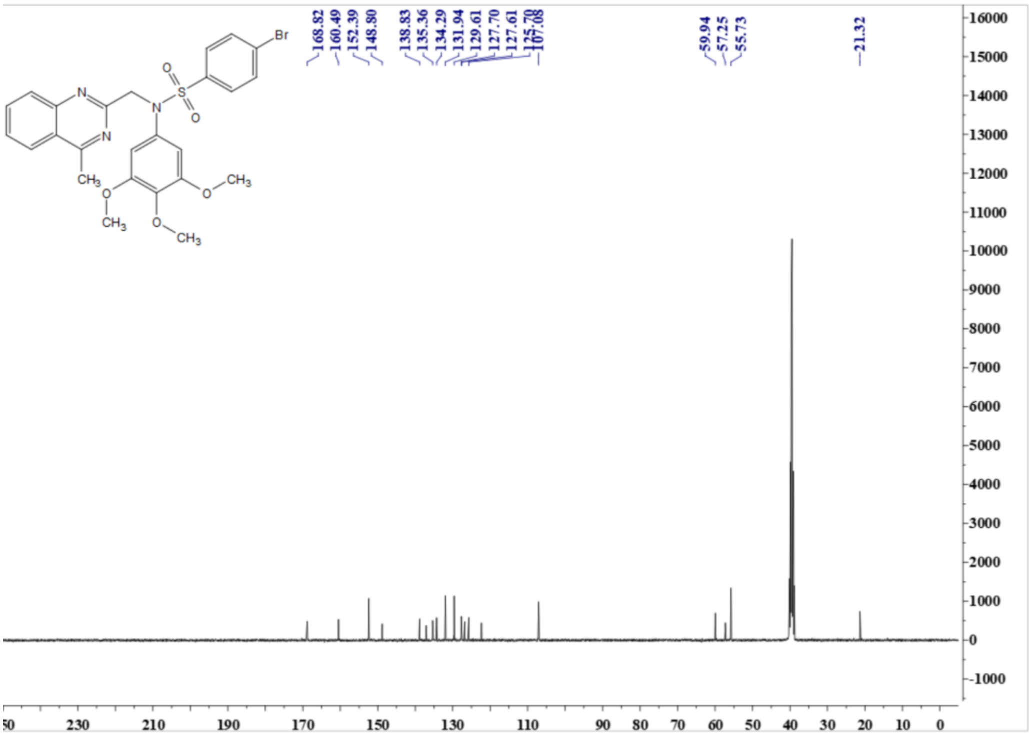

Figure S54. ^{13}C NMR spectrum of compound **10c** (100 MHz, DMSO-*d6*)

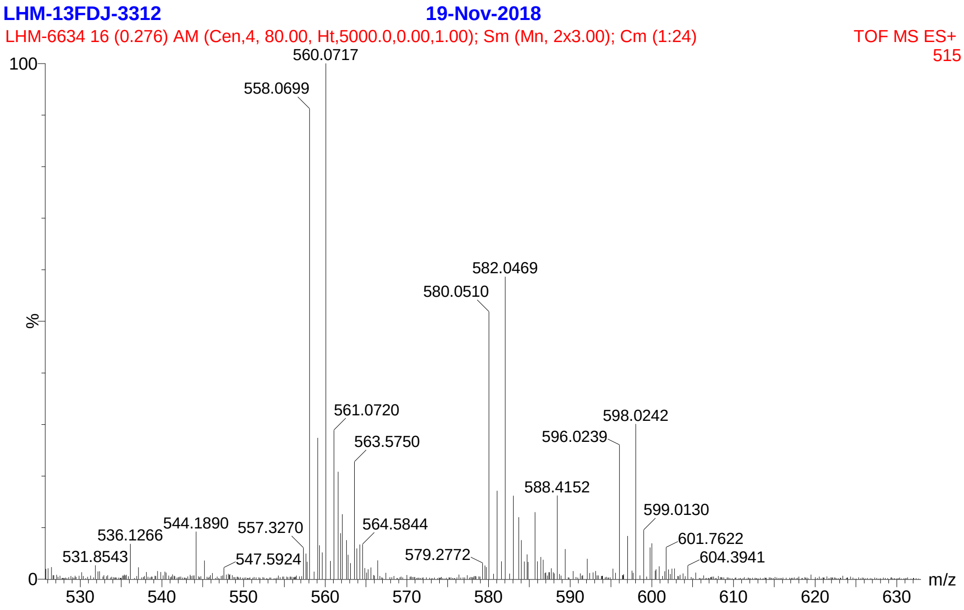

Figure S55. HRMS spectrum of compound **10c**

- ^{1}H, ^{13}C-NMR and HRMS of Compound **10d**

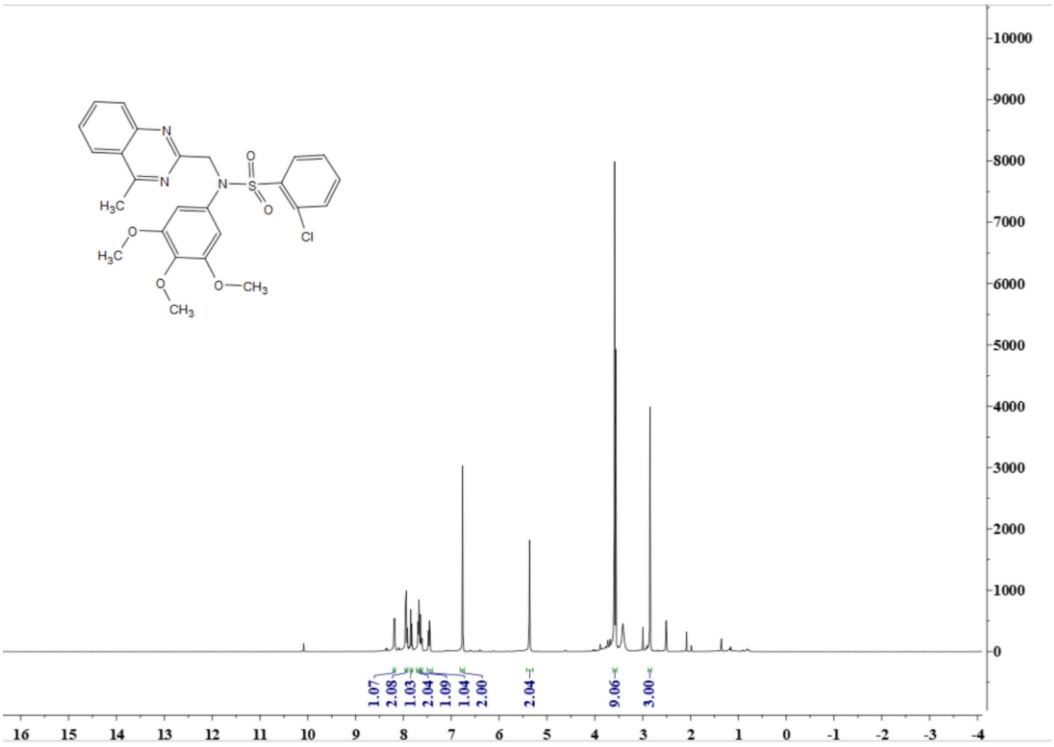

Figure S56. ^{1}H NMR spectrum of compound **10d** (400 MHz, DMSO-d_6)

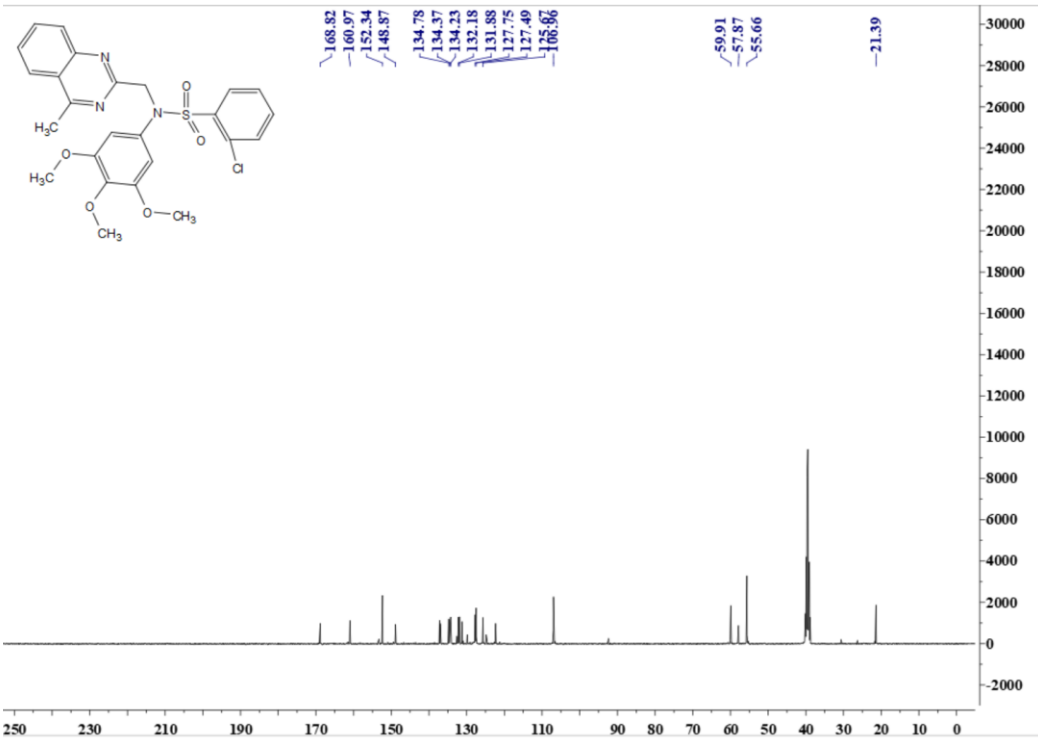

Figure S57. ^{13}C NMR spectrum of compound **10d** (100 MHz, DMSO-d_6)

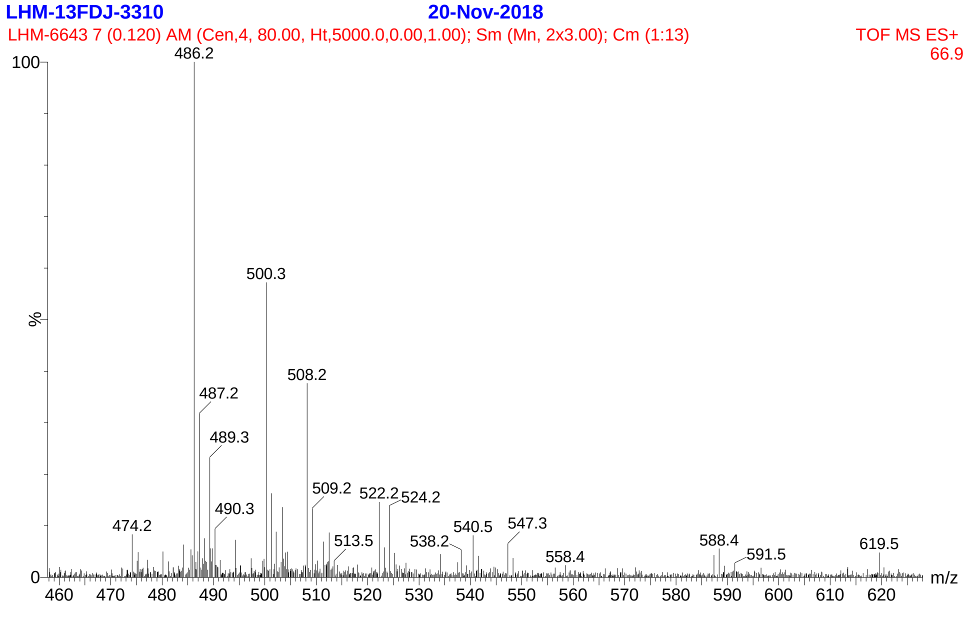

Figure S58. HRMS spectrum of compound **10d**

- ^{1}H, ^{13}C-NMR and HRMS of Compound **10e**

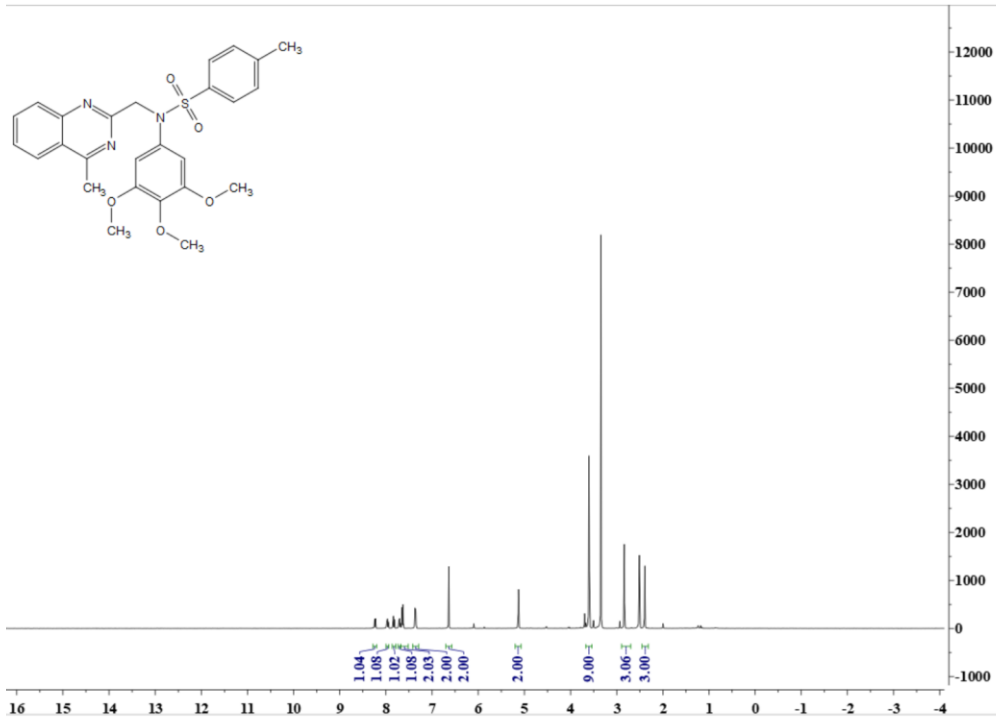

Figure S59. ^{1}H NMR spectrum of compound **10e** (400 MHz, DMSO-*d6*)

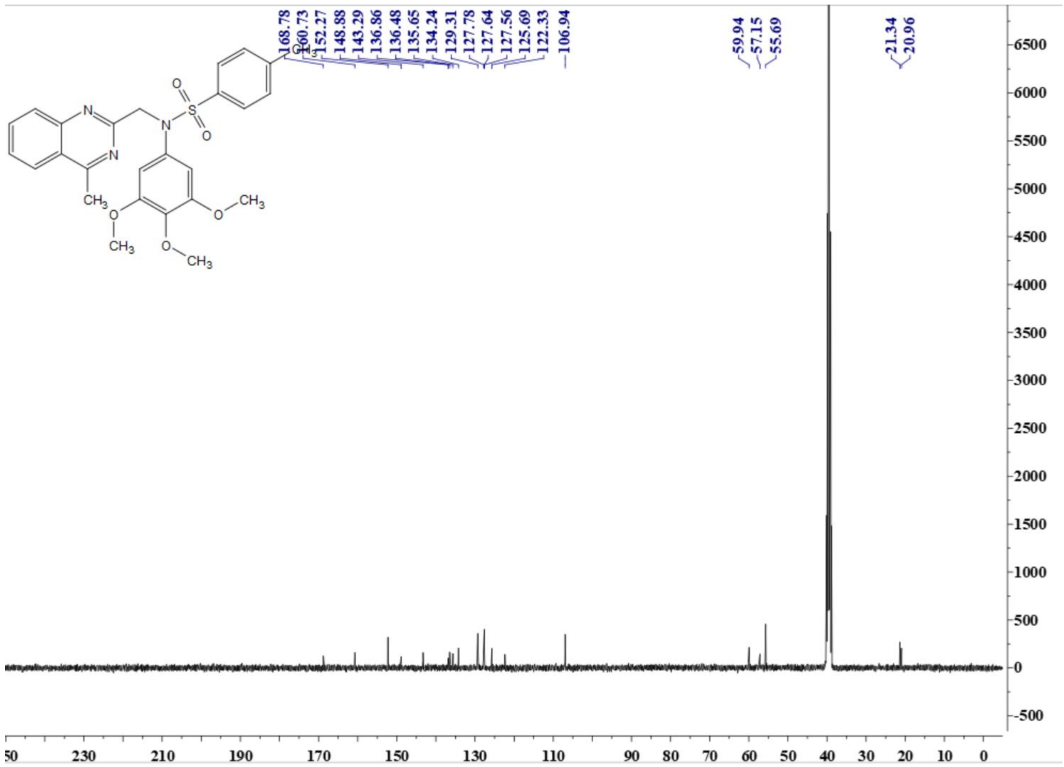

Figure S60. ^{13}C NMR spectrum of compound **10e** (100 MHz, DMSO-*d6*)

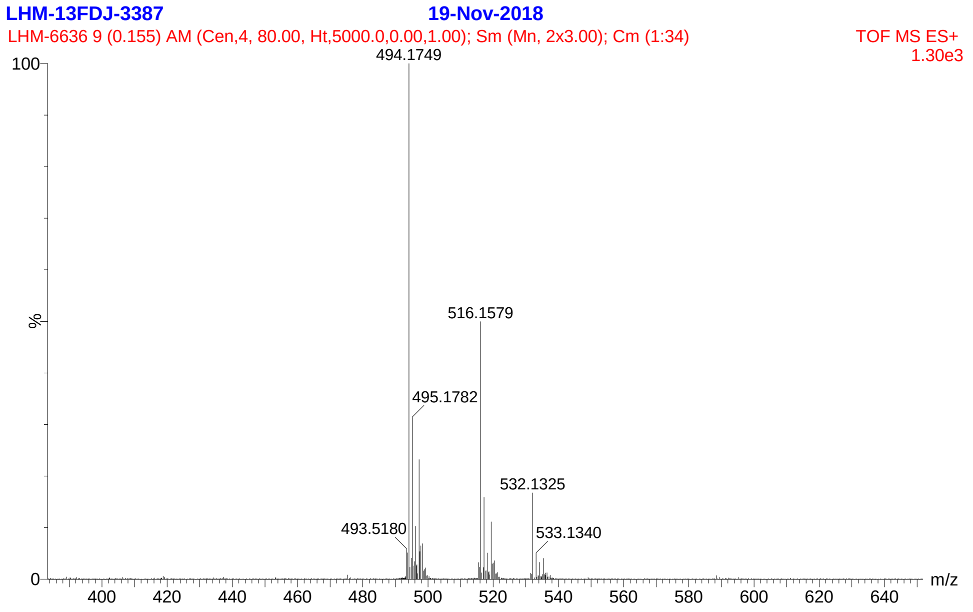

Figure S61. HRMS spectrum of compound **10e**

- ^{1}H, ^{13}C-NMR and HRMS of Compound **10f**

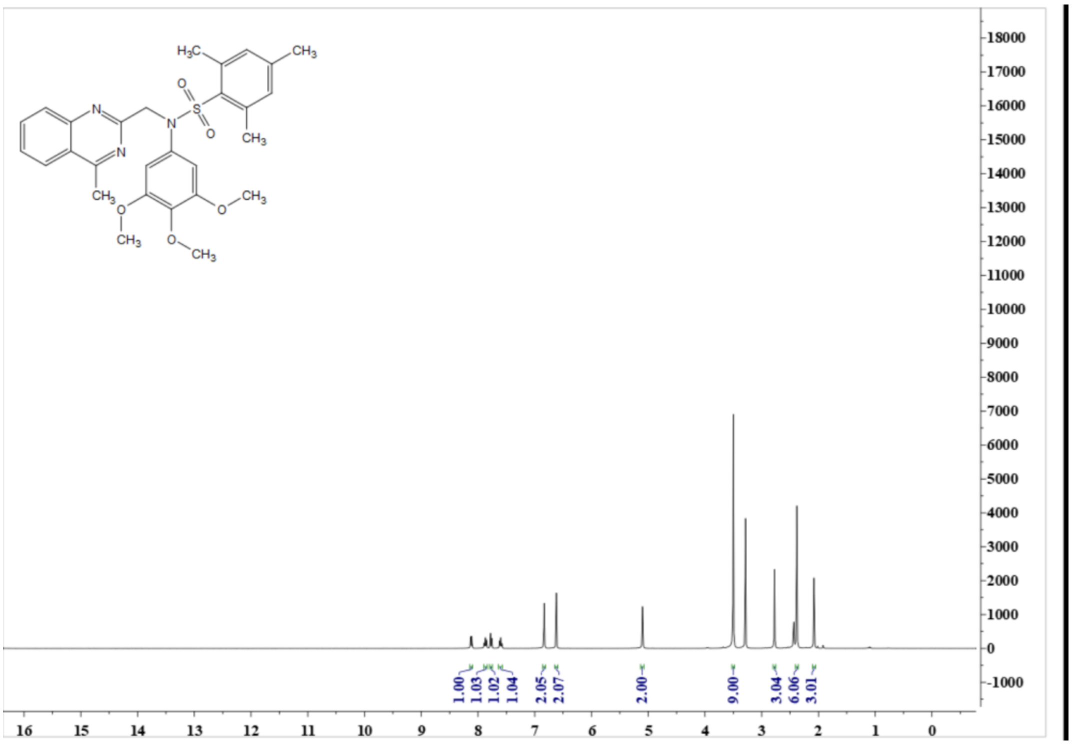

Figure S62. ^{1}H NMR spectrum of compound **10f** (400 MHz, DMSO-*d6*)

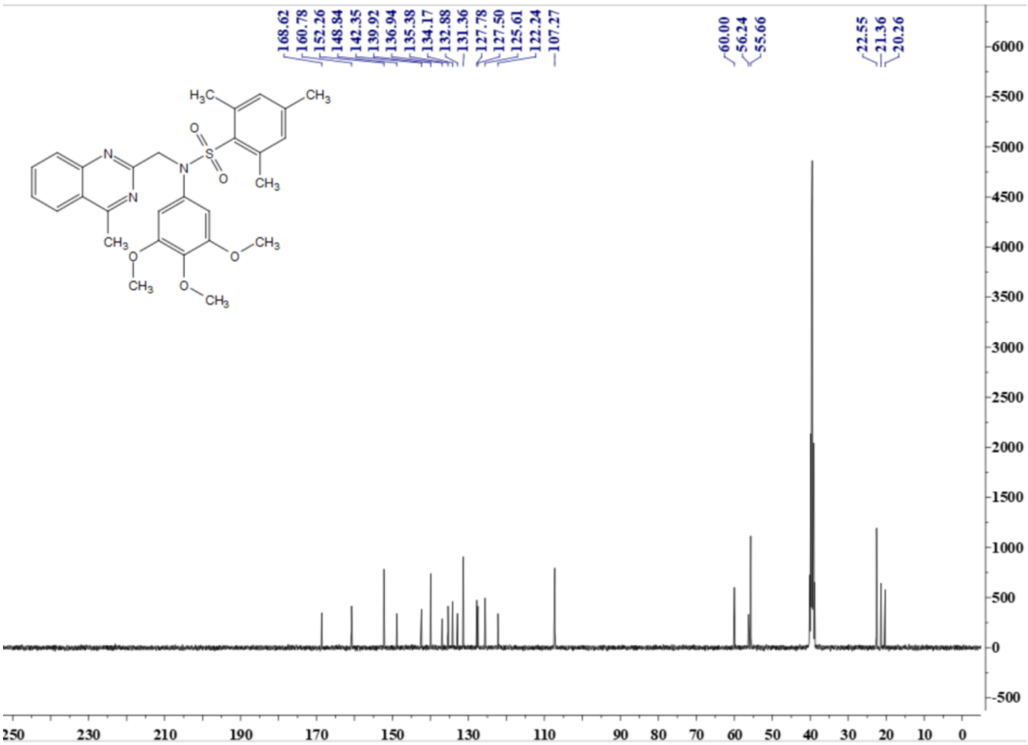

Figure S63. ^{13}C NMR spectrum of compound **10f** (100 MHz, DMSO-d_6)

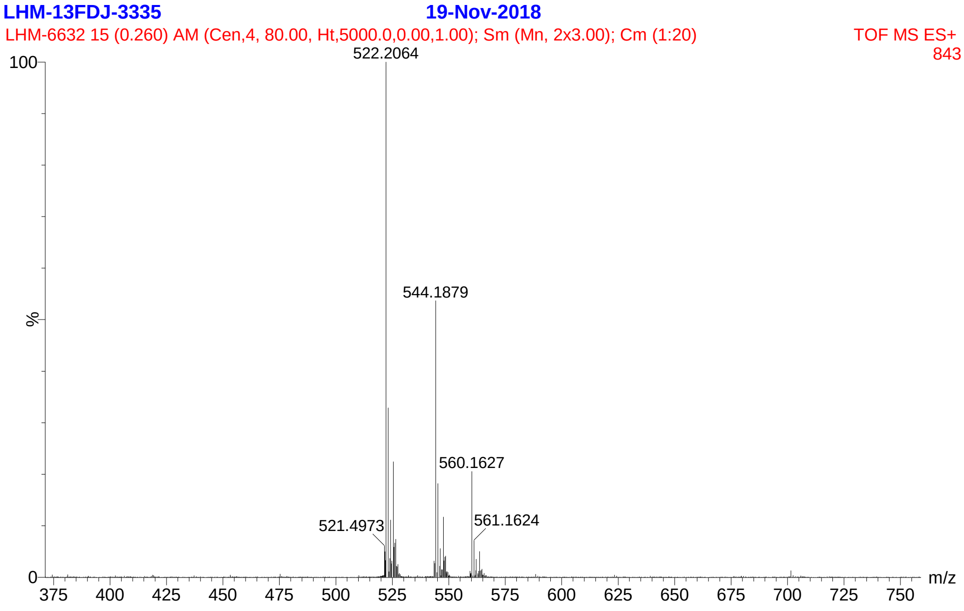

Figure S64. HRMS spectrum of compound **10f**

- ^{1}H, ^{13}C-NMR and HRMS of Compound **11g**

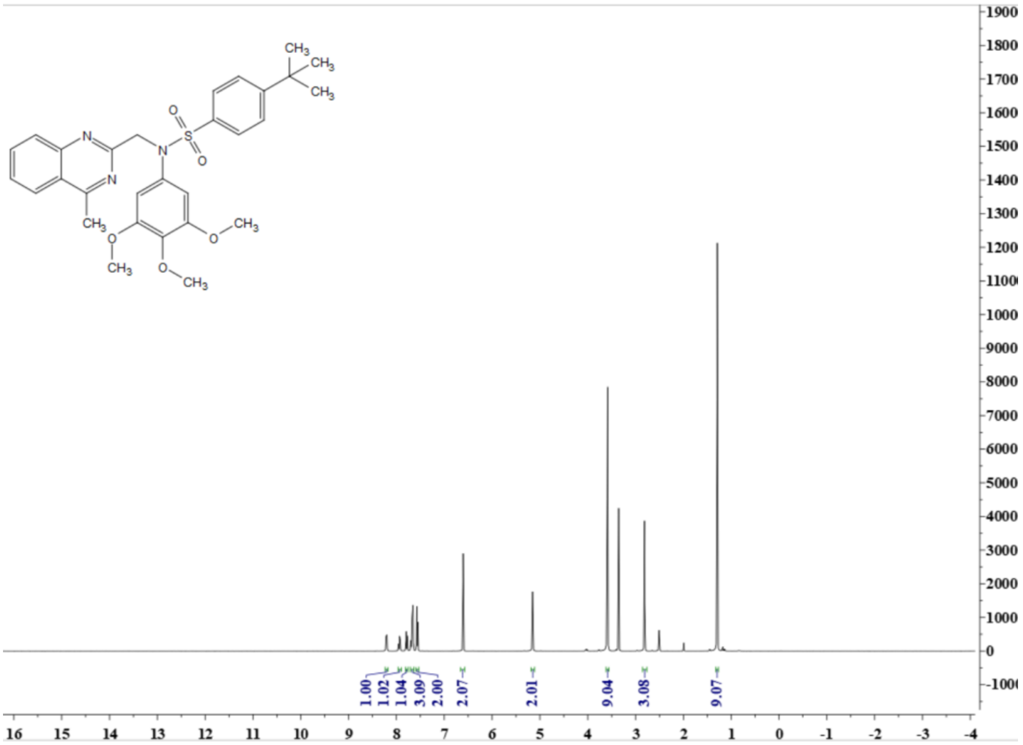

Figure S65. ^{1}H NMR spectrum of compound **11g** (400 MHz, DMSO-*d6*)

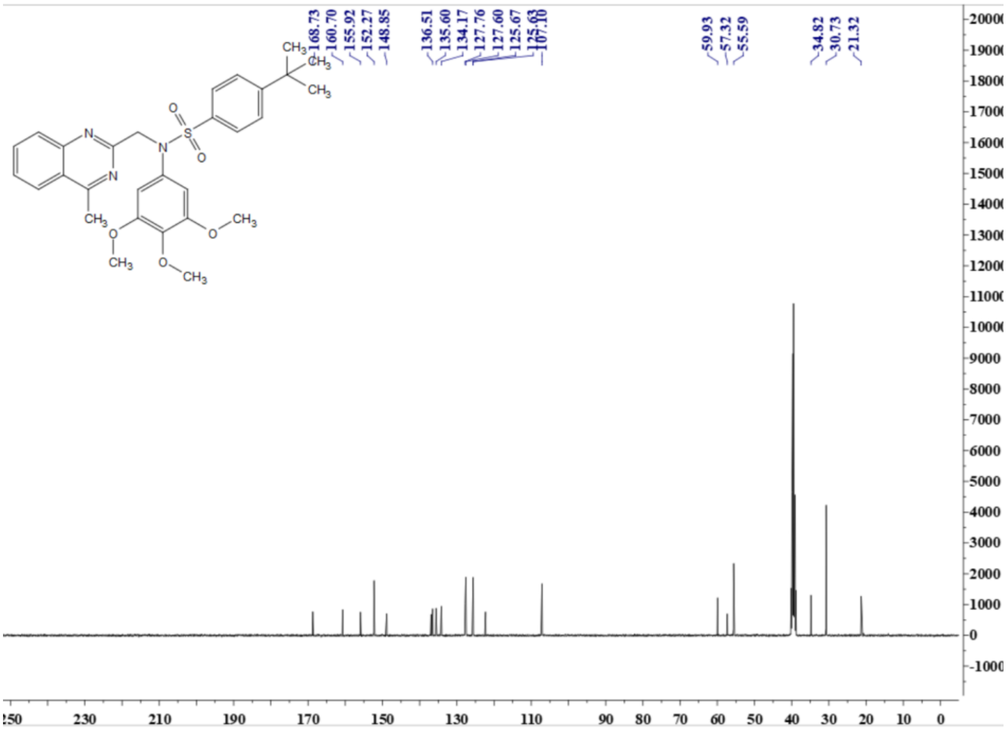

Figure S66. ^{13}C NMR spectrum of compound **10g** (100 MHz, DMSO-*d6*)

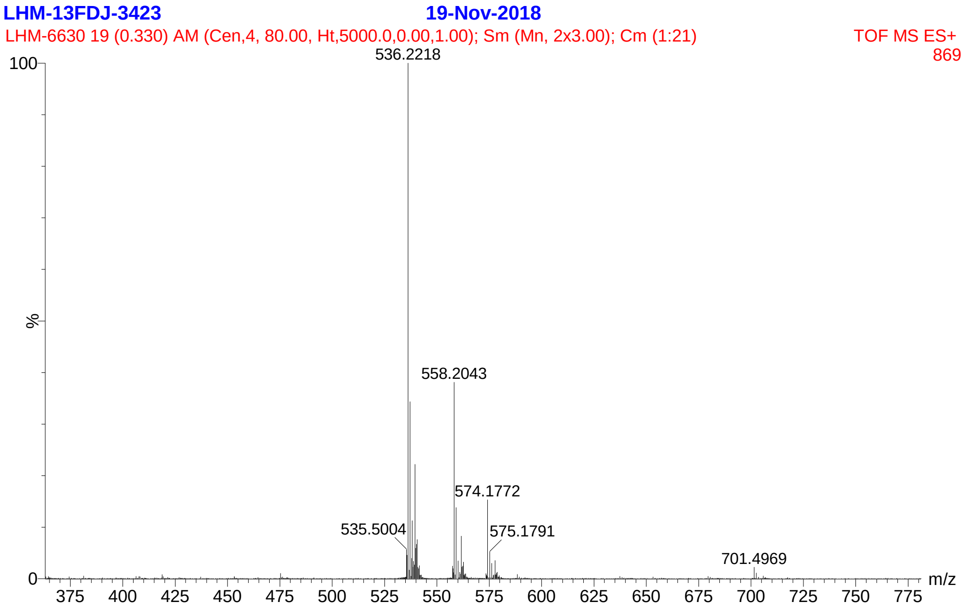

Figure S67. HRMS spectrum of compound **10g**

- ^{1}H, ^{13}C-NMR and HRMS of Compound **10h**

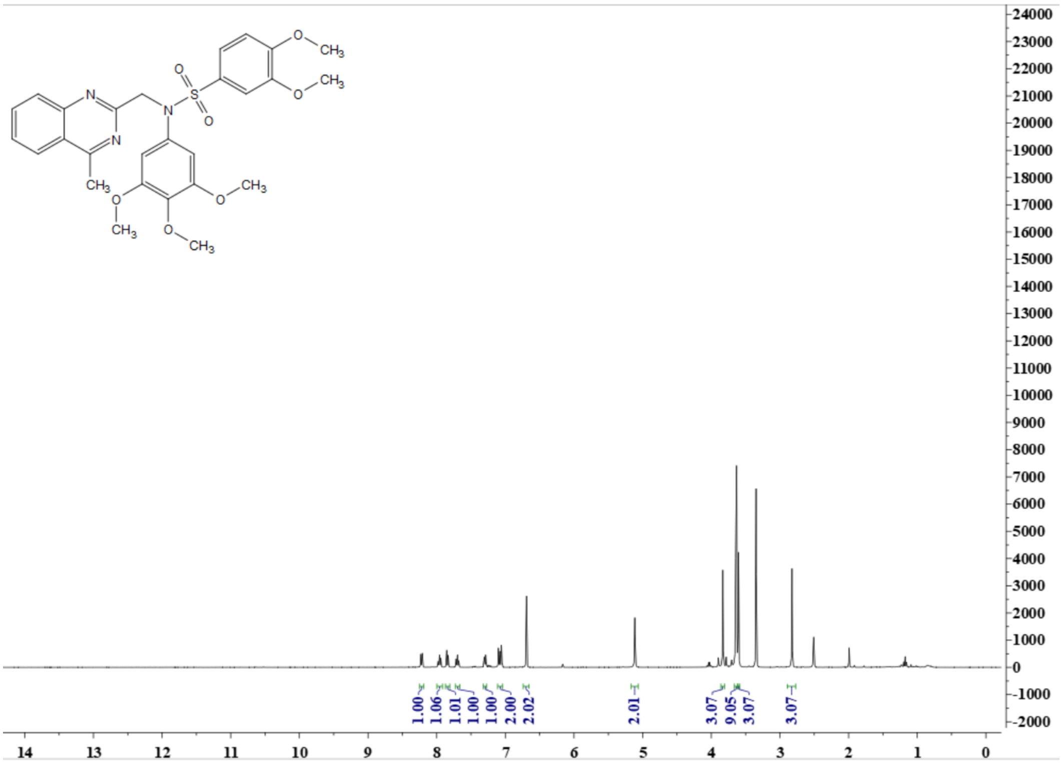

Figure S68. ^{1}H NMR spectrum of compound **10h** (400 MHz, DMSO-*d6*)

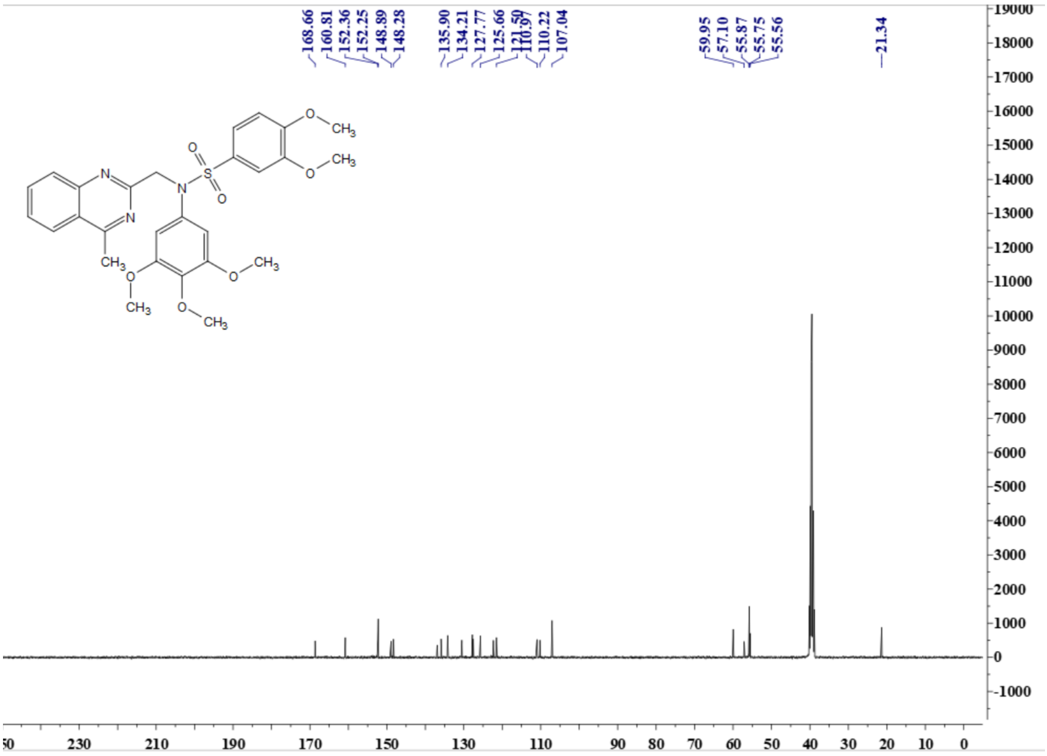

Figure S69. ^{13}C NMR spectrum of compound **10h** (100 MHz, DMSO-*d6*)

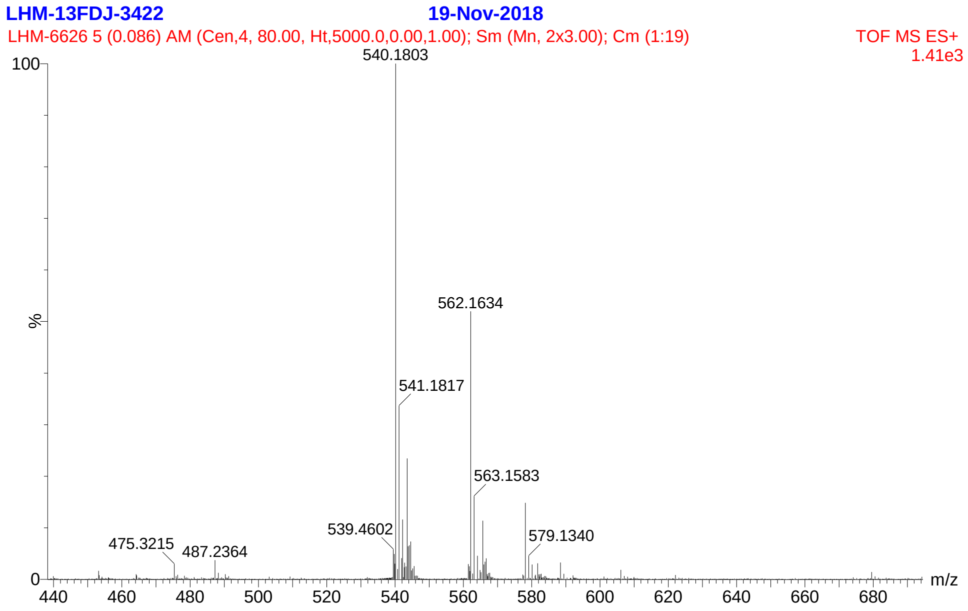

Figure S70. HRMS spectrum of compound **10h**

- ^{1}H, ^{13}C-NMR and HRMS of Compound **10i**

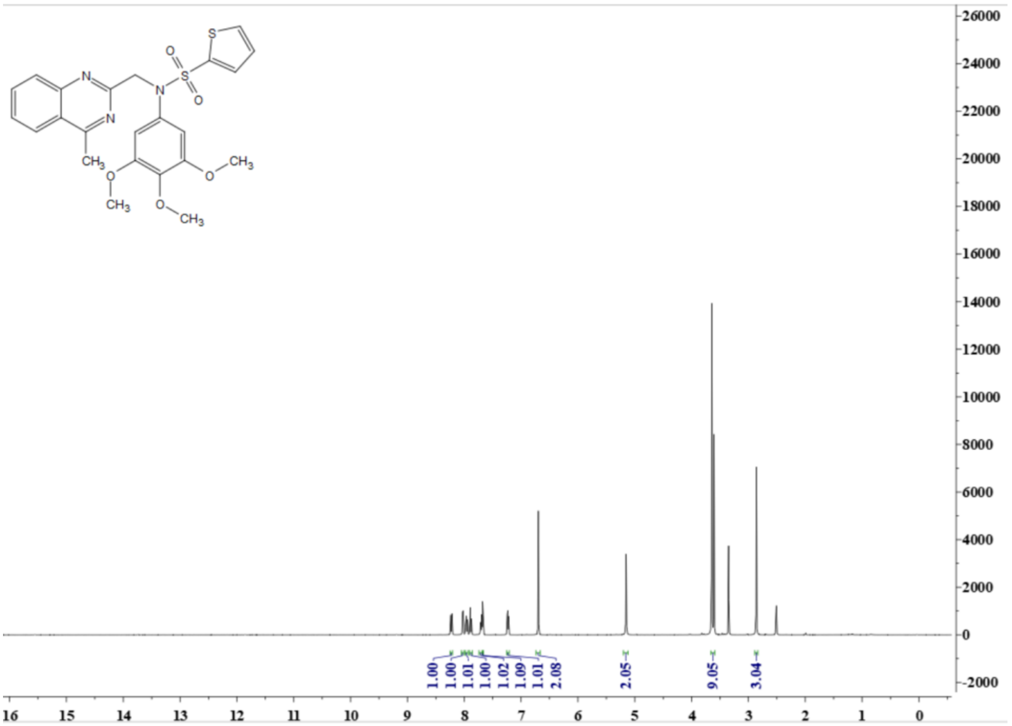

Figure S71. ^{1}H NMR spectrum of compound **10i** (400 MHz, DMSO-*d6*)

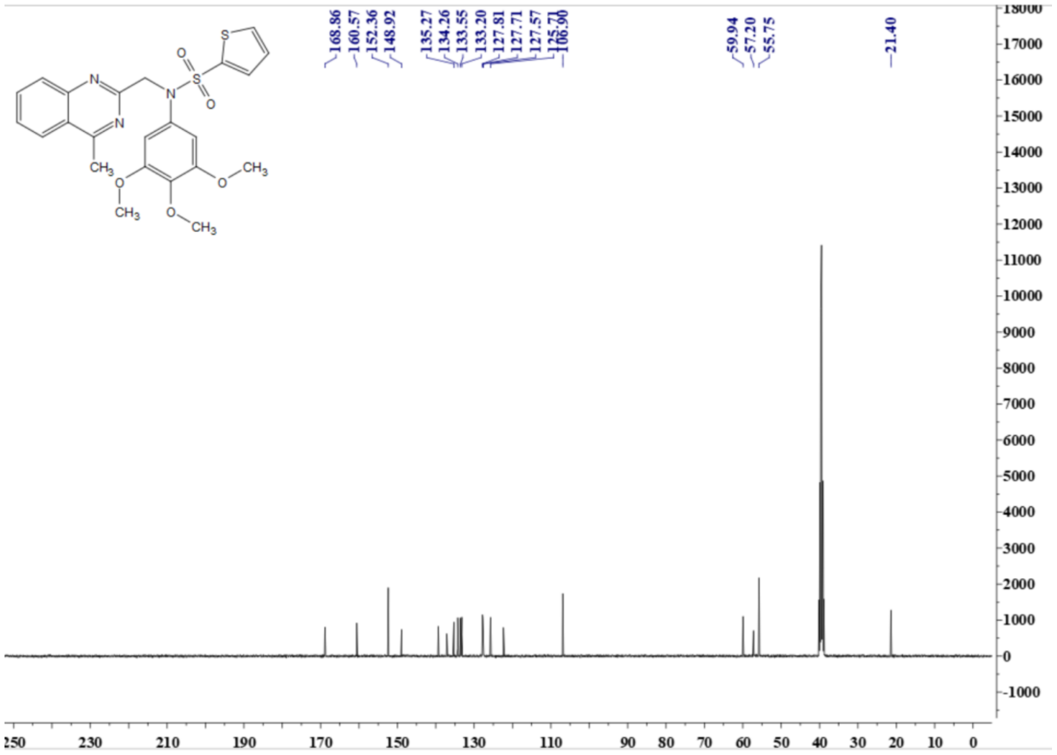

Figure S72. ^{13}C NMR spectrum of compound **10i** (100 MHz, DMSO-*d6*)

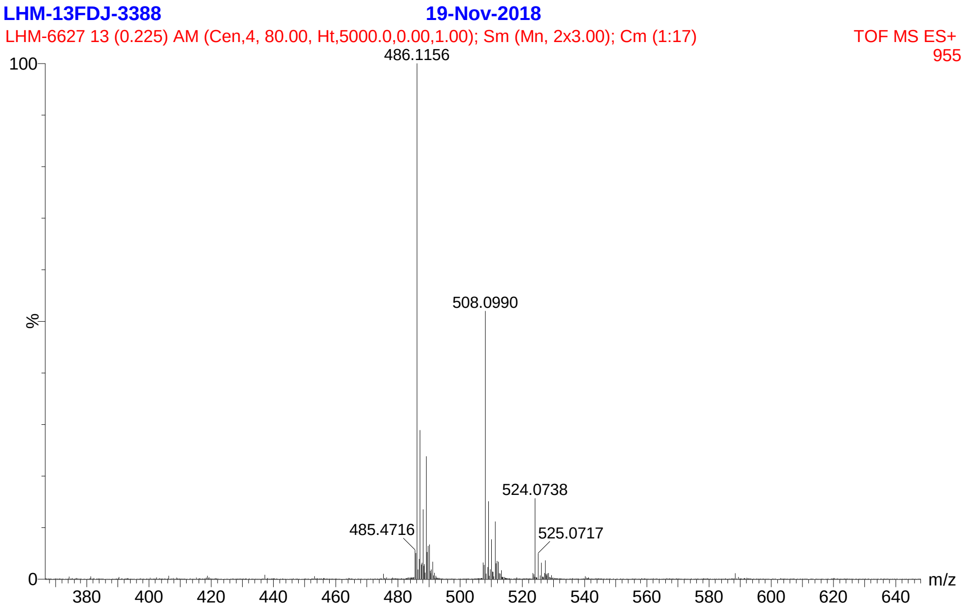

Figure S73. HRMS spectrum of compound **10i**

- ^{1}H, ^{13}C-NMR and HRMS of Compound **10j**

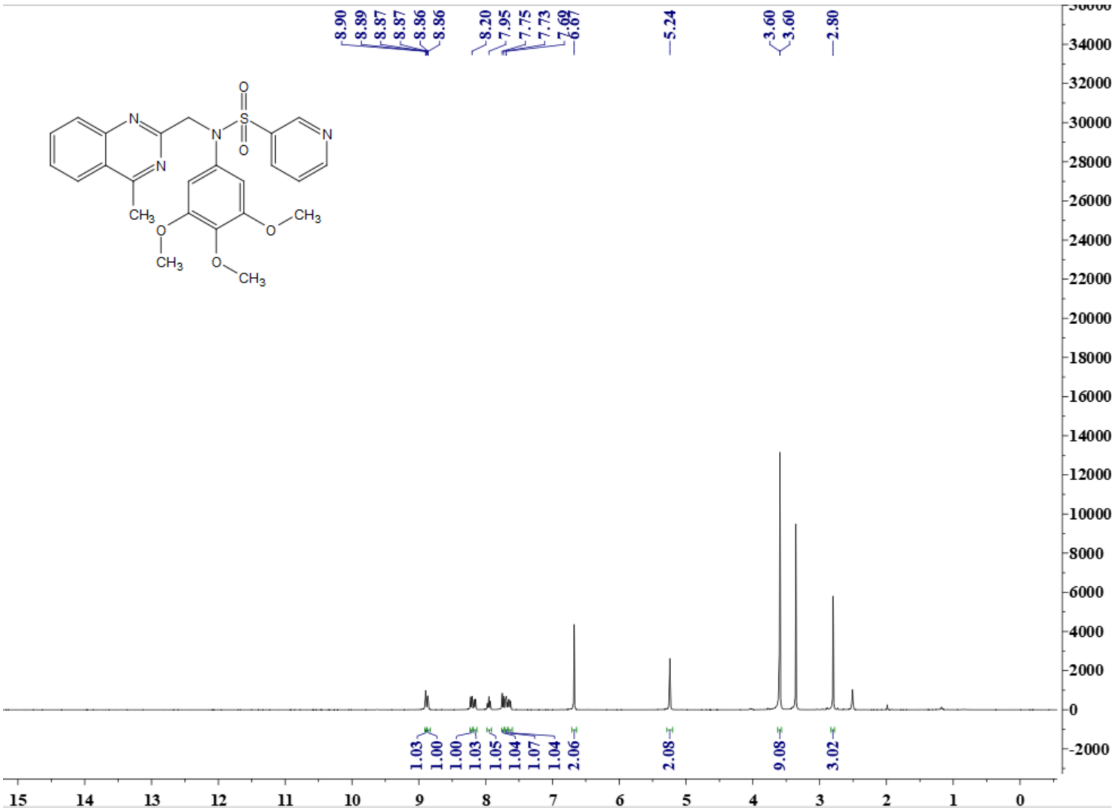

Figure S74. ^{1}H NMR spectrum of compound **10j** (400 MHz, DMSO-d_6)

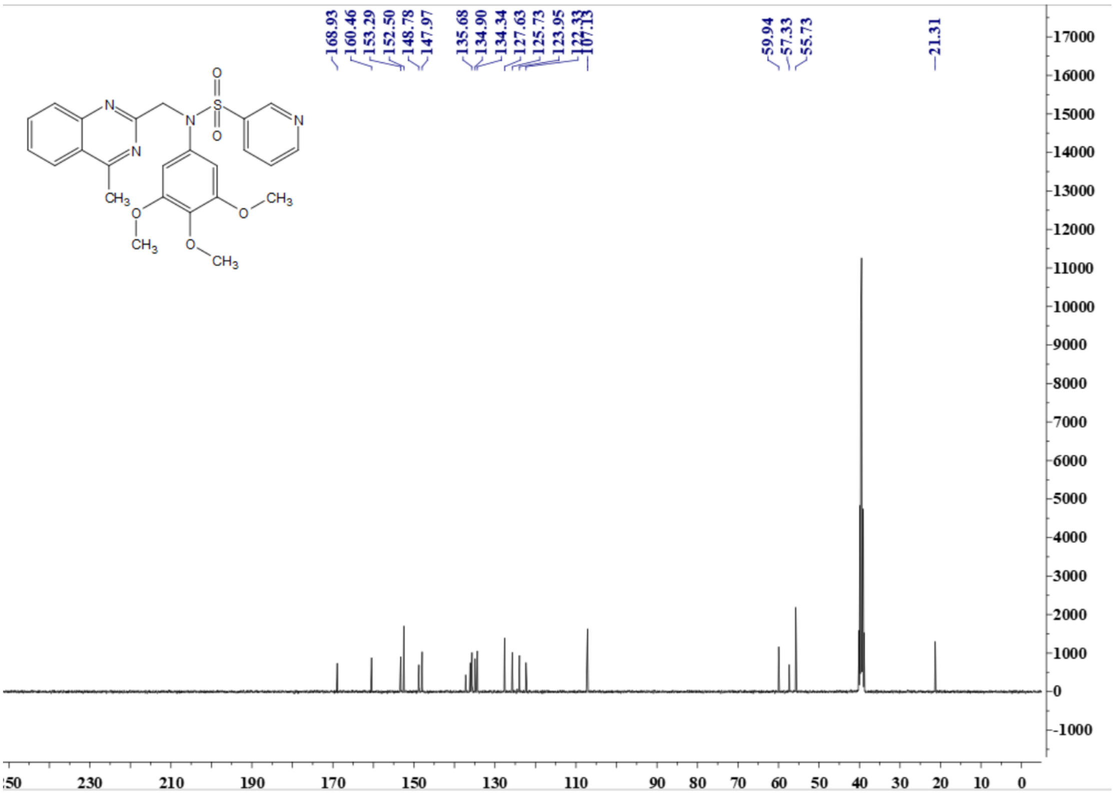

Figure S75. ^{13}C NMR spectrum of compound **10j** (100 MHz, DMSO-d_6)

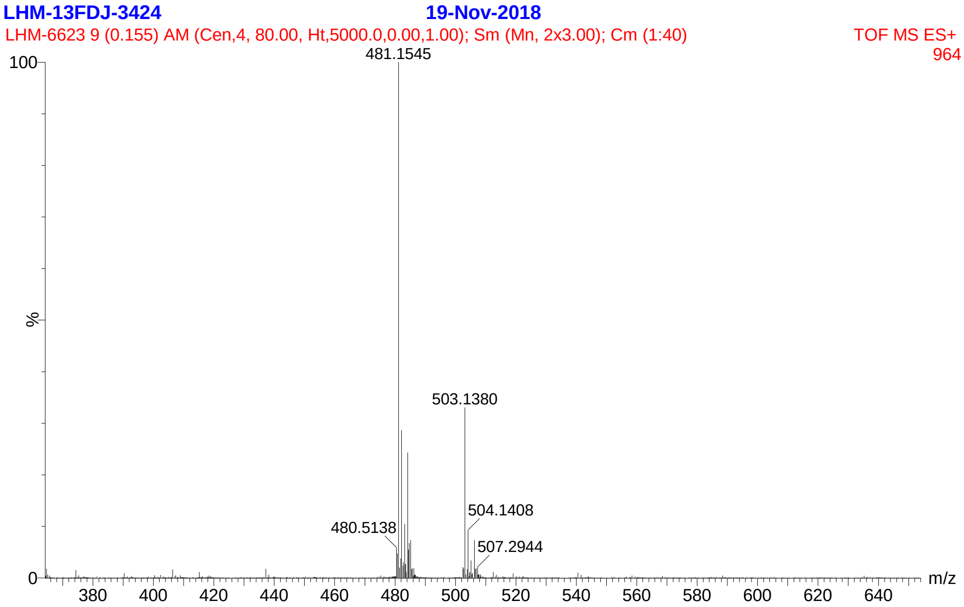

Figure S76. HRMS spectrum of compound **10j**

- ^{1}H, ^{13}C-NMR and HRMS of Compound **11a**

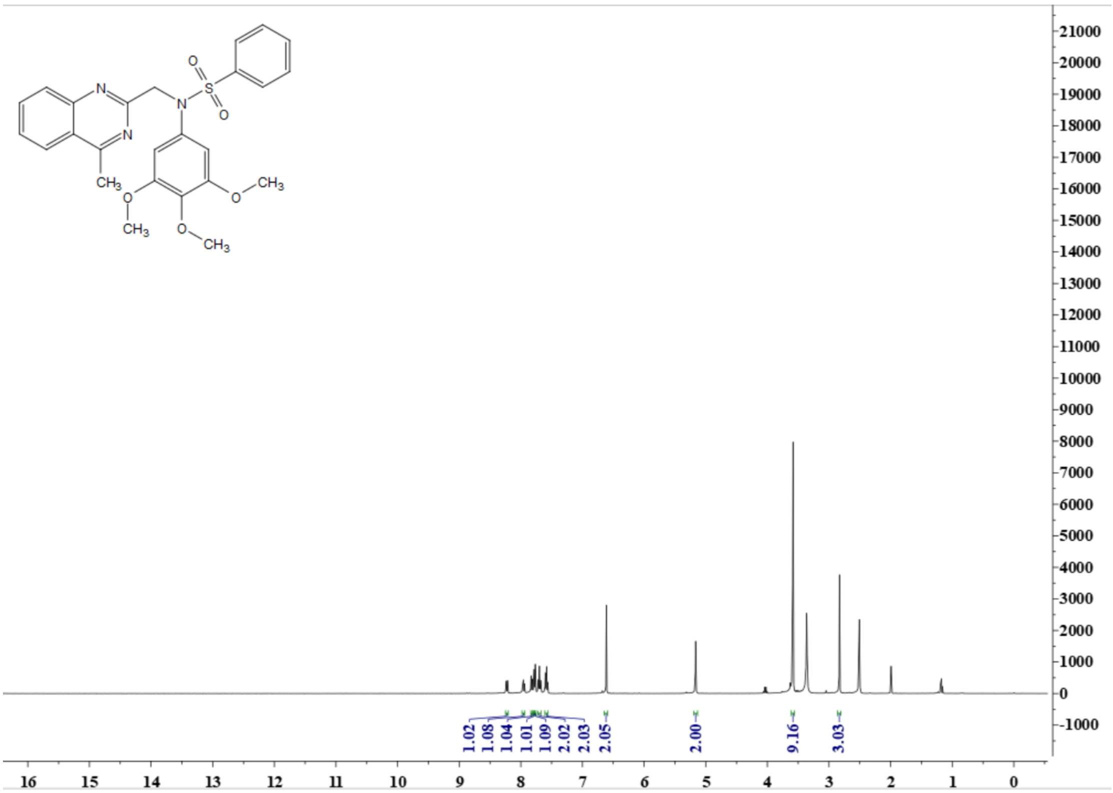

Figure S77. ^{1}H NMR spectrum of compound **11a** (400 MHz, DMSO-*d6*)

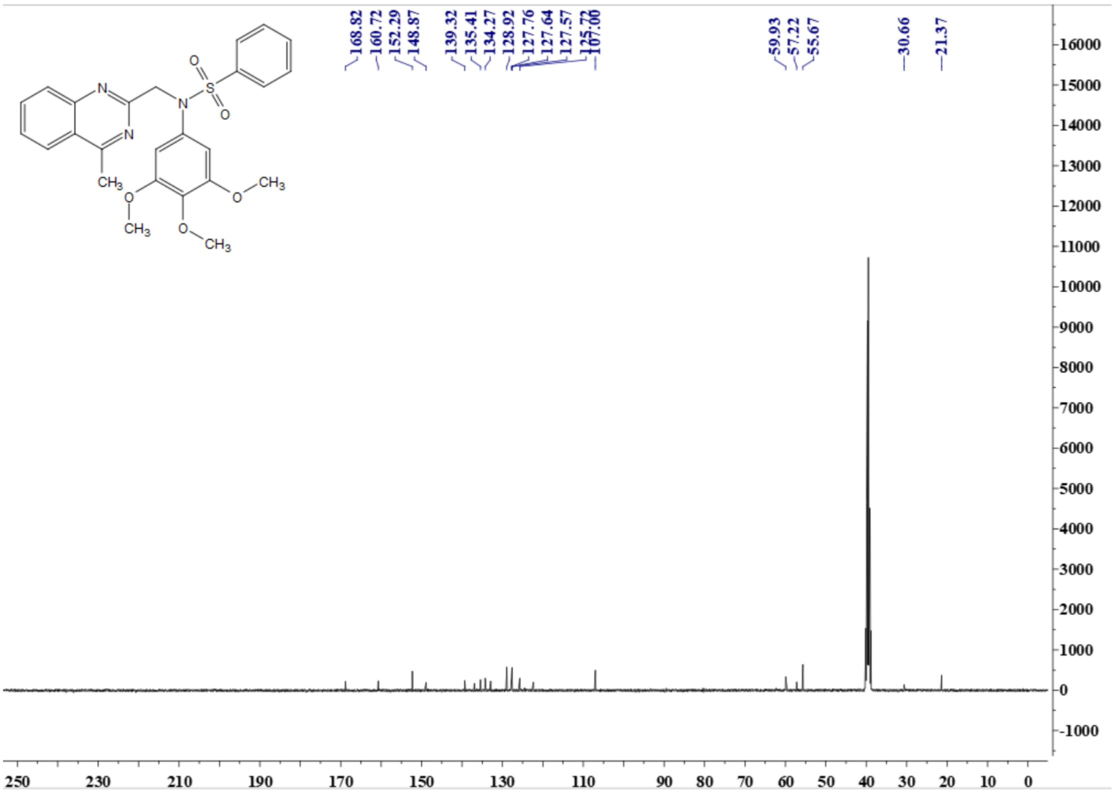

Figure S78. ^{13}C NMR spectrum of compound **11a** (100 MHz, DMSO-*d6*)

- ^{1}H, ^{13}C-NMR and HRMS of Compound **11b**

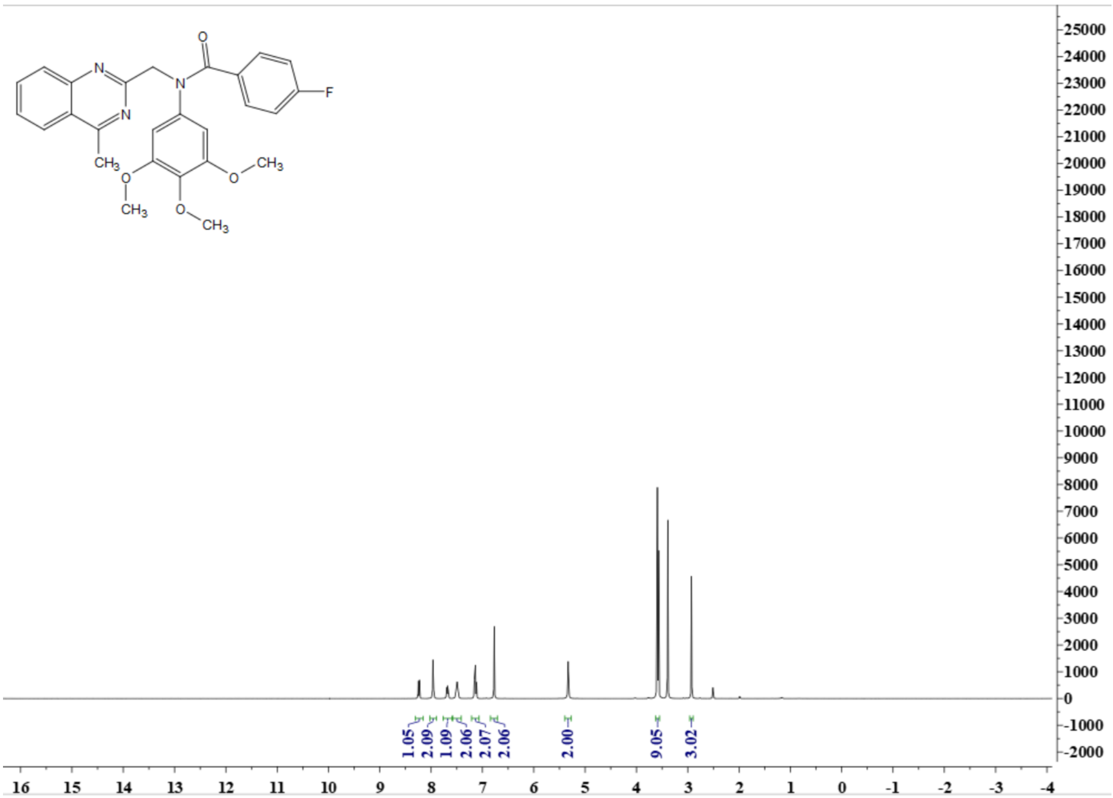

Figure S79. ^{1}H NMR spectrum of compound **11b** (400 MHz, DMSO-d_6)

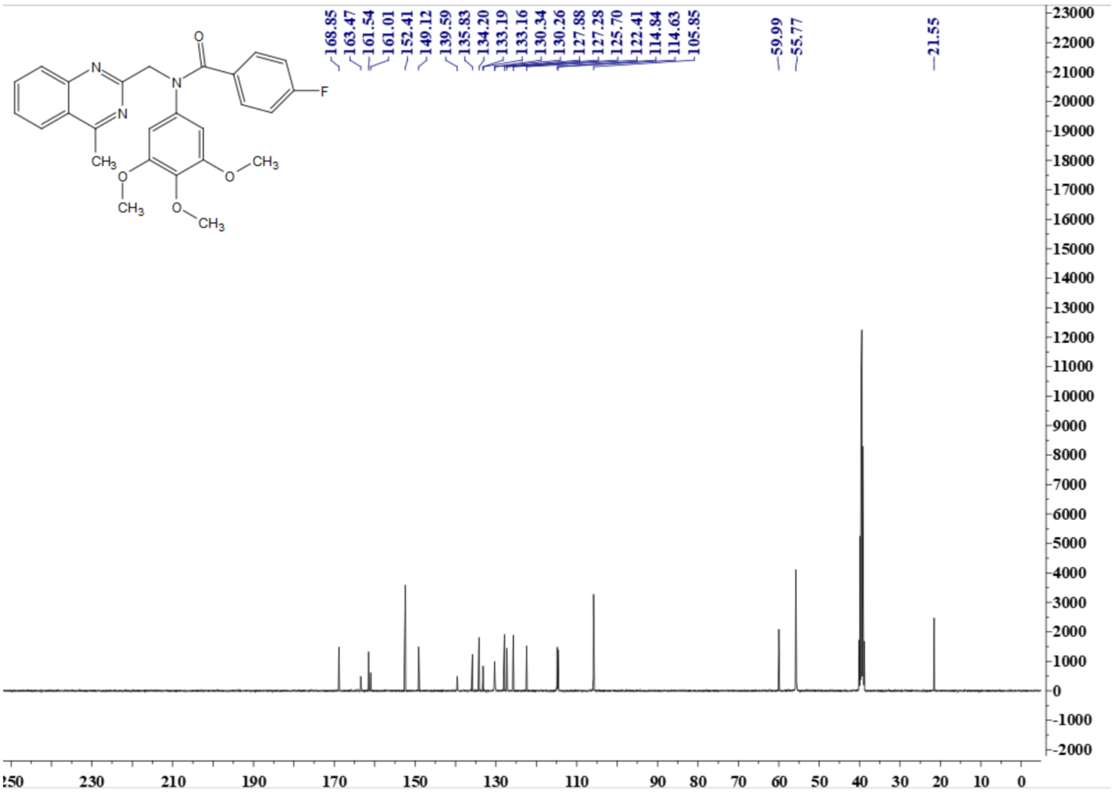

Figure S80. ^{13}C NMR spectrum of compound **11b** (100 MHz, DMSO-d_6)

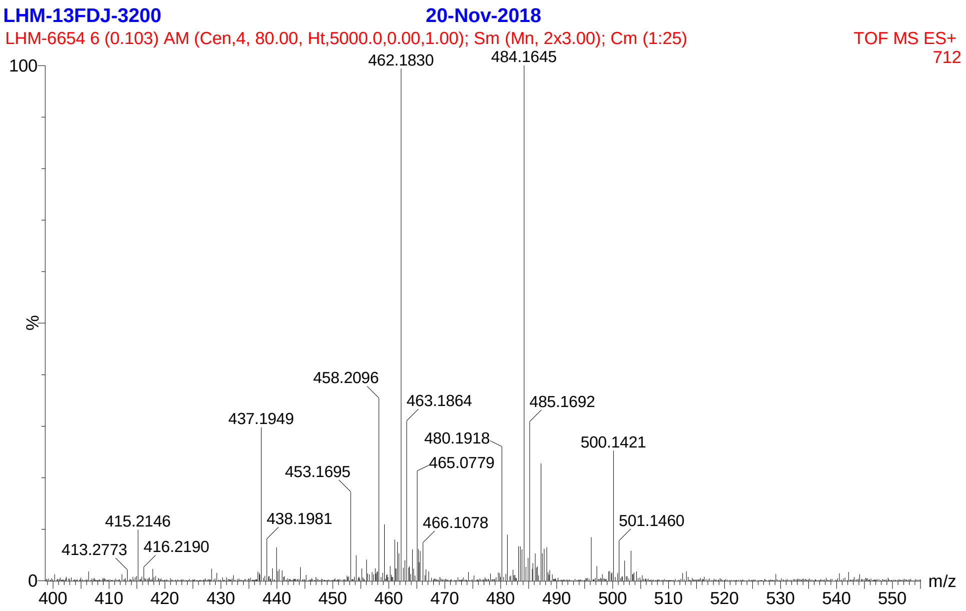

Figure S81. HRMS spectrum of compound **11b**

- ^{1}H, ^{13}C-NMR and HRMS of Compound **11c**

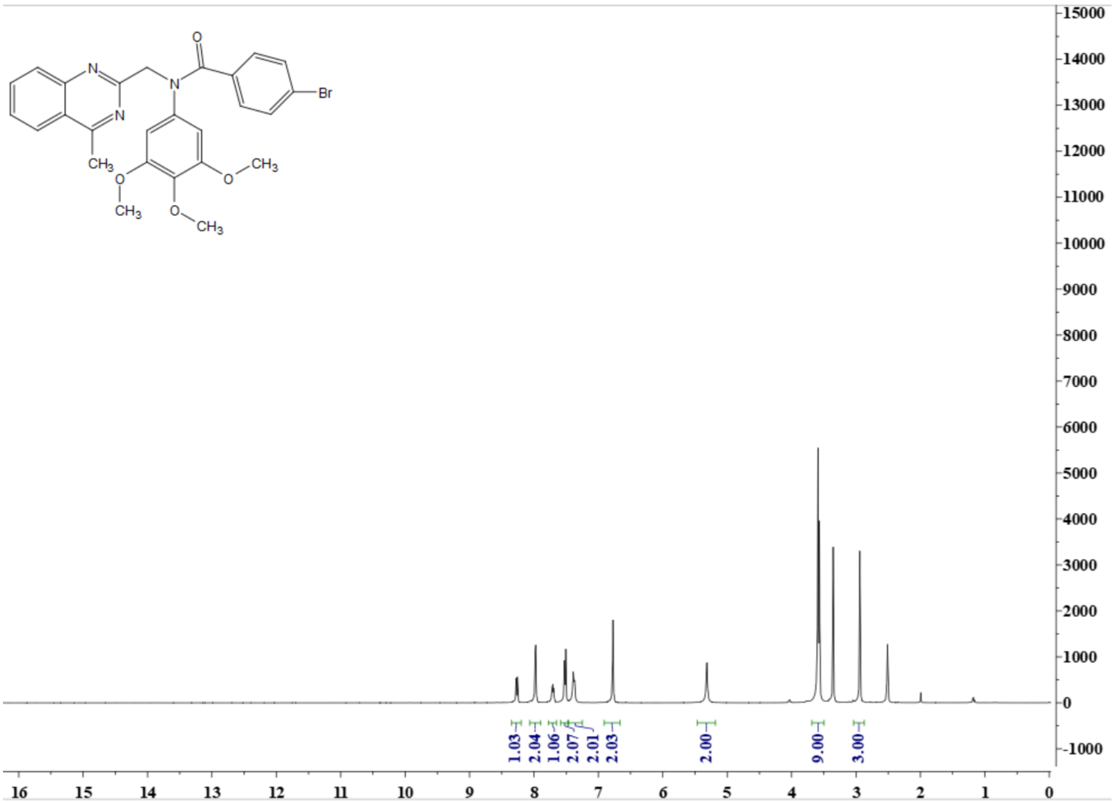

Figure S82. ^{1}H NMR spectrum of compound **11c** (400 MHz, DMSO-*d6*)

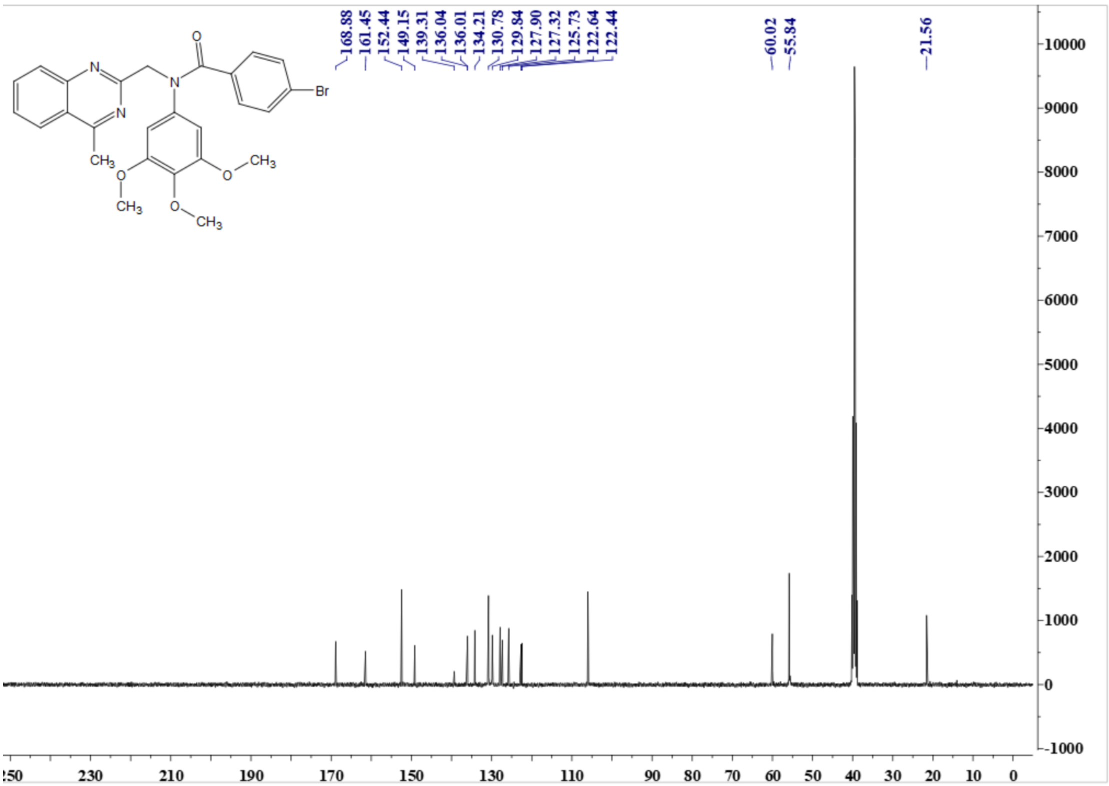

Figure S83. ^{13}C NMR spectrum of compound **11c** (100 MHz, DMSO-*d6*)

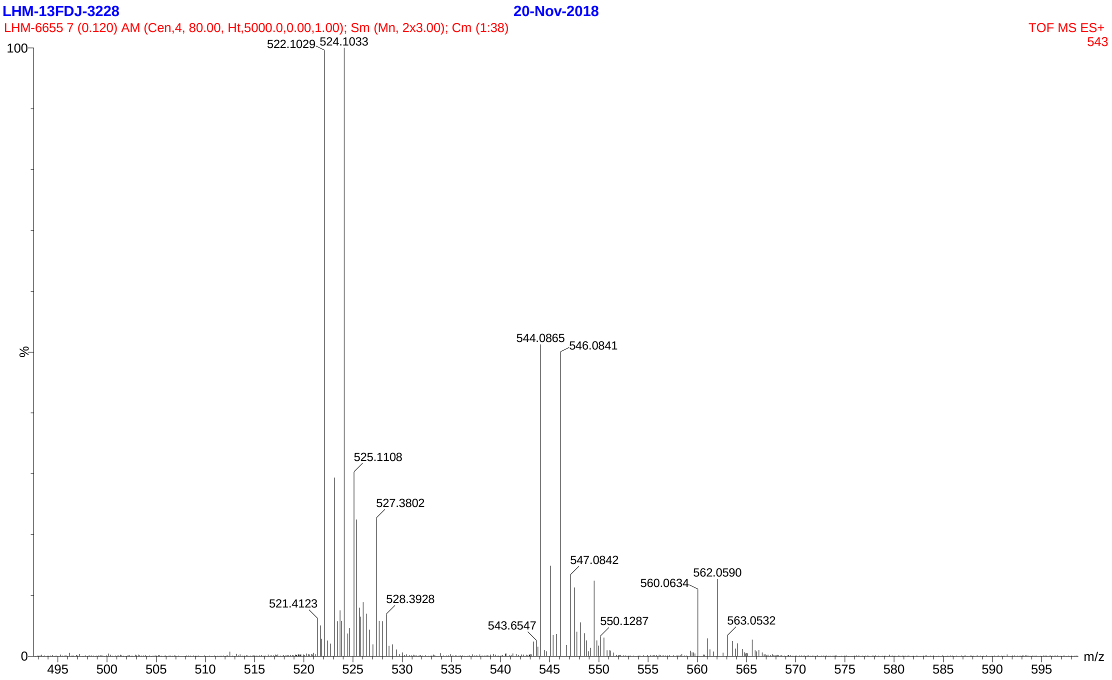

Figure S83. HRMS spectrum of compound **11c**

- ^{1}H, ^{13}C-NMR and HRMS of Compound **11d**

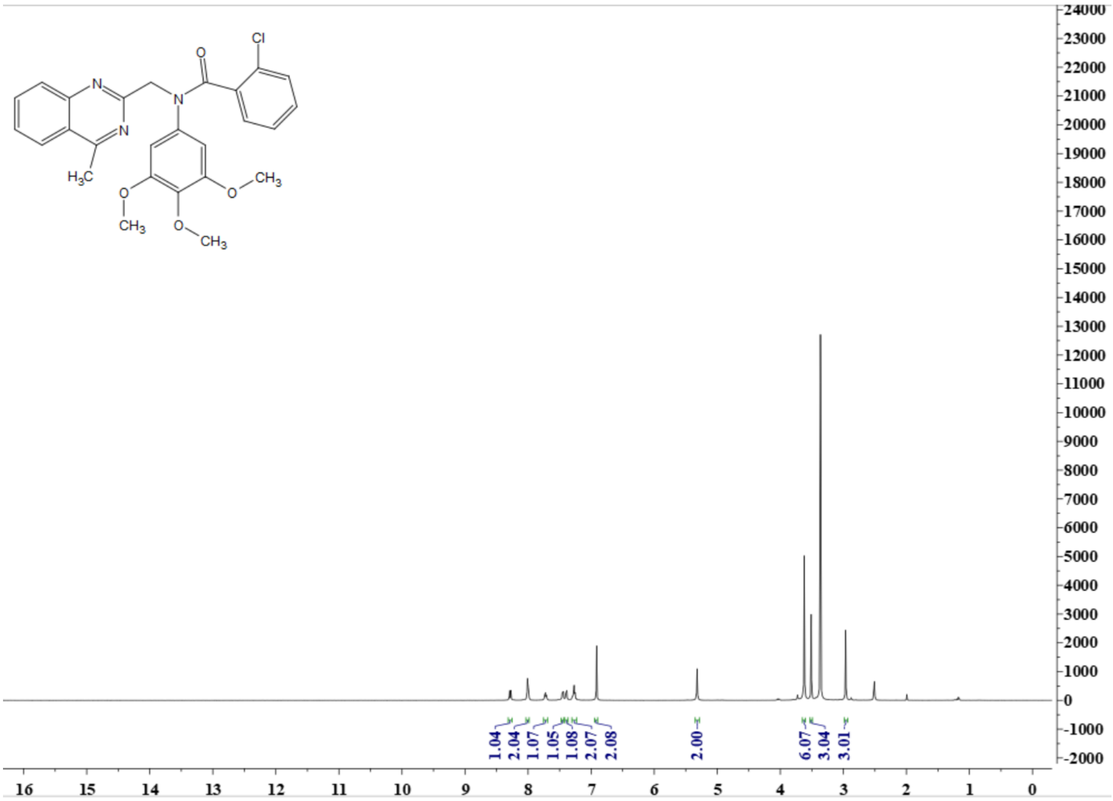

Figure S84. ^{1}H NMR spectrum of compound **11d** (400 MHz, DMSO-*d6*)

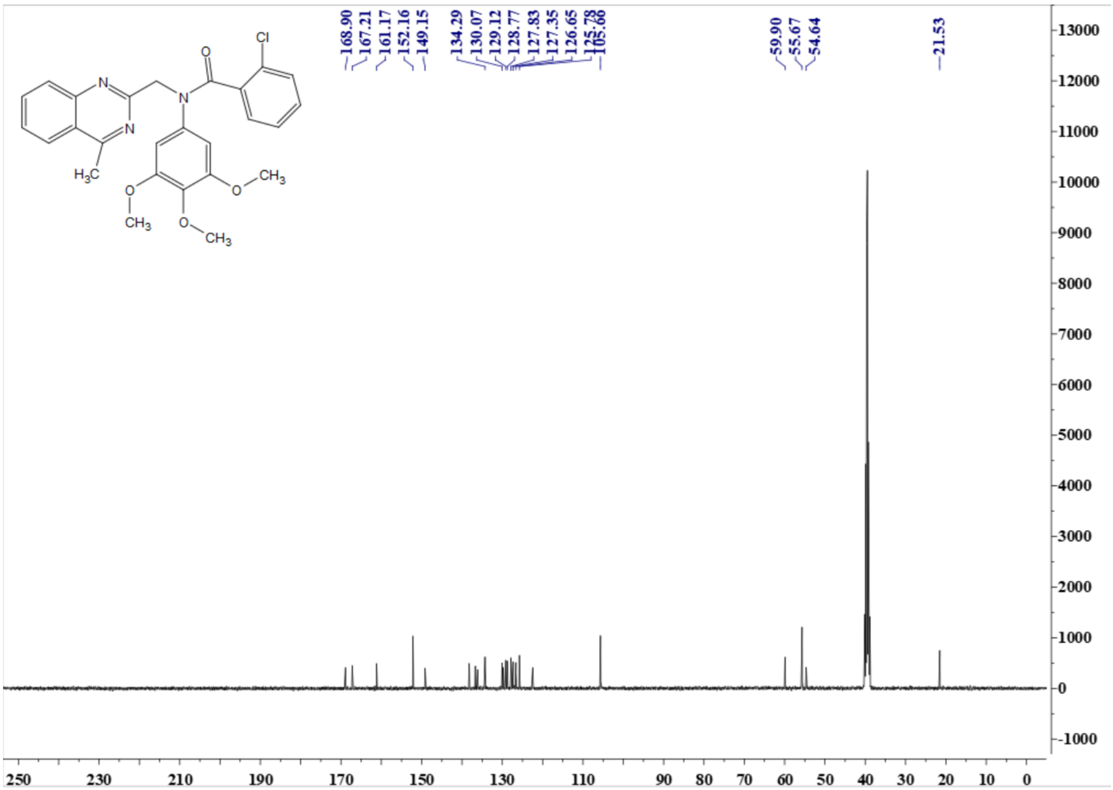

Figure S85. ^{13}C NMR spectrum of compound **11d** (100 MHz, DMSO-*d6*)

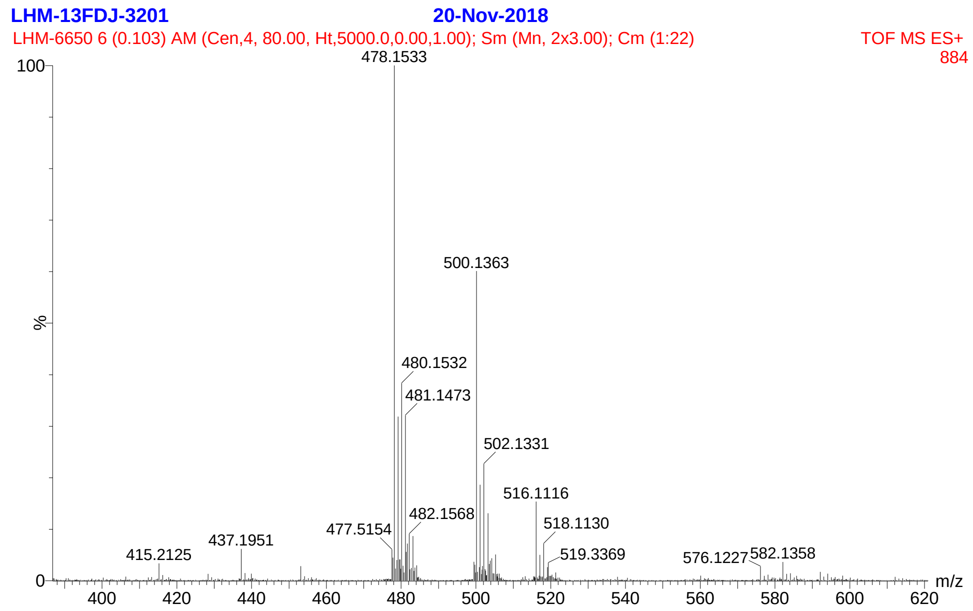

Figure S86. HRMS spectrum of compound **11d**

- ^{1}H, ^{13}C-NMR and HRMS of Compound **11e**

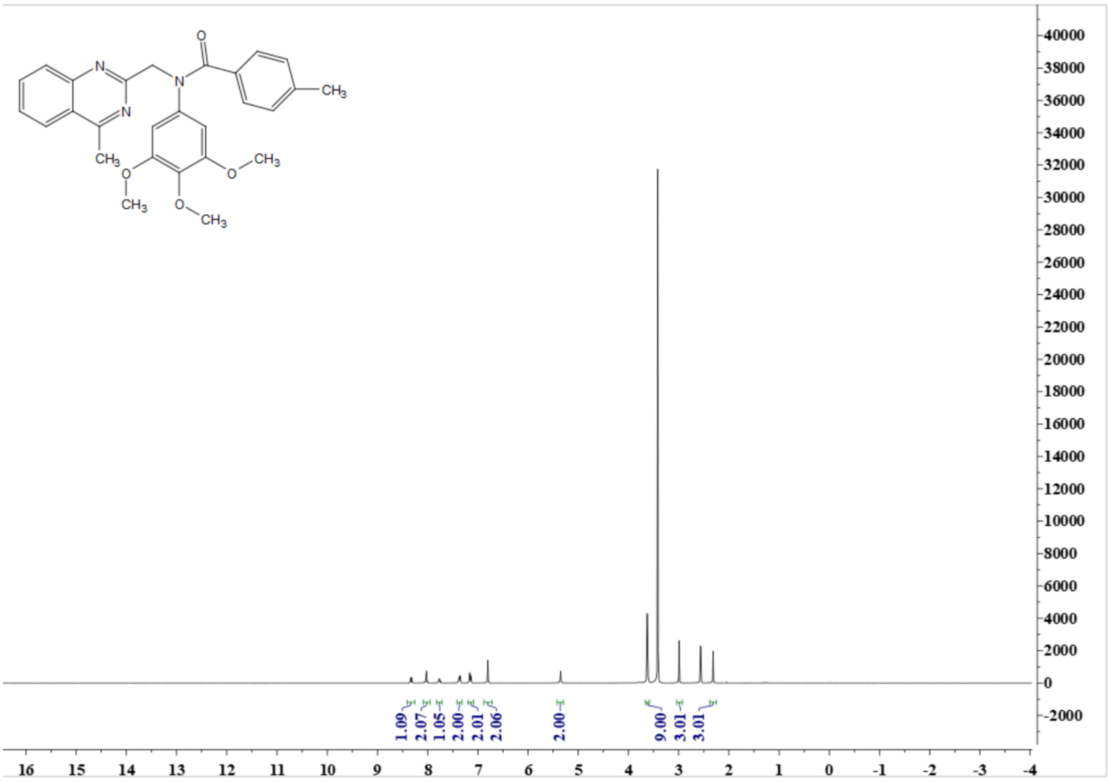

Figure S87. ^{1}H NMR spectrum of compound **11e** (400 MHz, DMSO-d_6)

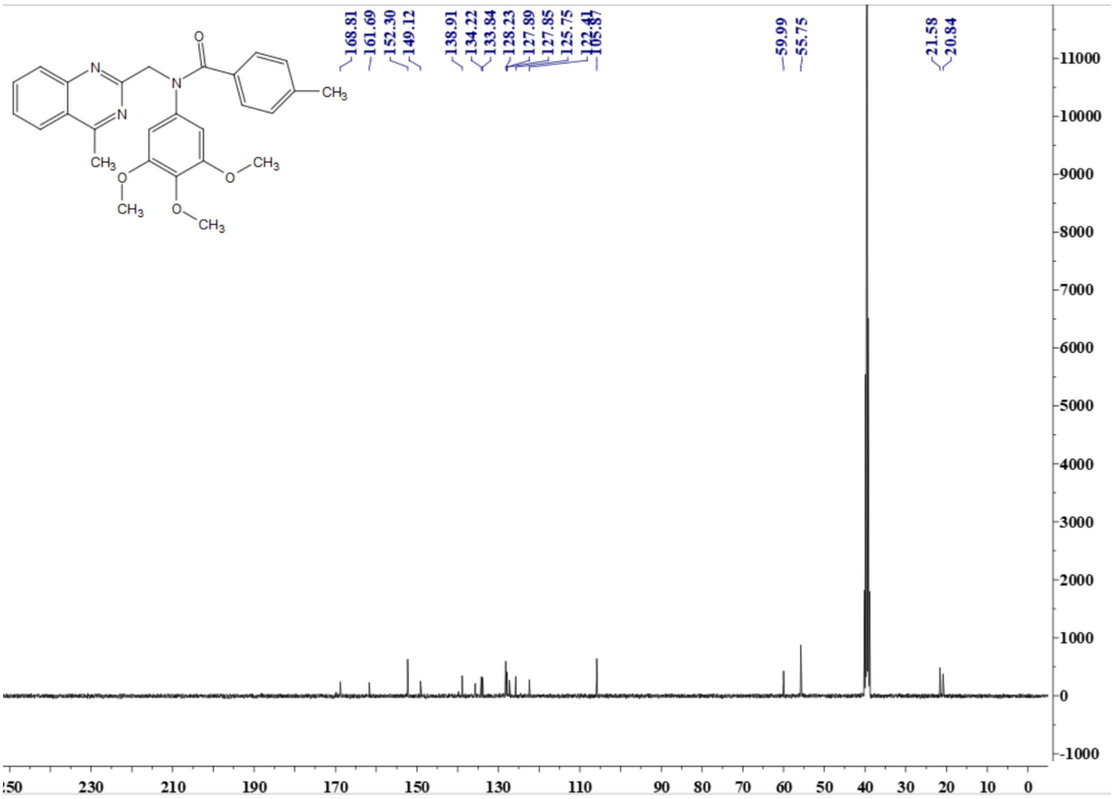

Figure S88. ^{13}C NMR spectrum of compound **11e** (100 MHz, DMSO-d_6)

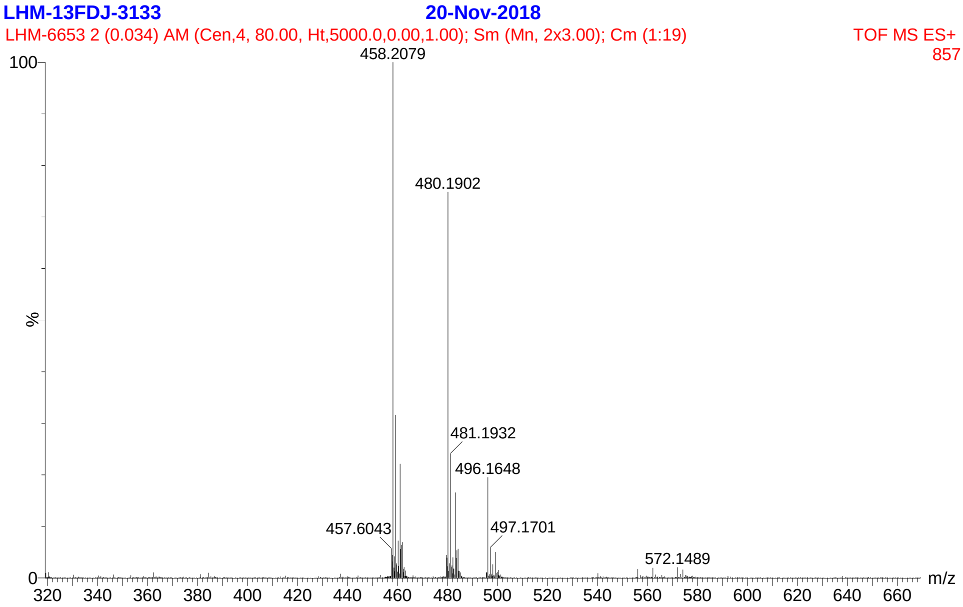

Figure S89. HRMS spectrum of compound **11e**

- ^{1}H, ^{13}C-NMR and HRMS of Compound **11f**

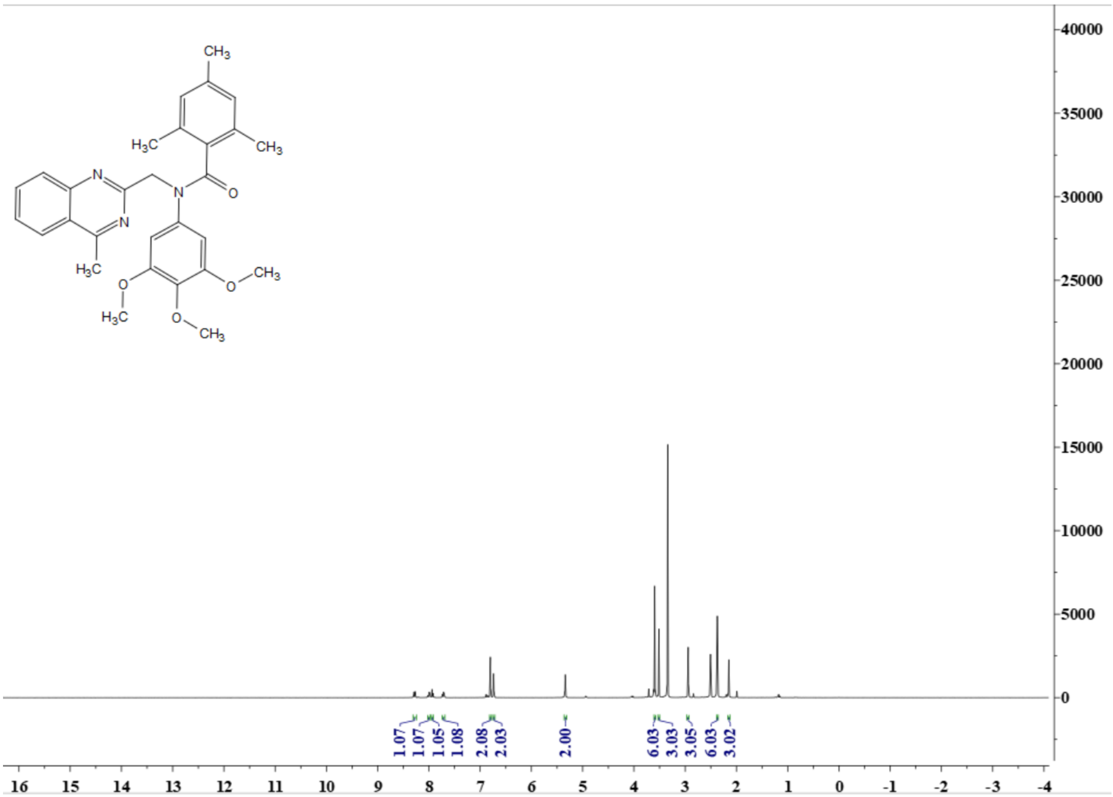

Figure S90. ^{1}H NMR spectrum of compound **11f** (400 MHz, DMSO-*d6*)

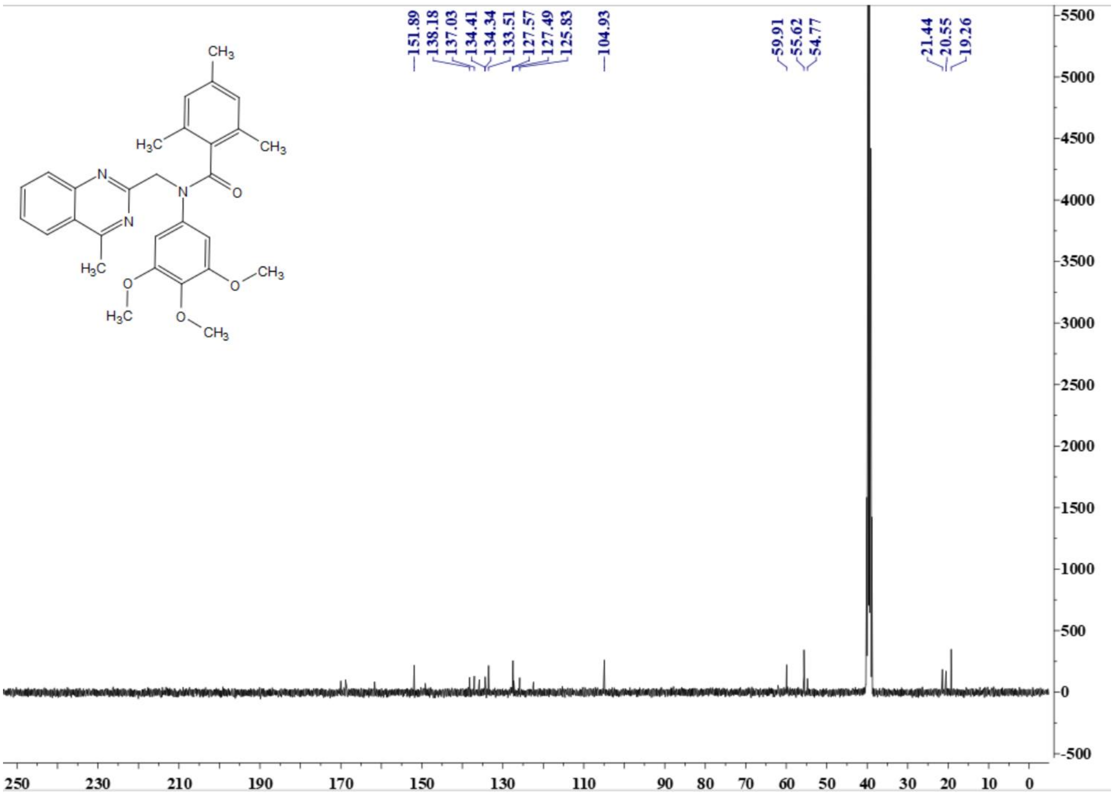

Figure S91. ^{13}C NMR spectrum of compound **11f** (100 MHz, DMSO-*d6*)

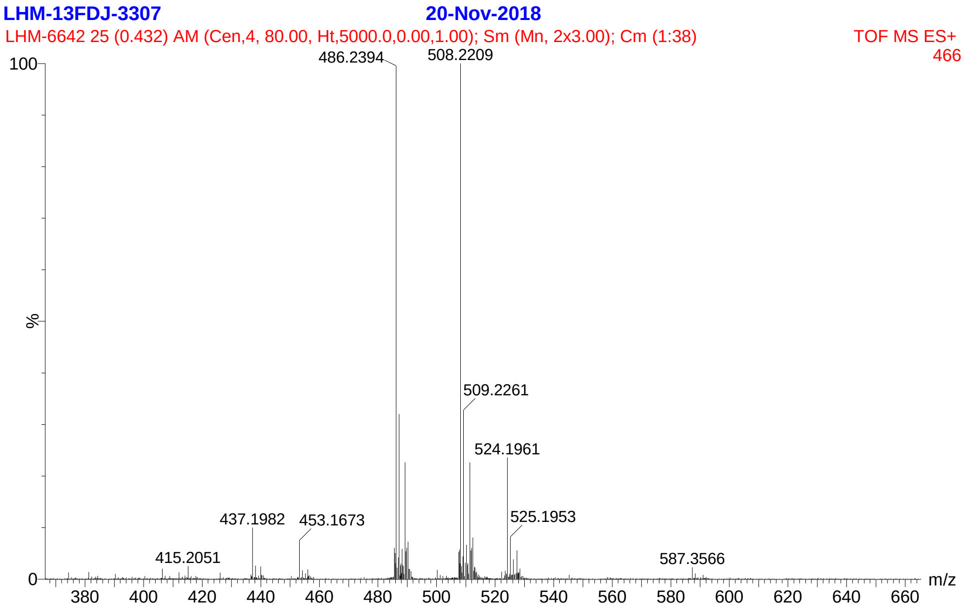

Figure S92. HRMS spectrum of compound **11f**

- ^{1}H, ^{13}C-NMR and HRMS of Compound **11g**

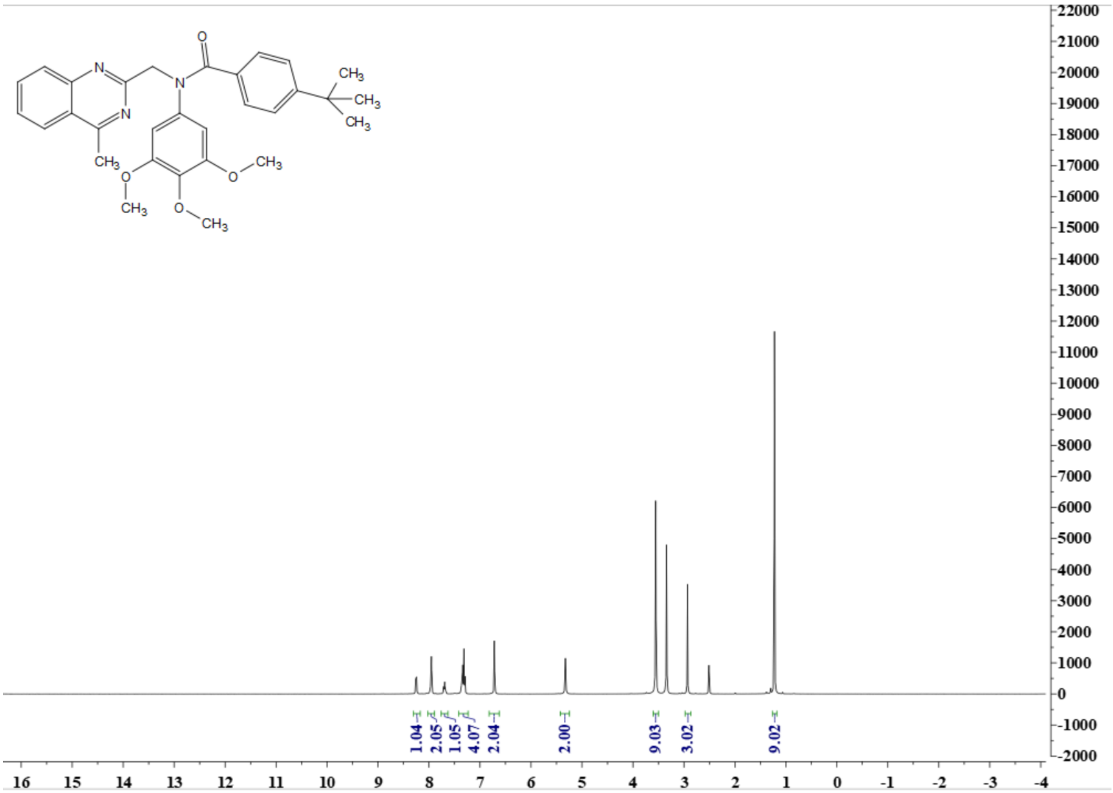

Figure S93. ^{1}H NMR spectrum of compound **11g** (400 MHz, DMSO-*d6*)

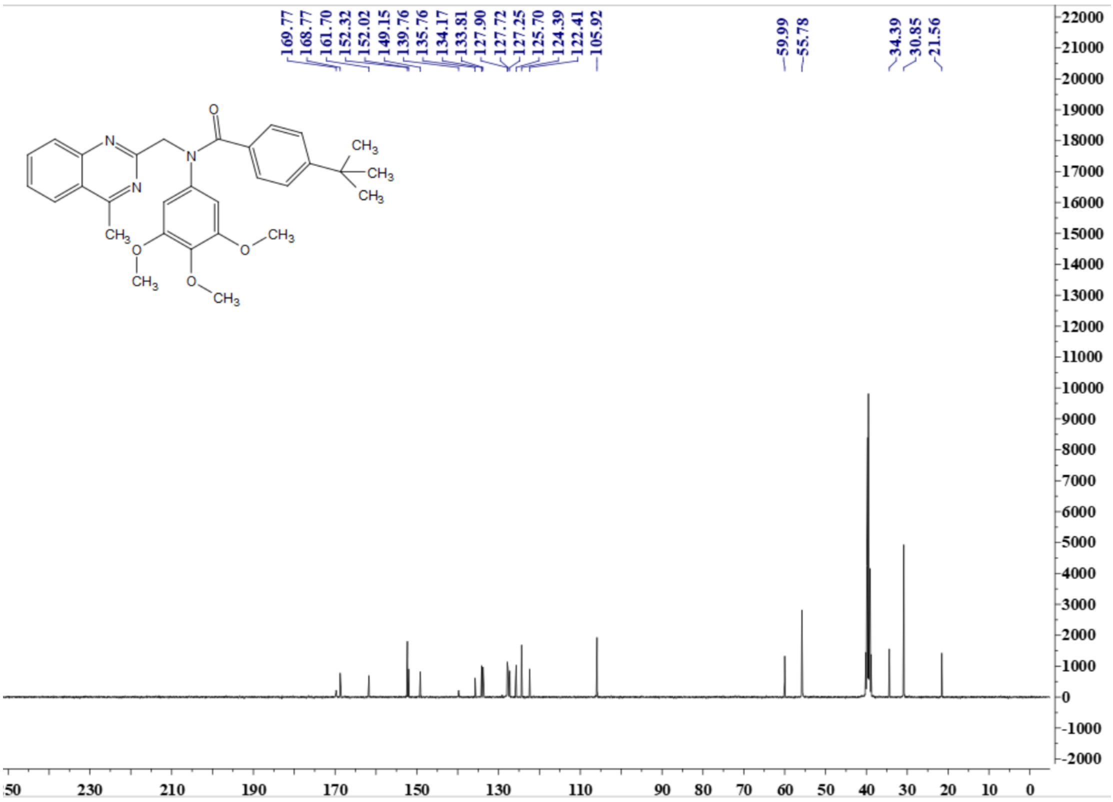

Figure S94. ^{13}C NMR spectrum of compound **11g** (100 MHz, DMSO-*d6*)

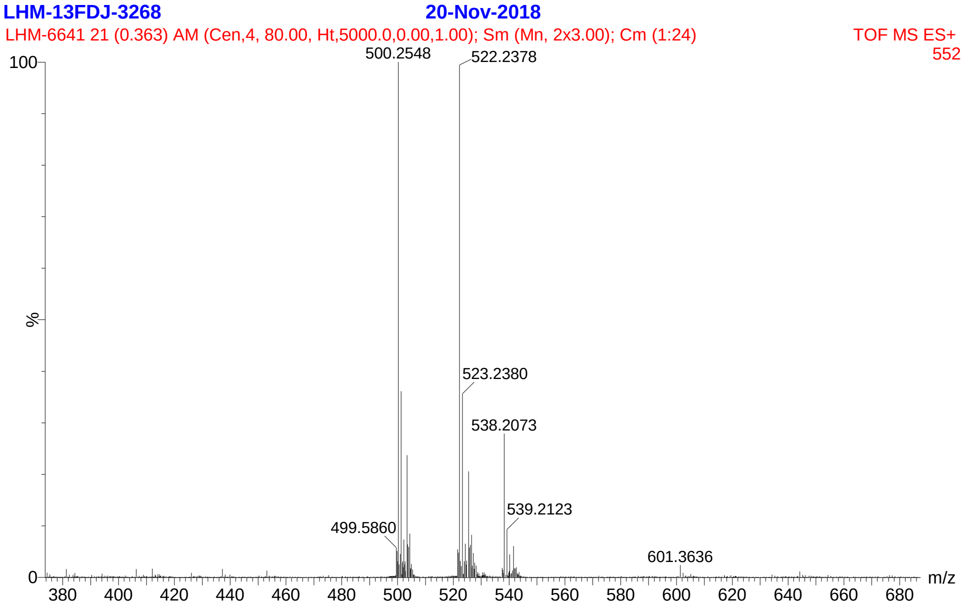

Figure S95. HRMS spectrum of compound **11g**

- ^{1}H, ^{13}C-NMR and HRMS of Compound **11h**

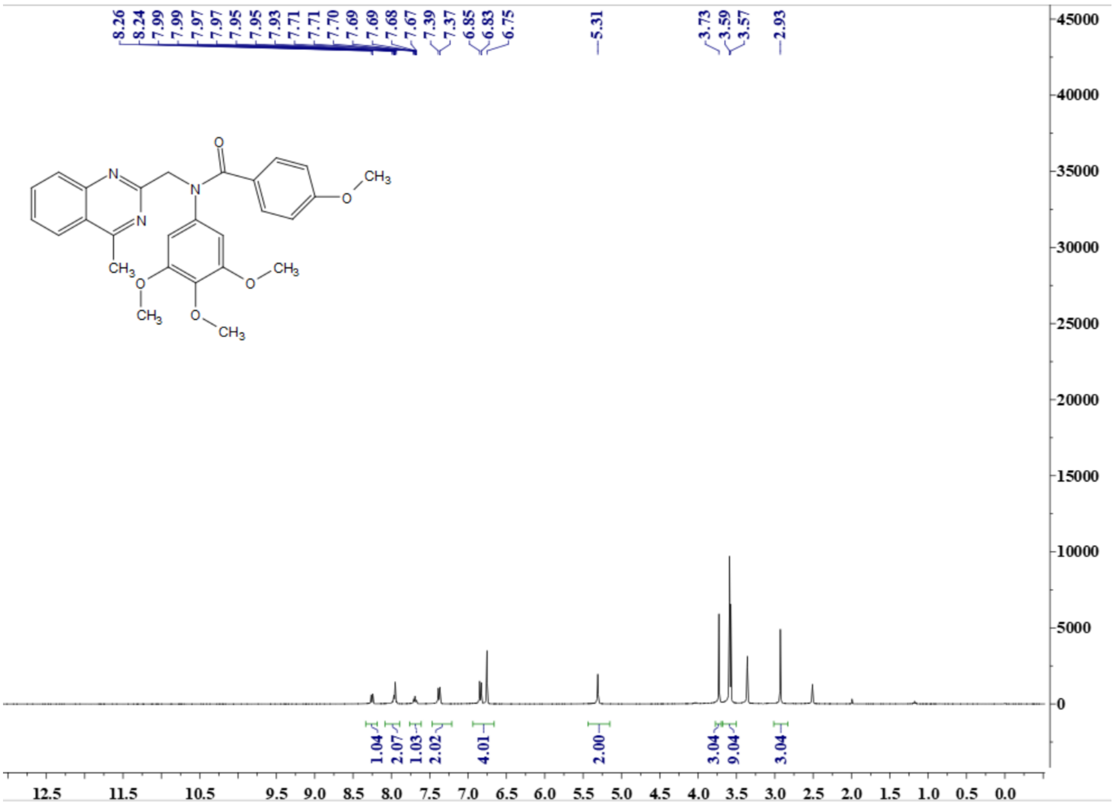

Figure S96. ^{1}H NMR spectrum of compound **11h** (400 MHz, DMSO-*d6*)

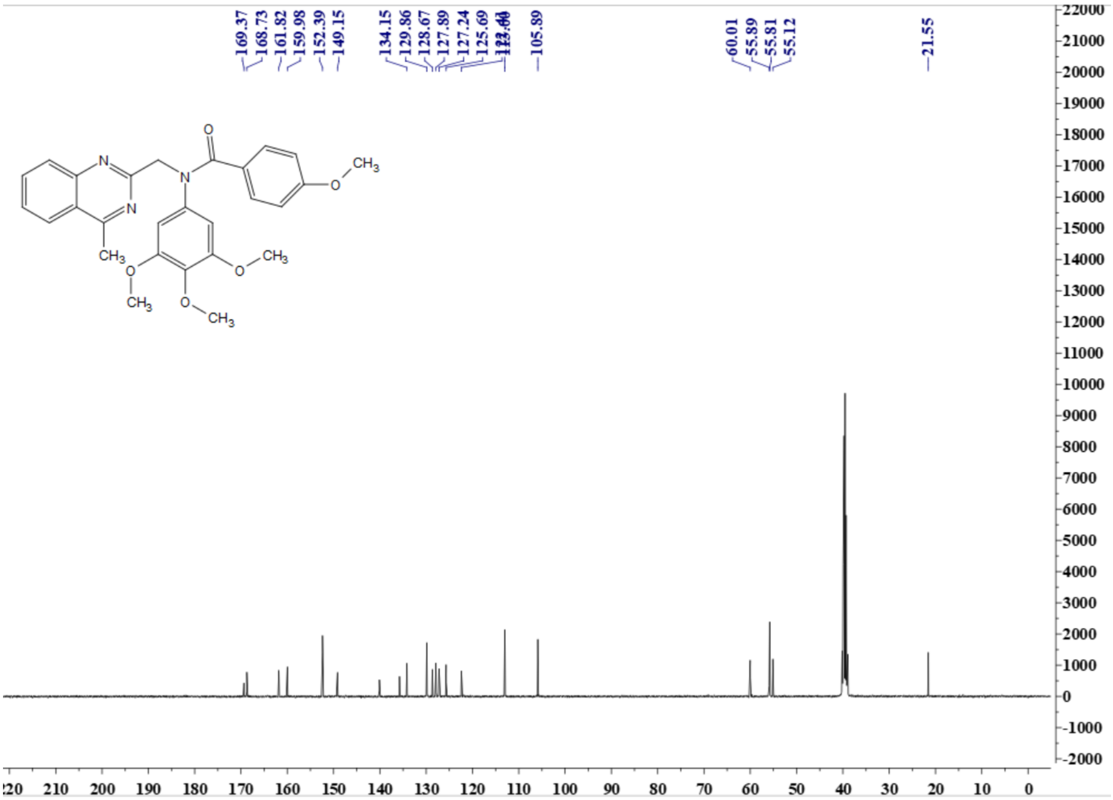

Figure S97. ^{13}C NMR spectrum of compound **11h** (100 MHz, DMSO-*d6*)

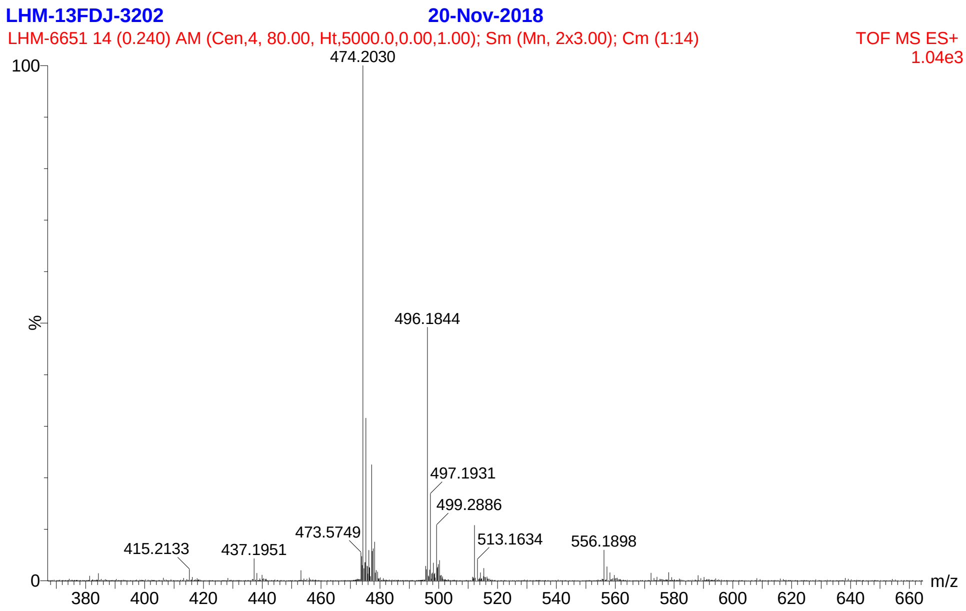

Figure S98. HRMS spectrum of compound **11h**

- ^{1}H, ^{13}C-NMR and HRMS of Compound **11i**

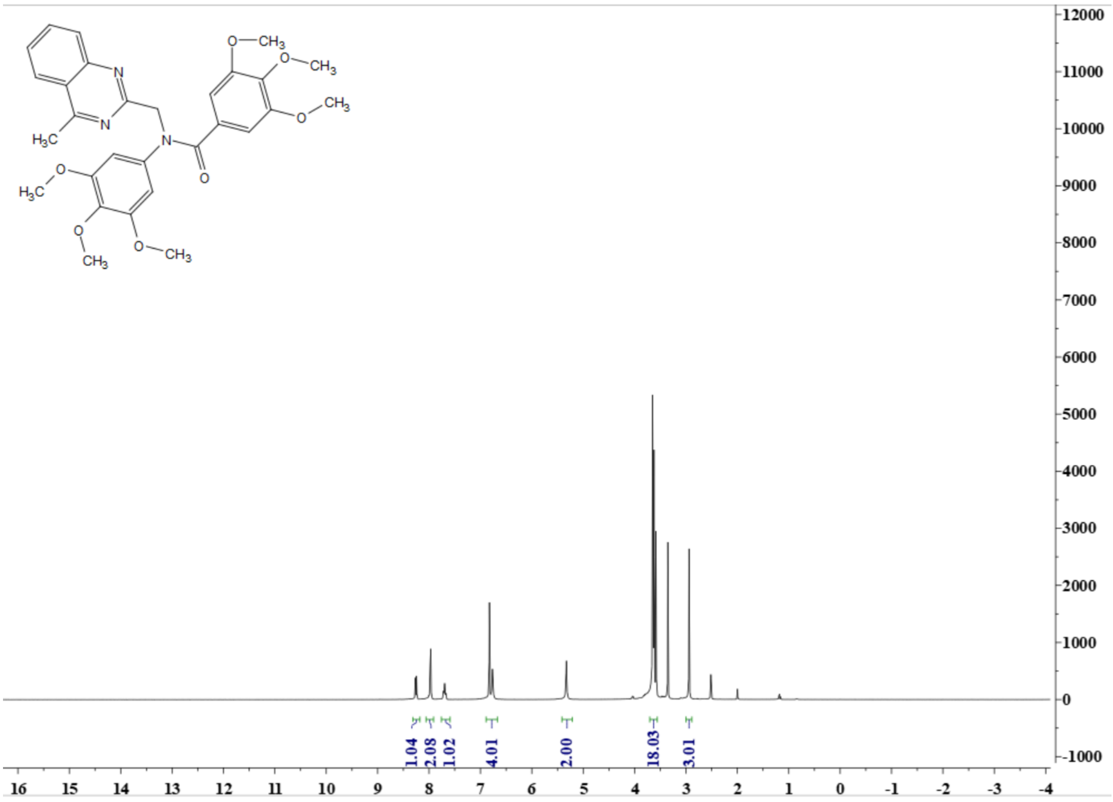

Figure S99. ^{1}H NMR spectrum of compound **11i** (400 MHz, DMSO-*d6*)

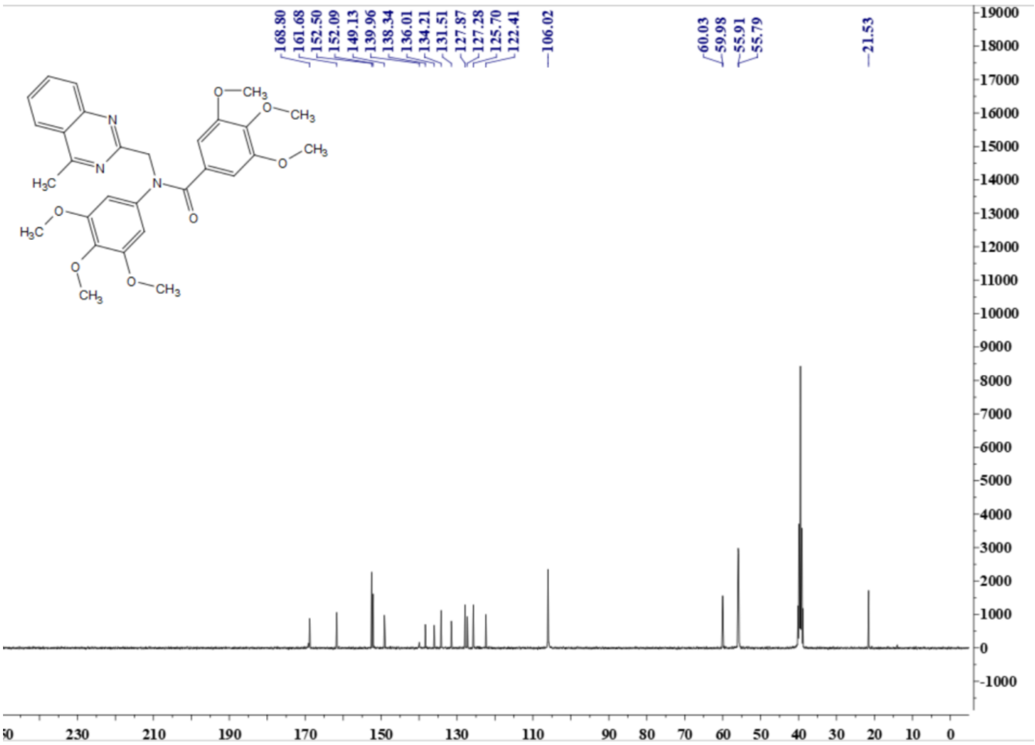

Figure S100. ^{13}C NMR spectrum of compound **11i** (100 MHz, DMSO-d_6)

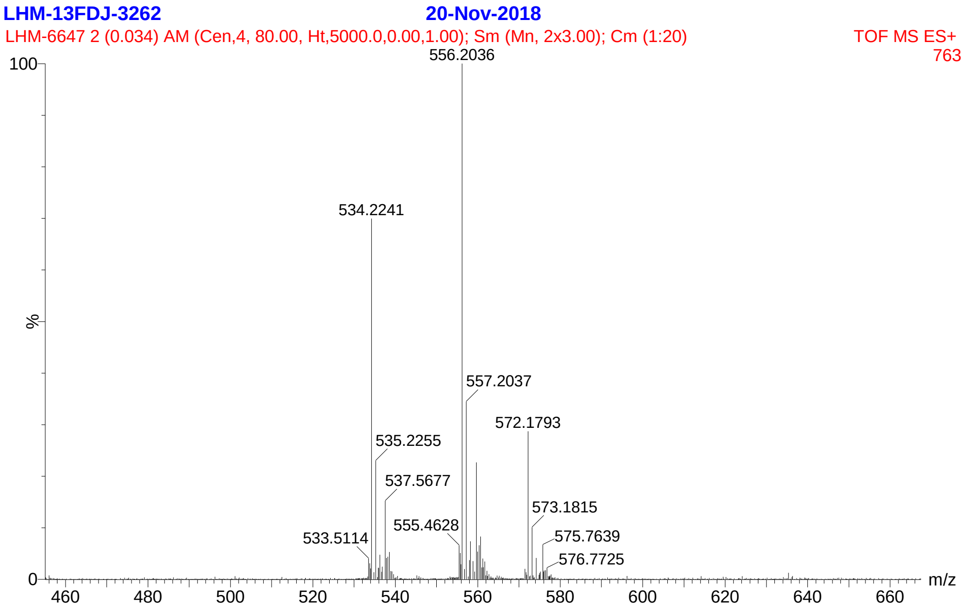

Figure S101. HRMS spectrum of compound **11i**

● ^{1}H, ^{13}C-NMR and HRMS of Compound **11j**

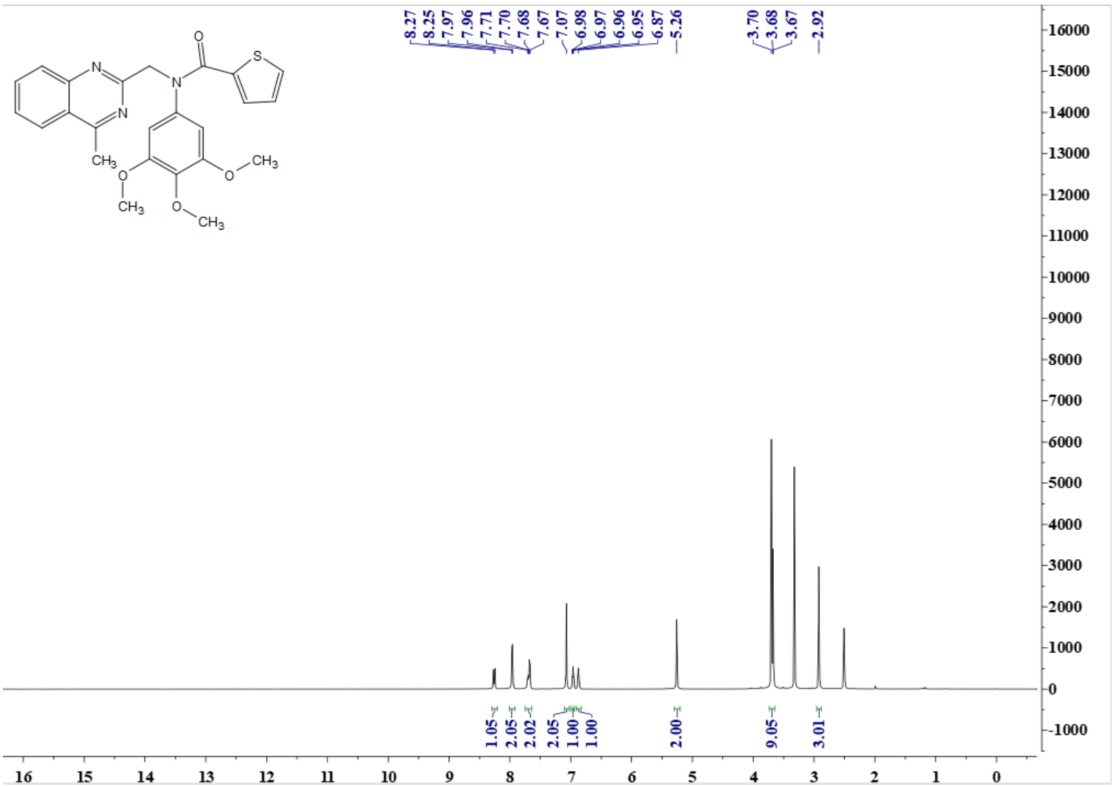

Figure S102. ^{1}H NMR spectrum of compound **11j** (400 MHz, DMSO-*d*6)

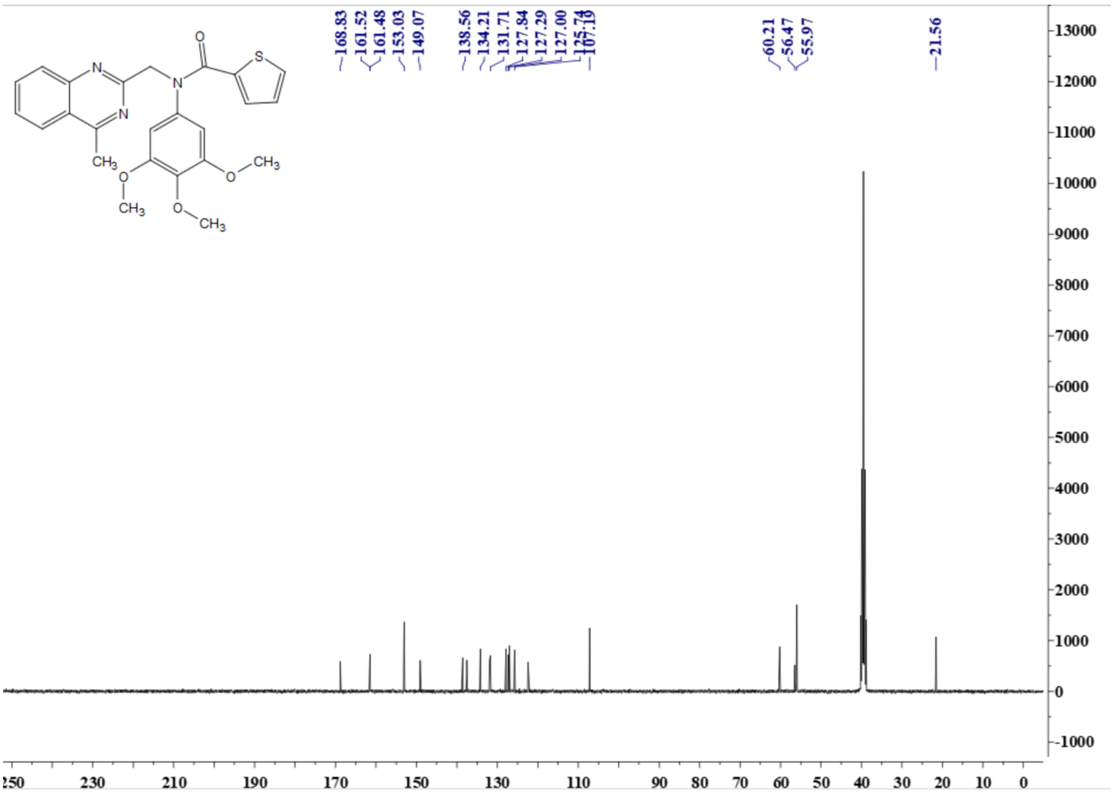

Figure S103. ^{13}C NMR spectrum of compound **11j** (100 MHz, DMSO-*d*6)

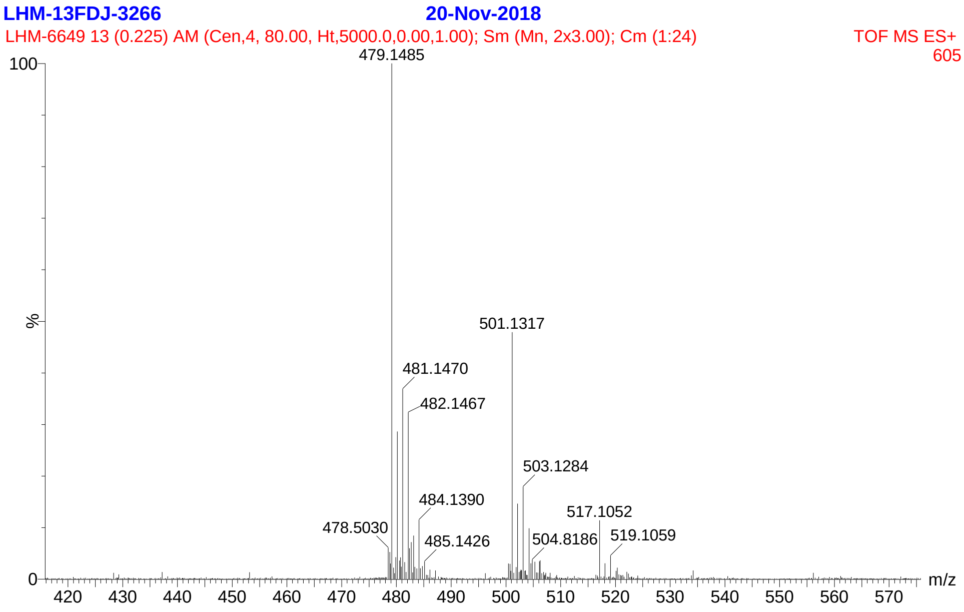

Figure S104. HRMS spectrum of compound **11j**

- ^{1}H, ^{13}C-NMR and HRMS of Compound **14a**

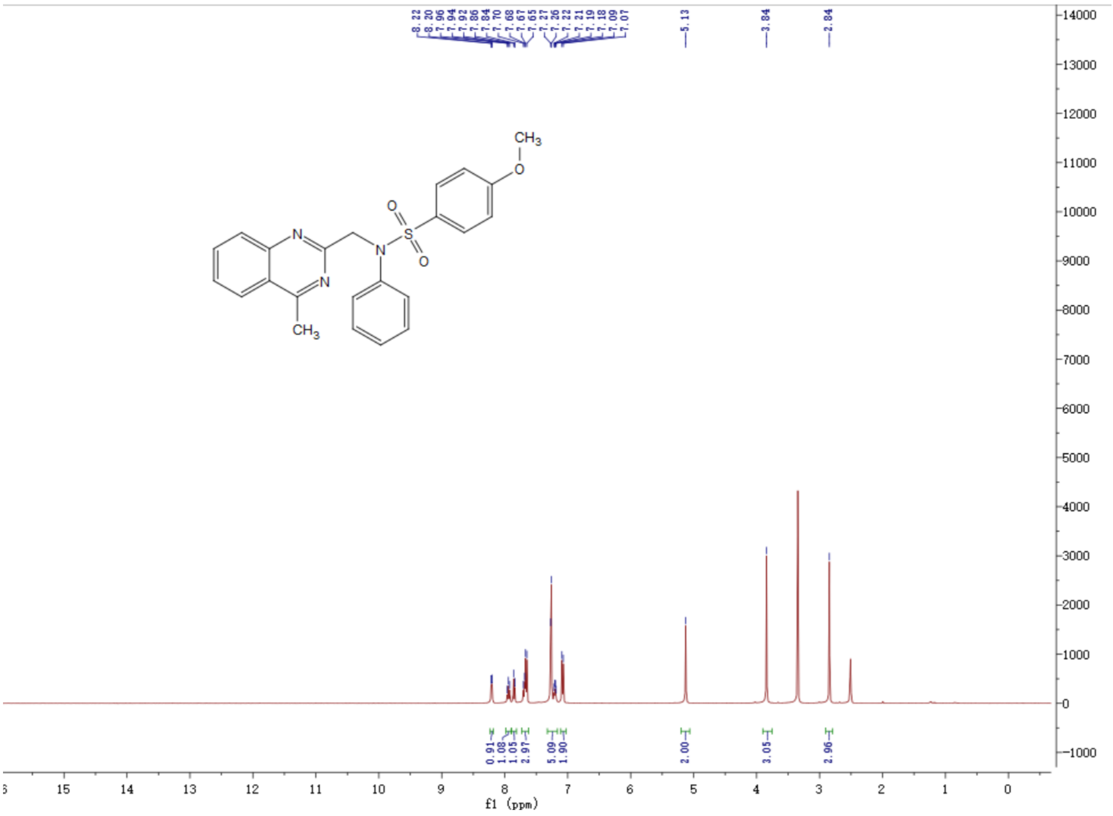

Figure S105. ^{1}H NMR spectrum of compound **14a** (400 MHz, DMSO-d_6)

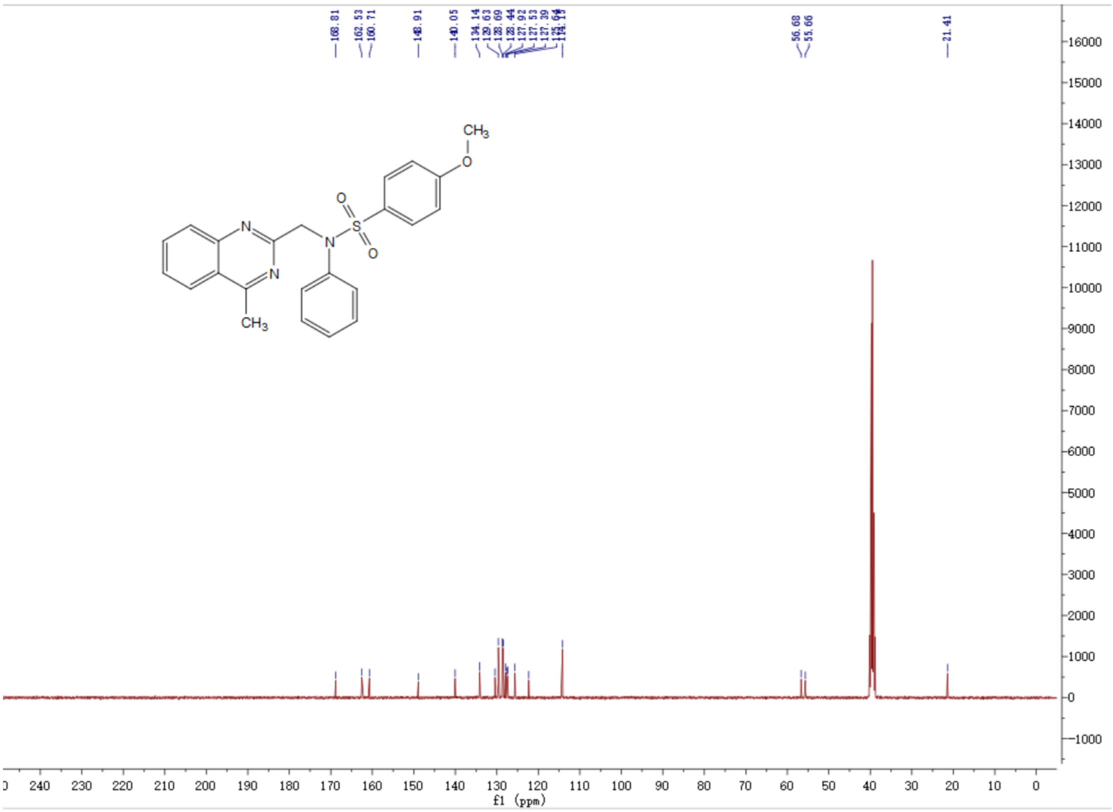

Figure S106. ^{13}C NMR spectrum of compound **14a** (100 MHz, DMSO-d_6)

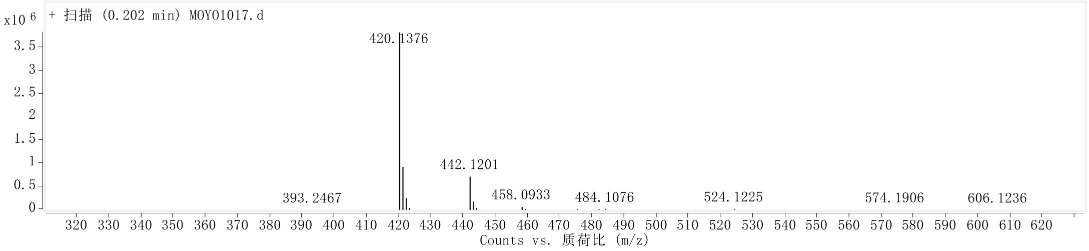

Figure S107. HRMS spectrum of compound **14a**

- ^{1}H, ^{13}C-NMR and HRMS of Compound **14b**

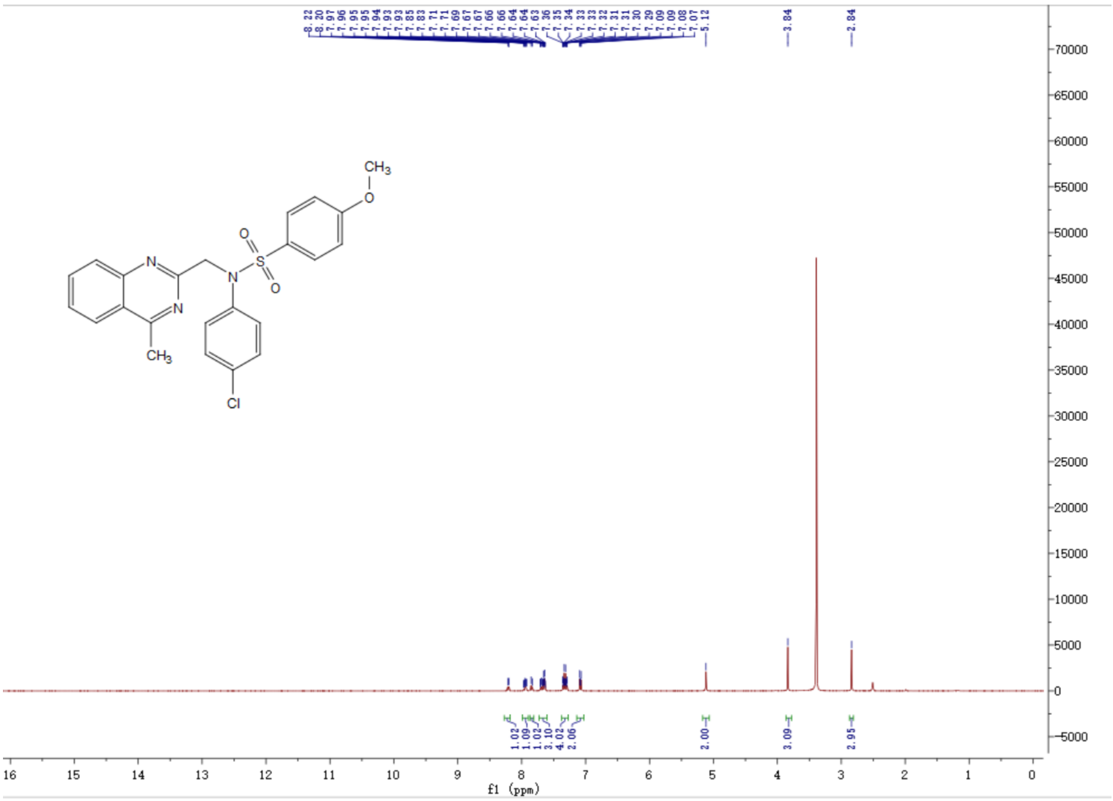

Figure S108. ^{1}H NMR spectrum of compound **14b** (400 MHz, DMSO-*d6*)

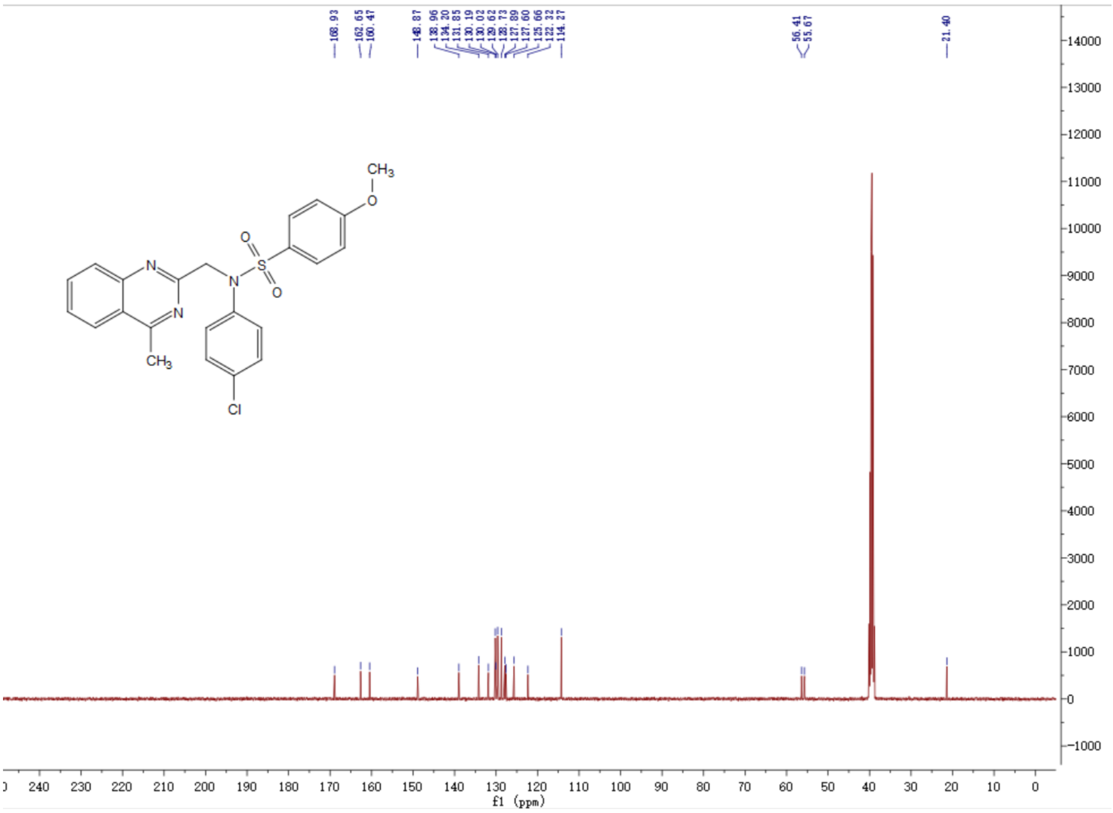

Figure S109. ^{13}C NMR spectrum of compound **14b** (100 MHz, DMSO-*d6*)

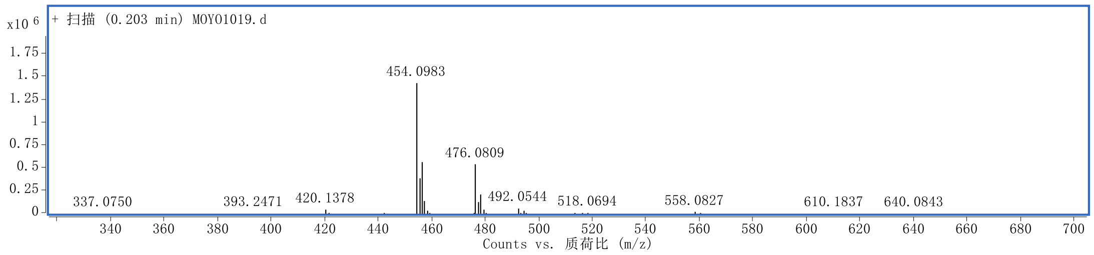

Figure S110. HRMS spectrum of compound **14b**

- ^{1}H, ^{13}C-NMR and HRMS of Compound **14c**

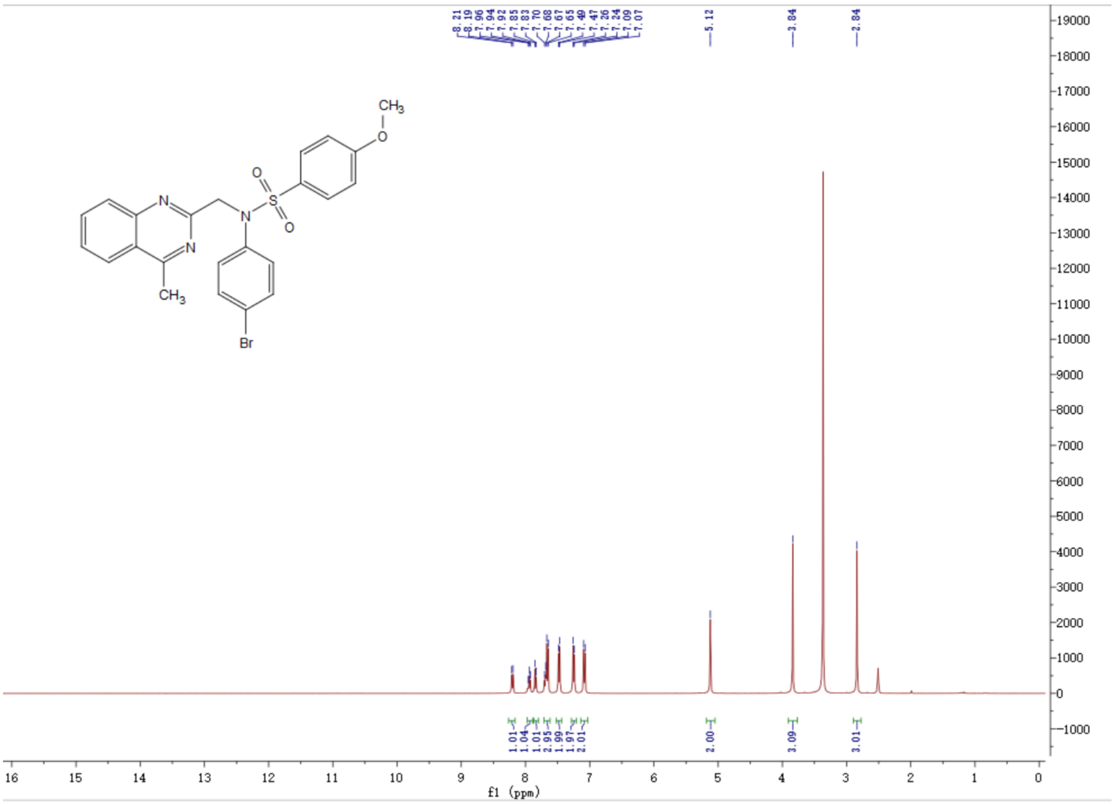

Figure S111. ^{1}H NMR spectrum of compound **14c** (400 MHz, DMSO-d_6)

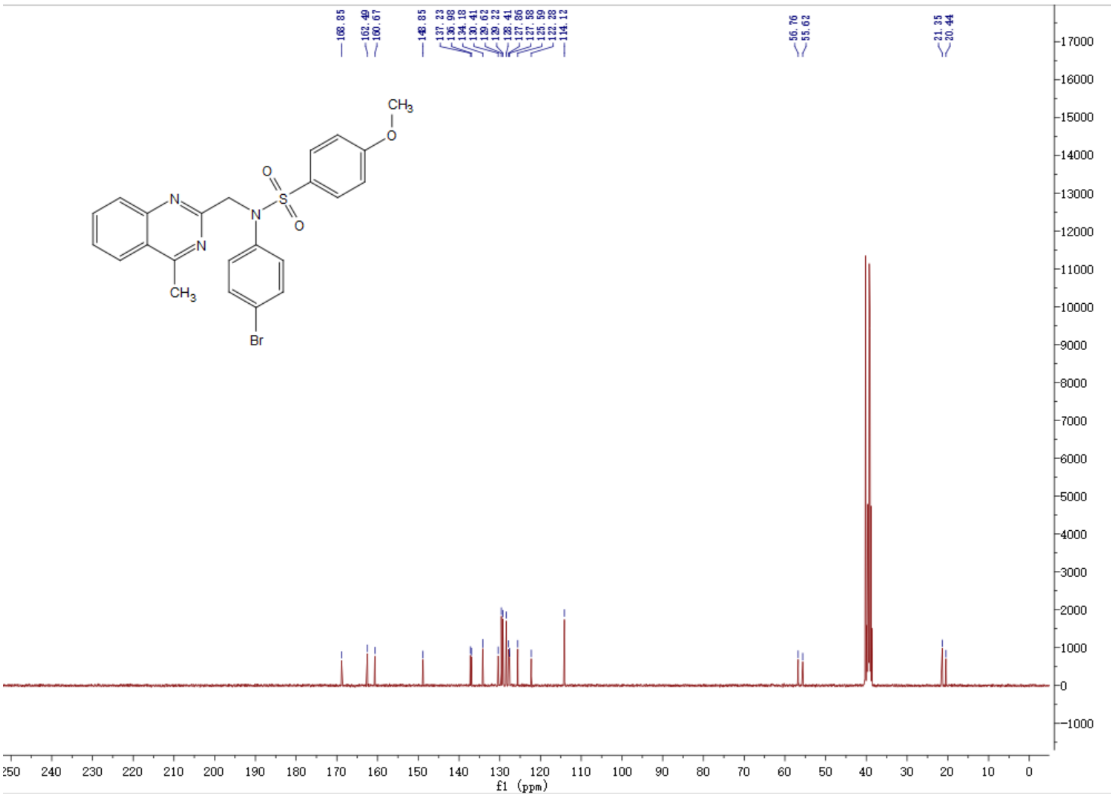

Figure S112. ^{13}C NMR spectrum of compound **14c** (100 MHz, DMSO-*d6*)

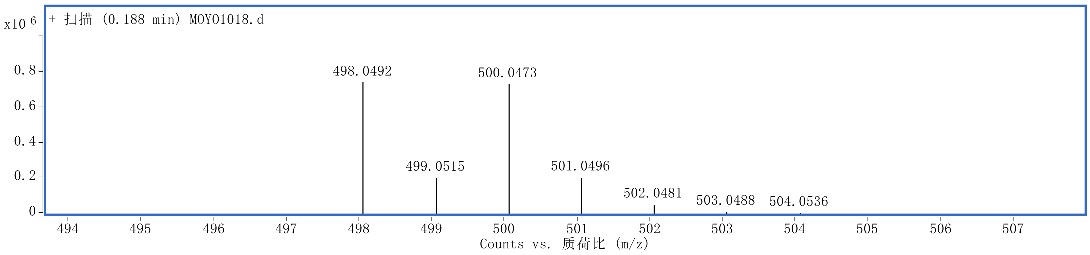

Figure S113. HRMS spectrum of compound **14c**

- ^{1}H, ^{13}C-NMR and HRMS of Compound **14d**

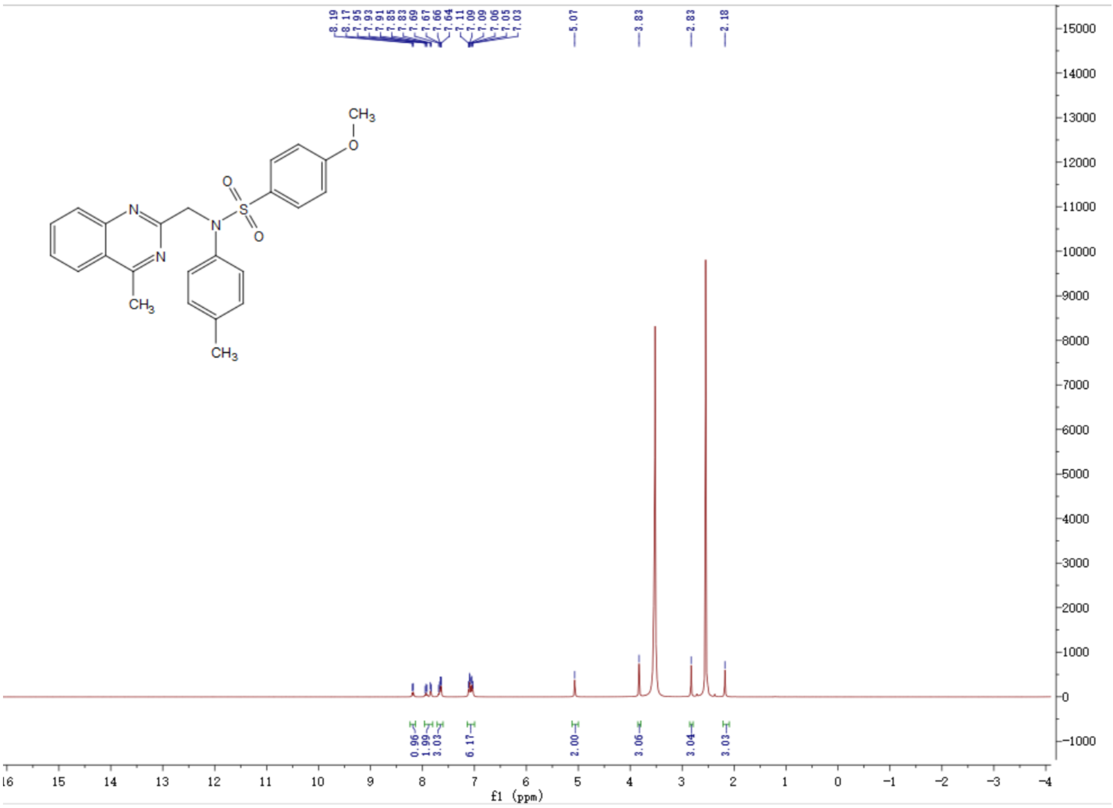

Figure S114. ^{1}H NMR spectrum of compound **14d** (400 MHz, DMSO-*d6*)

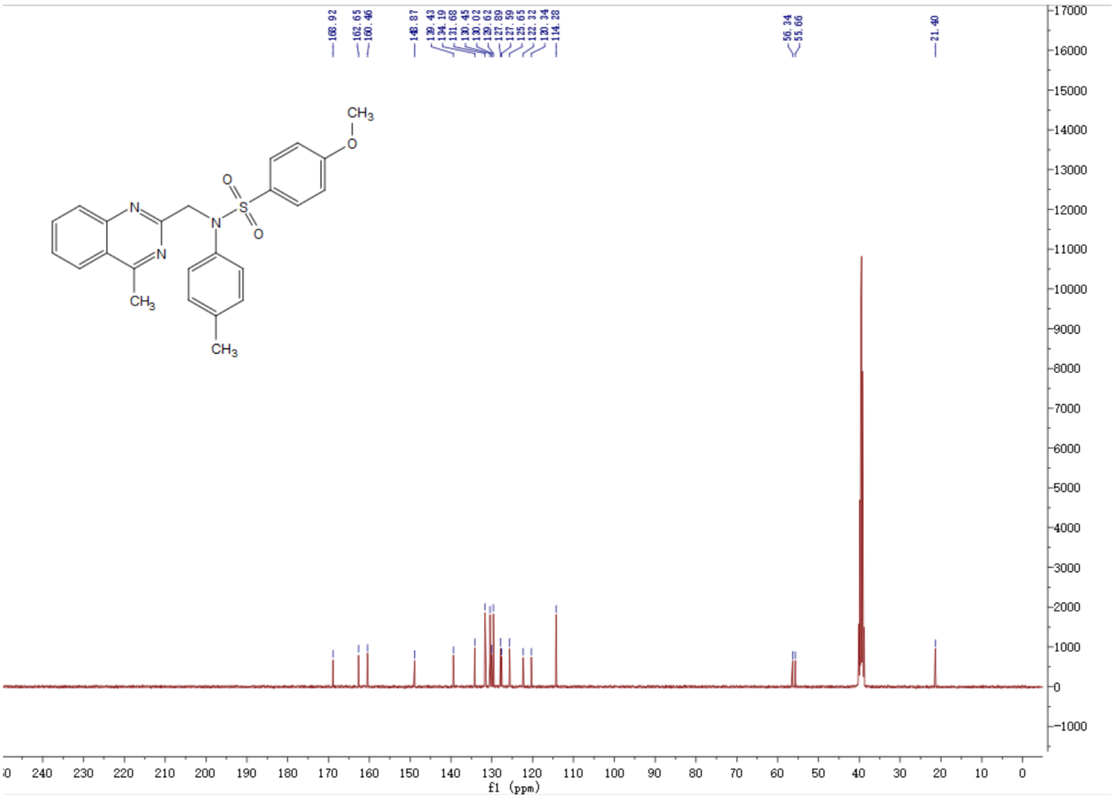

Figure S115. ^{13}C NMR spectrum of compound **14d** (100 MHz, DMSO-*d6*)

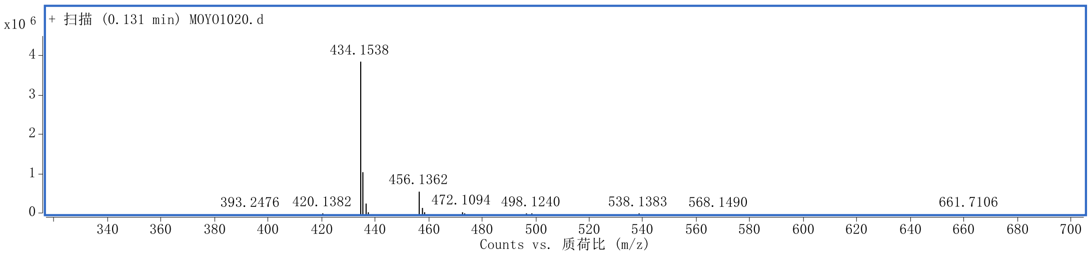

Figure S116. HRMS spectrum of compound **14d**
